# Efficacy of perioperative and neoadjuvant therapies in gastric and gastroesophageal junction adenocarcinoma: a network meta-analysis

**DOI:** 10.1093/oncolo/oyaf157

**Published:** 2025-08-04

**Authors:** Muhammad Umair Anjum, Syed Arsalan Ahmed Naqvi, Salman Ayub Jajja, Ammad Raina, Muhammad Umar Afzal, Kunwer Sufyan Faisal, Diana Segovia, Zhaohui Jin, Harry H Yoon, Pedro Luiz Serrano Uson Junior, Jason Starr, Daniel H Ahn, Tanios S Bekaii-Saab, Irbaz Bin Riaz, Mohamad Bassam Sonbol

**Affiliations:** Division of Hematology and Oncology, Mayo Clinic Cancer Center, Mayo Clinic, Phoenix, AZ, United States; Division of Hematology and Oncology, Mayo Clinic Cancer Center, Mayo Clinic, Phoenix, AZ, United States; Division of Hematology and Oncology, Mayo Clinic Cancer Center, Mayo Clinic, Phoenix, AZ, United States; Department of Internal Medicine, Canyon Vista Medical Center, Midwestern University, Sierra Vista, AZ, United States; Division of Hematology and Oncology, Mayo Clinic Cancer Center, Mayo Clinic, Phoenix, AZ, United States; Department of Internal Medicine, Ziauddin Medical University, Karachi, Pakistan; Division of Hematology and Oncology, Mayo Clinic Cancer Center, Mayo Clinic, Phoenix, AZ, United States; Division of Oncology, Mayo Clinic Cancer Center, Mayo Clinic, Rochester, MN, United States; Division of Oncology, Mayo Clinic Cancer Center, Mayo Clinic, Rochester, MN, United States; Center for Personalized Medicine, Hospital Israelita Albert Einstein, São Paulo, Brazil; Division of Oncology, Mayo Clinic Cancer Center, Mayo Clinic, Jacksonville, FL, United States; Division of Hematology and Oncology, Mayo Clinic Cancer Center, Mayo Clinic, Phoenix, AZ, United States; Division of Hematology and Oncology, Mayo Clinic Cancer Center, Mayo Clinic, Phoenix, AZ, United States; Division of Hematology and Oncology, Mayo Clinic Cancer Center, Mayo Clinic, Phoenix, AZ, United States; Division of Hematology and Oncology, Mayo Clinic Cancer Center, Mayo Clinic, Phoenix, AZ, United States

**Keywords:** gastric cancer, gastroesophageal junction cancer, perioperative, neoadjuvant, overall survival, disease-free survival

## Abstract

**Background:**

Optimal treatment for resectable gastric and gastroesophageal junction (GEJ) adenocarcinoma remains unclear due to limited head-to-head comparisons among chemotherapy and chemoradiation regimens. This network meta-analysis aimed to determine the relative efficacy of available multimodality treatment regimens in these patients.

**Methods:**

MEDLINE, EMBASE, Scopus, Web of Science, and CENTRAL were searched till September 20, 2024. Phase 3 randomized trials evaluating perioperative/neoadjuvant systemic therapy ± radiation in resectable gastric/GEJ adenocarcinoma were included. Primary outcomes were disease-free survival (DFS) and overall survival (OS). Frequentist network meta-analysis was conducted to compare hazard ratios (HRs) and 95% confidence intervals (CIs).

**Results:**

Fifteen trials (8072 patients) were analyzed. pFLOT (perioperative fluorouracil, leucovorin, oxaliplatin, and docetaxel) ranked highest for DFS in the overall population (*P*-score 91%), achieving significant improvements compared to surgery alone (HR 0.48, 95% CI, 0.38-0.60) and nCROSS (neoadjuvant paclitaxel, carboplatin, and radiotherapy) (0.67, 0.56-0.81). For OS, nPLF + nCRT(EP) (neoadjuvant cisplatin, leucovorin, and fluorouracil for induction, followed by etoposide, cisplatin, and radiotherapy) (*P*-score 90%) and pFLOT (*P*-score 87%) were the top regimens. pFLOT significantly outperformed surgery alone (0.57, 0.45-0.72) and nCROSS (0.73, 0.60-0.89). Based on the limited data available, adding neoadjuvant chemoradiation to pFLOT provided no additional OS (1.14, 0.76-1.72) or DFS (1.22, 0.83-1.82) benefit. Similarly, adding pembrolizumab to perioperative chemotherapy (cisplatin and fluorouracil/capecitabine) was not superior to pFLOT (DFS: 1.10, 0.71-1.69; OS: 1.09, 0.69-1.72).

**Conclusions:**

pFLOT demonstrated superior efficacy in resectable gastric/GEJ adenocarcinoma, outperforming surgery alone, nCROSS, and alternative perioperative regimens. The additive role of immunotherapy requires further investigation to optimize patient selection and outcomes.

**Implications for Practice**

The optimal treatment strategy for resectable gastric and gastroesophageal junction (GEJ) adenocarcinoma remains unclear. In our network meta-analysis of 15 trials, perioperative FLOT (fluorouracil, leucovorin, oxaliplatin, and docetaxel) has shown superior efficacy compared to alternative strategies, including the CROSS regimen (neoadjuvant paclitaxel and carboplatin with radiotherapy). Moreover, the addition of neoadjuvant chemoradiation to pECF/ECX or FLOT offered no additional survival benefit.

## Introduction

Gastroesophageal cancers are relatively uncommon in the United States, with fewer than 50,000 new cases expected in 2024.^1^ However, these cancers are responsible for significant morbidity and mortality worldwide.^2^ For early-stage gastric and gastroesophageal junction (GEJ) adenocarcinoma, the primary therapeutic goal is curative intent. Multimodality treatment strategies, such as neoadjuvant chemoradiotherapy and perioperative chemotherapy, are the recommended standard of care.^3-5^ The implementation of these approaches has significantly improved survival outcomes in this patient population.

The MAGIC trial^6^ was the first landmark trial to provide substantial evidence supporting the use of perioperative chemotherapy in treating patients with gastric and GEJ adenocarcinoma. The results showed significant improvement in overall survival (OS) and disease-free survival (DFS) in patients who received pECF (perioperative epirubicin, cisplatin, and fluorouracil) compared to surgical resection alone. While the MAGIC trial demonstrated benefits of perioperative chemotherapy, the CROSS trial^7^ later showed the superiority of neoadjuvant chemoradiotherapy using the nCROSS regimen (neoadjuvant paclitaxel and carboplatin with radiotherapy) over surgery alone. Building on these advancements, the FLOT4 trial^8^ more recently assessed the efficacy of the pFLOT protocol (perioperative fluorouracil, leucovorin, oxaliplatin, and docetaxel) compared to the widely accepted MAGIC-based regimen, finding significantly superior efficacy in both OS and DFS. Most recently, the phase 3 ESOPEC trial^9^ demonstrated a significant improvement in OS with pFLOT compared to nCROSS regimen in resectable esophageal adenocarcinoma. Additionally, recent trials such as KEYNOTE-585^10^ investigated the potential benefits of incorporating immunotherapy into perioperative regimens for resectable gastric and GEJ adenocarcinoma.

Despite all these advancements, the lack of head-to-head comparisons among these different regimens has led to uncertainty regarding the optimal treatment strategy for patients with resectable gastric and GEJ adenocarcinoma. As a result, the choice of regimen varies according to individual physician preferences in different geographical settings. This systematic review and network meta-analysis were conducted to evaluate all available therapies for resectable gastric and GEJ adenocarcinoma using direct and indirect comparisons, to identify the most efficacious therapy for improving survival.

## Methods

This systematic review and network meta-analysis are reported in accordance with the Preferred Reporting Items for Systematic Reviews and Meta-Analyses (PRISMA) extension statement for systematic reviews, incorporating network meta-analyses for healthcare interventions^11^ (Supplementary Methods 1). This study was registered in the Open Science Framework.^12^

### Literature search

Electronic databases, such as MEDLINE, EMBASE, Scopus, Web of Science, and Cochrane Central Register of Controlled Trials (CENTRAL), were comprehensively searched from each database’s inception till September 20, 2024. The search strategy was designed and conducted by an experienced librarian using controlled vocabulary and supplementary keywords after thorough discussion with the principal investigator. The detailed search strategy is available in Supplementary Methods 2.

### Study selection

We included phase 3 randomized controlled trials assessing the efficacy and safety of different perioperative systemic therapies ± radiation treatment in patients with localized gastric or lower esophageal/GEJ adenocarcinoma. Systemic therapy was defined as either chemotherapy or immune checkpoint inhibitors (ICIs). We included ICI trials due to their established role^13,14^ in metastatic gastric and GEJ cancers, recent FDA approvals for perioperative use in other cancers^15,16^ (eg, breast and lung), and emerging interest in their application in the perioperative setting, particularly following their role in the adjuvant setting^17^ after trimodality therapy. Trials evaluating anti-HER2 therapies were excluded because HER2-positive disease represents a distinct patient population with different therapeutic considerations. Similarly, anti-angiogenesis agents were not included as our primary interest lies in the evolving role of chemotherapy and ICIs in HER2-negative disease.

Trials reporting purely gastric or upper/upper to mid esophageal cancer without GEJ data were excluded. Trials allowing adjuvant treatment were included only if there was a neoadjuvant portion. Trials that reported data for only squamous cell carcinoma patients were also excluded. Trials that included a patient population of both adenocarcinoma and squamous cell carcinoma patients were only included if the majority of patients had adenocarcinoma or data for adenocarcinoma was reported separately. Nonrandomized, single-arm, phase 1, 2, or 4 studies were excluded.

Two independent reviewers (M.U.A. and S.A.J.) first screened the titles and abstracts of the retrieved articles, followed by a subsequent review of full text for potential inclusion in accordance with the *a priori* eligibility criteria. Any discrepancies or conflicts between the two reviewers were resolved after discussion with a third reviewer (M.B.S.).

### Data extraction and quality assessment

Data from the final list of eligible trials were extracted using a systematically structured data collection instrument. Extracted data included, but not limited to, trial characteristics, baseline population characteristics, and outcome results. Additionally, the quality of included trials was assessed using the Cochrane risk of bias tool version 2.^18^

The process of data extraction and risk of bias assessment was carried out by 2 independent reviewers (M.U.A. and S.A.J.). Any discrepancies or conflicts between the 2 reviewers were resolved by consensus or by input from a third reviewer (M.B.S.).

### Outcomes

Main patient-important efficacy outcomes included DFS and OS. Additionally, R0 resection rate and pathologic complete response (pCR) were assessed. Safety outcomes included overall grade 3 and higher adverse events (G3AE) and specific G3AEs including diarrhea, nausea, vomiting, neutropenia, neutropenic fever, infections, and peripheral neuropathy.

### Statistical analysis

For survival outcomes (OS and DFS), precomputed hazard ratios (HRs) with corresponding 95% CIs were pooled using an inverse variance approach after logarithmic transformation. Raw binary outcome data (ie, pCR) were expressed as relative risk with 95% CI. Mixed treatment comparisons were made using network meta-analyses within the frequentist framework. The choice of model was based on the geometry of the network and sparsity of direct evidence. Fixed-effect model was selected if the network was open with sparse direct evidence, since the common between-study heterogeneity cannot be estimated reliably in such networks.^19-21^ For each patient-important outcome, relative treatment rankings were assessed using a *P*-score and evaluated for their congruence with pairwise estimates. A higher relative treatment rank indicated a better potential for efficacy and safety.

Given the sparsity of direct evidence leading to multiple subnetworks, clinically relevant assumptions were formulated for equivalent efficacy among different treatment options. The relative efficacy of pCF (perioperative cisplatin with fluorouracil) was assumed to be similar to that of pCF/CX (perioperative cisplatin with fluorouracil/capecitabine)^22,23^, which was the comparator in the KEYNOTE-585 trial.^10^ Likewise, the relative efficacy of nCF (neoadjuvant cisplatin with fluorouracil) was assumed to be similar to that of nPLF (neoadjuvant cisplatin, leucovorin, and fluorouracil), which was the comparator in the POET trial.^24^ To evaluate the robustness of this assumption, sensitivity analyses, excluding the POET trial, were conducted to assess OS in both the overall population and the GEJ cohort, and DFS in the overall population.

Additional sensitivity analyses were conducted, stratifying by treatment combination (pECF/ECX [perioperative epirubicin, cisplatin, and fluorouracil/capecitabine], and pFLOT separately), as assessed in the TOPGEAR trial.^25^

### Certainty of evidence

The grading of recommendation, assessment, development, and evaluation (GRADE) approach^26^ was used to assess the certainty of evidence.^19^ Each outcome was assessed for overall risk of bias, indirectness, inconsistency, and publication bias using direct evidence. Indirect evidence was evaluated for intransitivity, while imprecision and incoherence were assessed in network estimates. A noncontextualized approach with a null effect as the threshold was used to assess imprecision.^21^ The findings were graded as high, moderate, low, or very low and presented in a summary table.

## Results

### Study selection

Of the total 1639 studies identified up until September 20, 2024, 15 clinical trials (18 references)^6-10,24,25,27-37^ were included in this systematic review and network meta-analysis as shown in Supplementary Figure 1.

### Study characteristics

A total of 8072 patients were included in the 15 trials (Table 1; Supplementary Table 1). The median age ranged from 62 to 63 years (interquartile range) across these trials. Twelve trials^6,8-10,24,25,29,32,33,35-37^ included patients with adenocarcinoma only, and 3 trials^27,30,34^ had a mixed population with both adenocarcinoma and squamous cell carcinoma patients. All trials included and reported data for patients with lower esophageal/GEJ cancer. Additionally, gastric and upper/middle esophageal cancer patients were also included, in addition to GEJ cancer, in 8^6,8,10,25,32,34,35,37^ and 4^27,30,33,34,36^ trials, respectively.

**Table 1.** Baseline trial characteristics.

Trial	Arm	*N*	Cycles of therapy used	Radiation dose (Gy)	Age in years median (range)
Perioperative setting					
FLOT4^8^	pFLOT	356	4 cycles before and 4 cycles after surgery	Not applicable	62 (54-69)
	pECF/ECX	360	3 cycles before and 3 cycles after surgery	Not applicable	62 (52-69)
KEYNOTE-585^10^	pPembro + CF/CX	402	3 cycles of combined therapy before and 3 cycles of combined therapy after, followed by up to 11 cycles of pembrolizumab alone	Not applicable	64 (56-70)
	Placebo + pCF/CX	402	3 cycles of combined therapy before and 3 cycles of combined therapy after, followed by up to 11 cycles of placebo alone	Not applicable	63 (55-69)
MAGIC^6^	pECF	250	3 cycles before and 3 cycles after surgery	Not applicable	62 (29-85)
	Surgery alone	253	NA	Not applicable	62 (23-81)
FNCLCC/FFCD Study^32^	pCF	113	2/3 cycles before and 3/4 cycles after surgery	Not applicable	63 (36-75)
	Surgery alone	111	NA	Not applicable	63 (38-75)
MATTERHORN^35^	pDurva + FLOT	474	2 cycles of durvalumab and FLOT before and after surgery, followed by 10 cycles of durvalumab alone	Not applicable	NA
	Placebo + pFLOT	474	2 cycles of placebo and FLOT before and after surgery, followed by 10 cycles of placebo alone	Not applicable	NA
TOPGEAR^25^	pECF/ECX/FLOT + nCRT(F/X)	286	3 cycles before and 3 cycles after surgery (ECF/ECX); 4 cycles before and 4 cycles after surgery (FLOT)	45	61 (11) ^a^
	pECF/ECX/FLOT	288	3 cycles before and 3 cycles after surgery (ECF/ECX); 4 cycles before and 4 cycles after surgery (FLOT)	Not applicable	60 (11) ^a^
CRITICS^37^	nECX + aCRT(CX)	395	3 cycles before surgery (ECX), followed by chemoradiotherapy post-surgery	45	63 (56-68) ^b^
	pECX	393	3 cycles before and 3 cycles after surgery	Not applicable	62 (54-69) ^b^
Neoadjuvant setting					
CROSS^7^	nCROSS	178	5 cycles before surgery	41.4	60 (36-79)
	Surgery alone	188	NA	Not applicable	60 (36-73)
Burmeister BH et al^30^	nCRT(CF)	128	NA	35	61 (41-80)
	Surgery alone	128	NA	Not applicable	62 (28-83)
POET^24^	nPLF + nCRT(EP)	60	2 cycles of PLF and 3 cycles of concurrent chemoradiotherapy before surgery	30	60.6
	nPLF	59	2.5 cycles before surgery	Not applicable	56
UK MRC OE05^33^	nECX	446	4 cycles before surgery	Not applicable	62 (33-80)
	nCF	451	2 cycles before surgery	Not applicable	62 (27-81)
MRC OE02^34^	nCF	400	2 cycles before surgery	Not applicable	63 (36-84)
	Surgery alone	402	NA	Not applicable	62 (30-80)
EA2174^36^	nCROSS + Nivo	137	5 cycles before surgery	41.4-50.4	65.5
	nCROSS	138	5 cycles before surgery	41.4-50.4	
Perioperative with neoadjuvant setting					
Neo-AEGIS^29^	pECF ^c^	184	4 cycles before and 4 cycles after surgery	Not applicable	63.8
	nCROSS	178	5 cycles before surgery	41.4	63.8
ESOPEC^9^	pFLOT	221	4 cycles before and 4 cycles after surgery	Not applicable	63.1 (8.6) ^a^
	nCROSS	217	5 cycles before surgery	41.4	62.6 (9.8) ^a^

Abbreviations: *N*, number of patients; pFLOT, perioperative fluorouracil, leucovorin, oxaliplatin, and docetaxel; pECF/ECX, perioperative epirubicin, cisplatin, and fluorouracil/capecitabine; pPembro + CF/CX, perioperative pembrolizumab with cisplatin and fluorouracil/capecitabine; pCF/CX, perioperative cisplatin and fluorouracil/capecitabine; pECF, perioperative epirubicin, cisplatin, and fluorouracil; pCF, perioperative cisplatin and fluorouracil; pDurva + FLOT, perioperative durvalumab with fluorouracil, leucovorin, oxaliplatin and docetaxel; pECF/ECX/FLOT + nCRT(F/X): (either perioperative epirubicin with cisplatin and fluorouracil/capecitabine, or perioperative fluorouracil, leucovorin, oxaliplatin, and docetaxel, along with neoadjuvant chemoradiotherapy (fluorouracil/capecitabine and radiotherapy); pECF/ECX/FLOT, perioperative epirubicin, cisplatin and fluorouracil/capecitabine, or fluorouracil, leucovorin, oxaliplatin, and docetaxel; nECX + aCRT(CX), neoadjuvant epirubicin, cisplatin/oxaliplatin and capecitabine, followed by adjuvant chemoradiotherapy with radiotherapy, cisplatin and capecitabine; pECX, perioperative epirubicin, cisplatin/oxaliplatin and capecitabine; nCROSS, neoadjuvant paclitaxel and carboplatin with radiotherapy; nCRT(CF), neoadjuvant cisplatin and fluorouracil with radiotherapy; nPLF + nCRT (EP), neoadjuvant cisplatin, leucovorin, and fluorouracil for induction, followed by etoposide and cisplatin with concomitant radiotherapy; nPLF, neoadjuvant cisplatin, leucovorin, and fluorouracil; nECX, neoadjuvant epirubicin, cisplatin, and capecitabine; nCF, neoadjuvant cisplatin and fluorouracil; nCROSS + nivo, neoadjuvant paclitaxel and carboplatin with radiotherapy, plus nivolumab; NA, not available.

^a^Mean (standard deviation) reported.

^b^Median (interquartile range) reported.

^c^pECF was the predominant choice of chemotherapy administered in this group. However, 27 (15%) patients in the perioperative chemotherapy group received pFLOT instead after a protocol amendment.

Five trials^8,10,25,35,37^ compared different perioperative systemic therapies, 3 trials^24,33,36^ compared neoadjuvant therapies, 2 trials^9,29^ compared neoadjuvant therapy with perioperative therapy, and 5 trials^6,27,30,32,34^ compared either neoadjuvant or perioperative therapy with surgery alone. Two trials^10,35^ were blinded, with the remainder being open label. The primary endpoint was OS in all the trials except the Burmeister et al.,^30^ MATTERHORN,^35^ and EA2174^36^ trials.

The nCROSS^9,27,29,36^ treatment regimen was assessed in four trials, while pFLOT^8,9,35^ was assessed in three trials. nCF^33,34^ and pECF^6,29^ were each assessed in two trials. Other comparators included: pPembro + CF/CX^10^ (perioperative pembrolizumab with cisplatin and fluorouracil/capecitabine), pECF/ECX,^8^ pCF/CX, nCRT(CF)^30^ (neoadjuvant cisplatin and fluorouracil with radiotherapy), nPLF + nCRT(EP)^24^ (neoadjuvant cisplatin, leucovorin, and fluorouracil for induction, followed by etoposide and cisplatin with concomitant radiotherapy), nPLF,^24^ nECX^33^ (neoadjuvant epirubicin, cisplatin, and capecitabine), pCF,^32^ pECF/ECX/FLOT + nCRT(F/X)^25^ (either perioperative ECF, ECX, or FLOT, along with neoadjuvant chemoradiotherapy [fluorouracil/capecitabine and radiotherapy]), pECF/ECX/FLOT,^25^ pECX^37^ (perioperative epirubicin, cisplatin/oxaliplatin, and capecitabine), nECX + aCRT(CX)^37^ (nECX followed by adjuvant chemoradiotherapy with radiotherapy, cisplatin, and capecitabine), nCROSS + Nivo^36^ (nCROSS with nivolumab), and pDurva + FLOT^35^ (perioperative durvalumab with pFLOT). Surgery alone^6,27,30,32,34^ was used as a comparator in 5 trials. Detailed patient characteristics are shown in Table 1 and Supplementary Table 1. Of note, while pECF/ECX was the main chemotherapy used in the Neo-AEGIS trial,^29^ pFLOT was used in 15% of the patients. Similarly, in TOPGEAR trial,^25^ two-thirds of the patients in each group received ECF/ECX chemotherapy, and one-third received FLOT.

Overall, the included trials had a low risk of bias for OS, with some concerns for DFS and pCR (potential detection bias due to open-label nature of these trials) (Supplementary Figure 2).

### Network meta-analysis

#### Disease-free survival

Thirteen trials^6-10,25,29-34,37^ were included in the network for DFS in the overall population (Supplementary Figures 3–5).

**Figure 1.**
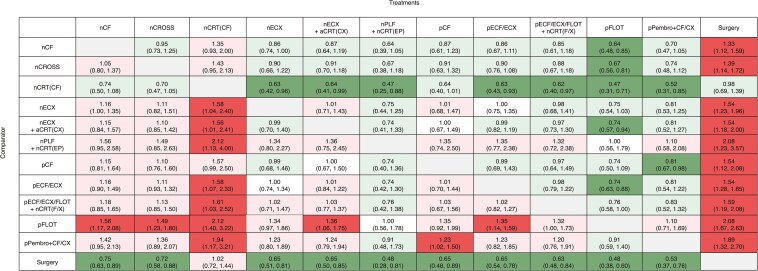
League table showing mixed treatment comparisons in the overall population for disease-free survival. The values in each cell represent the relative treatment effect (and 95% CI) of the treatment on the top, compared to the treatment on the left. Green color suggests relative treatment benefit. Light green suggests nonsignificant benefit and dark green suggests significant benefit. Red color suggests relative treatment harm. Light red suggests nonsignificant harm, and dark red suggests significant harm.

In the overall population (gastric/GEJ adenocarcinoma), mixed treatment comparisons for DFS showed that pFLOT (rank-1, *P*-score 91%) was ranked as potentially the most efficacious treatment, followed by nPLF + nCRT(EP) (rank-2, *P*-score 84%), and pPembro + CF/CX (rank-3, *P*-score 81%) (Supplementary Figure 6). The pFLOT regimen was associated with a statistically significant improvement in DFS compared to nCF (HR 0.64, 95% CI, 0.48-0.85), nCROSS (0.67, 0.56-0.81), nCRT(CF) (0.47, 0.31-0.71), nECX + aCRT(CX) (0.74, 0.57-0.94), pECF/ECX (0.74, 0.63-0.88), and surgery alone (0.48, 0.38-0.60) (Figure 1). nPLF + nCRT(EP) was associated with a statistically significant improvement in DFS compared to nCRT(CF) (0.47, 0.25-0.88) and surgery alone (0.48, 0.28-0.81). Additionally, pPembro + CF/CX was associated with a statistically significant DFS improvement compared to nCRT(CF) (0.52, 0.31-0.85), pCF (0.81, 0.67-0.98), and surgery alone (0.53, 0.37-0.76). However, no statistically significant associations were observed among pFLOT, nPLF + nCRT(EP), and pPembro + CF/CX in terms of DFS improvement. Detailed results of other mixed treatment comparisons are reported in Figure 1 and Supplementary Figure 6. The addition of neoadjuvant chemoradiation to pFLOT (pECF/ECX/FLOT + nCRT[F/X]) did not improve DFS compared to pFLOT alone (1.32, 1.00-1.73). Sensitivity analyses with data for TOPGEAR trial stratified according to each treatment combination administered (pECF/ECX or pFLOT) showed consistent results as reported in Supplementary Figure 7. Another sensitivity analysis, excluding the POET trial,^31^ also showed consistent results as reported in Supplementary Figure 8.

Due to the formation of multiple subnetworks, POET^31^ and KEYNOTE-585^10^ trials were excluded from the analysis for DFS in the GEJ cohort. The five remaining trials^9,25,28-30^ were included in the network as shown in Supplementary Figure 9. In the GEJ cohort, mixed treatment comparisons for DFS showed that pECF/ECX/FLOT + nCRT(F/X) (rank-1, *P*-score 90%) and pFLOT (rank-2, *P*-score 88%) were ranked as potentially the most efficacious treatments (Supplementary Figure 10). The pECF/ECX/FLOT + nCRT(F/X) treatment combination was associated with a statistically significant improvement in DFS compared to nCRT(CF) (0.46, 0.25-0.84), and surgery alone (0.44, 0.26-0.75) (Supplementary Figure 10). pFLOT was associated with statistically significant improvement in DFS compared to nCROSS (0.66, 0.51-0.85), nCRT(CF) (0.47, 0.28-0.78), and surgery alone (0.46, 0.31-0.67). No statistically significant association was observed between pFLOT and pECF/ECX + nCRT in terms of DFS improvement. Detailed results of other mixed treatment comparisons are reported in Supplementary Figure 10.

#### Overall survival:

Thirteen^6,8-10,25,28-34,37^ and 11^6,8-10,25,28,29,31,32,34,37^ trials contributed to the network (Supplementary Figures 11–13) for OS in the overall population and the GEJ cohort, respectively.

**Figure 2.**
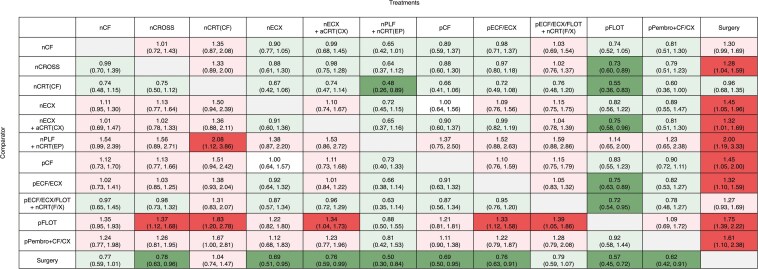
League table showing mixed treatment comparisons in the overall population for overall survival. The values in each cell represent the relative treatment effect (and 95% CI) of the treatment on the top, compared to the treatment on the left. Green color suggests relative treatment benefit. Light green suggests nonsignificant benefit and dark green suggests significant benefit. Red color suggests relative treatment harm. Light red suggests nonsignificant harm, and dark red suggests significant harm.

In the overall population of gastric/GEJ adenocarcinoma, mixed treatment comparisons for OS showed that nPLF + nCRT(EP) (rank-1, *P*-score 90%) and pFLOT (rank-2, *P*-score 87%) were ranked as potentially the most efficacious treatments (Supplementary Figure 14, Figure 2). pFLOT was associated with statistically significant improvement in OS compared to nCROSS (0.73, 0.60-0.89), nCRT(CF) (0.55, 0.36-0.83), nECX + aCRT(CX) (0.75, 0.58-0.96), pECF/ECX (0.75, 0.63-0.89) and surgery alone (0.57, 0.45-0.72). pPembro + CF/CX was associated with statistically significant improvement in OS compared to surgery alone (0.62, 0.42-0.91). However, no statistically significant associations were observed among pFLOT, nPLF + nCRT(EP), and pPembro + CF/CX in terms of OS improvement.

Sensitivity analyses with data for TOPGEAR trial stratified according to each treatment combination administered showed that pFLOT + nCRT(F/X) (rank-4, *P*-score 64%) ranked lower than pFLOT and was not superior to pFLOT in the mixed treatment comparisons (1.14, 0.76-1.72) (Supplementary Figure 15).

Detailed results of other mixed treatment comparisons are reported in Figure 2 and Supplementary Figures 14 and 15.

In the GEJ cohort, mixed treatment comparisons showed that nPLF + nCRT(EP) (rank-1, *P*-score 85%) and pFLOT (rank-2, *P*-score 79%) (Supplementary Figure 17) were ranked as potentially the most efficacious treatments in terms of OS. However, the nPLF + nCRT(EP) treatment regimen was only associated with statistically significant improvement in OS compared to surgery alone (0.47, 0.28-0.79) (Supplementary Figure 17). On the other hand, pFLOT was associated with statistically significant OS improvement in comparison with nCROSS (0.71, 0.57-0.89), pECF/ECX (0.75, 0.60-0.92), and surgery alone (0.54, 0.40-0.75). Detailed results of other mixed treatment comparisons are reported in Supplementary Figure 17.

Sensitivity analyses, excluding POET trial,^31^ showed consistent results in the overall population and GEJ cohort, as reported in Supplementary Figures 16 and 18, respectively.

#### Pathologic complete response

Due to the formation of multiple subnetworks, UK MRCOE05^33^ and KEYNOTE-585^10^ trials were excluded from the analysis for pCR. Five^9,25,29,35,36^ remaining trials were included in the network as shown in Supplementary Figure 19.

In the overall population, mixed treatment comparisons showed that pDurva + FLOT (rank-1, *P*-score 100%) (Supplementary Figure 20) was ranked as potentially the most efficacious treatment in terms of improving pCR. The pDurva + FLOT regimen was associated with a statistically significant increase in pCR when compared to nCROSS (RR 3.85, 95% CI, 2.08-7.14), nCROSS + Nivo (3.23, 1.54-6.67), pECF/ECX (10.64, 3.80-29.79), pECF/ECX/FLOT + nCRT(F/X) (5.06, 1.59-16.14), and pFLOT (2.68, 1.84-3.89) (Supplementary Figure 20). Of note, the pDurva + FLOT regimen of the MATTERHORN trial^25^ was not included in the DFS and OS analyses as these results have not been reported yet. Detailed results of other mixed treatment comparisons are reported in Supplementary Figure 20.

#### Adverse events

Given the heterogeneity of different treatment regimens and their toxicities, adverse events data were reported descriptively without comparative analysis. Data from overall and specific G3AEs, including diarrhea, nausea, vomiting, neutropenia, neutropenic fever, infections and peripheral neuropathy, are reported in Supplementary Table 2.

### Certainty of evidence

Summary of findings for the pairwise comparisons of pFLOT and nCROSS with the other treatment regimens in the overall population and the GEJ cohort is provided in Supplementary Tables 3 and 4. Overall, the certainty of evidence ranged from low (due to imprecision) to high.

## Discussion

In this systematic review and network meta-analysis of patients with locoregional esophageal/GEJ adenocarcinoma, we found that pFLOT showed OS and DFS benefit compared to surgery, nCROSS regimen, and pECF/ECX. The addition of neoadjuvant chemoradiation (nCRT) to perioperative chemotherapy (pECF/ECX or pFLOT) was not superior to pFLOT alone. These results were also consistent when restricting the analysis to the GEJ patients alone.

The FLOT4 trial^8^ established pFLOT as the standard in gastric and GEJ cancers. However, questions remained about whether a perioperative strategy is superior to the trimodality CROSS regimen. The CROSS trial^7^ included esophageal (including squamous) and GEJ Siewert types 1 and 2 patients, but excluded Siewert type 3. In contrast, the FLOT 4 trial^8^ encompassed GEJ Siewert types 1, 2, and 3, as well as gastric adenocarcinomas. This led to the acceptance of a trimodality approach for esophageal cancers and a perioperative strategy for gastric cancers, leaving GEJ adenocarcinoma (Siewert type 3) as an unresolved overlap.

The Neo-AEGIS trial^29^ attempted to address this question but yielded inconclusive results, as most patients did not receive pFLOT. The ESOPEC trial,^9^ however, demonstrated the superiority of pFLOT over the trimodality approach. Our network meta-analysis corroborates these findings, confirming pFLOT as superior to other strategies.

Although pFLOT was clearly superior to the trimodality approach, the question remained of whether adding neoadjuvant chemoradiation to perioperative chemotherapy (ie, FLOT) would offer additional benefit. The TOPGEAR trial^25^ attempted to address this question by comparing chemoradiation plus chemotherapy with chemotherapy alone but fell short for 2 critical reasons. First, the chemotherapy regimens used in TOPGEAR^25^ were primarily pECF/ECX, not pFLOT, which is now the standard. Second, the concurrent chemotherapy in the chemoradiation arm was fluoropyrimidine monotherapy rather than carboplatin and paclitaxel in the nCROSS regimen. The results from our network meta-analysis demonstrated no added benefit of combining chemoradiation with pECF/ECX or pFLOT in gastric or GEJ adenocarcinoma (Figures 1 and 2). Additionally, subgroup analysis from the TOPGEAR trial, stratified by choice of chemotherapy, showed consistent findings, with no added benefit from the addition of chemoradiation to pFLOT (Supplementary Figures 7 and 15). However, this analysis could not isolate the GEJ cohort due to insufficient data and limited sample size.

The question of whether a CROSS-like regimen combined with pFLOT would yield superior outcomes remains unanswered. Until such data are available, the current evidence supports pFLOT as the standard of care for GEJ adenocarcinoma. Notably, our network meta-analysis also showed the non-conventional approach of using nPLF + nCRT(EP) to be amongst the top-performing regimens in terms of both OS and DFS in the gastric and GEJ adenocarcinoma population. However, these findings should be interpreted with caution due to the small sample size, early closure of the trial, and differences in treatment protocols. Recognizing these caveats, we conducted a sensitivity analysis excluding the POET trial, which demonstrated consistent results, thereby reinforcing the robustness of our main conclusions.

While the superiority of pFLOT is well-established, the role of immunotherapy in earlier treatment settings warrants further exploration. ICIs in combination with first-line chemotherapy are the current standard of care^38^ for patients with PD-L1-positive metastatic GEJ/gastric adenocarcinoma, but their role in earlier settings remains unclear. The CheckMate 577 trial^17^ demonstrated a DFS benefit for adjuvant nivolumab in patients who underwent trimodality therapy and did not achieve pCR. However, OS data from this trial are still awaited. As the trial population in CheckMate 577^17^ differs from those included in our study, Checkmate 577 was not included in this analysis. In contrast, we included two trials evaluating perioperative ICIs (KEYNOTE-585^10^ and MATTERHORN^35^), which investigated the addition of pembrolizumab or durvalumab to chemotherapy, respectively. Perioperative pembrolizumab, as evaluated in KEYNOTE-585, did not show a significant OS or DFS benefit compared to CF/CX alone. In our network meta-analysis, indirect comparison between pembrolizumab + CF/CX (from KEYNOTE-585) and pFLOT (from FLOT4 and ESOPEC) similarly did not demonstrate a significant survival advantage for pembrolizumab-based therapy over pFLOT. Meanwhile, durvalumab added to pFLOT demonstrated a pCR benefit compared to pFLOT alone, though it remains uncertain whether this will translate into a survival advantage, as full results from MATTERHORN^35^ are still pending. It is important to note that pCR as a surrogate endpoint for OS has come under increasing scrutiny.^39^ Several trials, including Neo-AEGIS,^29^ TOPGEAR,^25^ and KEYNOTE-585,^10^ have reported pCR improvements that failed to translate into OS benefits. This underscores the need for future research to refine patient selection and identify subgroups that may benefit from immunotherapy in earlier settings. For now, pFLOT remains the standard of care, supported by level-1 evidence.

There are several strengths of our study. First, our network meta-analysis not only confirms the findings of the ESOPEC trial,^9^ which demonstrated the superiority of pFLOT over nCROSS, but also provides additional insights into the evolving treatment landscape. Second, by synthesizing data from multiple trials, our analysis offers broader generalizability and reinforces the robustness of ESOPEC’s conclusions. Third, our inclusion of data from the TOPGEAR trial^25^ allows for a more nuanced evaluation of treatment strategies, particularly by comparing pFLOT with chemoradiation-based regimens. Although TOPGEAR^25^ predominantly utilized pECF/ECX rather than pFLOT, its findings help address gaps in the literature by exploring the impact of adding neoadjuvant chemoradiation to perioperative chemotherapy. Finally, we used GRADE to assess the certainty of evidence, formally appraising the evidence from mixed treatment comparisons.

The limitations of this study primarily arise from the use of trial-level data, and the small number of trials included at the level of each pairwise comparison. A large number of trials with individual patient data meta-analysis can potentially offer additional insights, adjusting for potential covariates. Moreover, there was a sparsity of direct evidence, which led to an open geometry of the network precluding formal assessment of incoherence. Likewise, publication bias at the level of mixed treatment comparisons could not be formally assessed due to limited direct evidence and sample sizes for these comparisons. Some included studies had patients with mixed pathologies (squamous cell carcinoma and adenocarcinoma) and different cancer subtypes (GEJ and gastric). However, we conducted additional analyses utilizing data from subgroup analyses in such trials, which showed consistent direction of results. Likewise, follow-up durations varied across the included trials, and hence, the relative efficacy of some treatments might be over- or underestimated. Despite these challenges, we believe this study offers meaningful insights into the evolving treatment landscape for GEJ adenocarcinoma.

## Conclusions

In summary, the treatment landscape for resectable GEJ adenocarcinoma continues to evolve, with pFLOT emerging as the standard of care due to its demonstrated superiority in OS and DFS compared to alternative strategies, including the nCROSS regimen, as shown in both the ESOPEC trial^9^ and this network meta-analysis. The addition of ICIs, as evaluated in studies such as KEYNOTE-585^10^ and MATTERHORN,^35^ has not yet demonstrated a clear survival benefit. Future efforts should focus on exploring the role of novel combinations and refining patient selection to further optimize outcomes in resectable GEJ adenocarcinoma.

## Supplementary Material

oyaf157_suppl_Supplementary_Figures_1-20_Tables_1-4

## Data Availability

The data underlying this article will be shared on reasonable request to the corresponding author.
