# Supplementary material for: Efficacy of perioperative and neoadjuvant therapies in gastric and gastroesophageal junction adenocarcinoma: a network meta-analysis
Source: Oncologist. 2025 Aug 4;30(8):oyaf157. doi: 10.1093/oncolo/oyaf157 (PMC12320486; doi:10.1093/oncolo/oyaf157)
Supplement: oyaf157_suppl_Supplementary_Figures_1-20_Tables_1-4 [file oyaf157_suppl_supplementary_figures_1-20_tables_1-4.docx]

**Online-only Supplemental Material**

[**Supplementary Methods 1: PRISMA checklist** 3](#_Toc196654614)

[**Supplementary Methods 2: Search strategy** 4](#_Toc196654615)

[**Supplementary Figure 1: PRISMA flow diagram showing the screening and selection process** 6](#_Toc196654616)

[**Supplementary Figure 2: Risk of bias in the included studies** 7](#_Toc196654617)

[**Supplementary Figure 3: Network plot for disease-free survival in the overall population** 9](#_Toc196654618)

[**Supplementary Figure 4: Network plot for disease-free Survival in the overall population, with data for TOPGEAR trial stratified according to each treatment combination administered** 10](#_Toc196654619)

[**Supplementary Figure 5: Network plot for disease-free survival in the overall population, excluding the POET trial** 11](#_Toc196654620)

[**Supplementary Figure 6: Mixed treatment comparisons for disease-free survival in the overall population** 12](#_Toc196654621)

[**Supplementary Figure 7: Mixed treatment comparisons for disease-free survival in the overall population, with data for TOPGEAR trial stratified according to each treatment combination administered** 13](#_Toc196654622)

[**Supplementary Figure 8: Mixed treatment comparisons for disease-free survival in the overall population, excluding the POET trial** 14](#_Toc196654623)

[**Supplementary Figure 9: Network plot for disease-free survival in the GEJ cohort, excluding the POET and KEYNOTE 585 trials** 15](#_Toc196654624)

[**Supplementary Figure 10: Mixed treatment comparisons for disease-free survival in the GEJ cohort, excluding the POET and KEYNOTE 585 trials** 16](#_Toc196654625)

[**Supplementary Figure 11: Network plot for overall survival in the overall population (left) and the GEJ cohort (right)** 17](#_Toc196654626)

[**Supplementary Figure 12: Network plot for overall survival in the overall population, with data for TOPGEAR trial stratified according to each treatment combination administered** 18](#_Toc196654627)

[**Supplementary Figure 13: Network plot for overall survival in the overall population (left) and the GEJ cohort (right), excluding the POET trial** 19](#_Toc196654628)

[**Supplementary Figure 14: Mixed treatment comparisons for overall survival in the overall population** 20](#_Toc196654629)

[**Supplementary Figure 15: Mixed treatment comparisons for overall survival in the overall population, with data for TOPGEAR trial stratified according to each treatment combination administered** 21](#_Toc196654630)

[**Supplementary Figure 16: Mixed treatment comparisons for overall survival in the overall population, excluding the POET trial** 22](#_Toc196654631)

[**Supplementary Figure 17: Mixed treatment comparisons for overall survival in the GEJ cohort** 23](#_Toc196654632)

[**Supplementary Figure 18: Mixed treatment comparisons for overall survival in the GEJ cohort excluding the POET trial** 24](#_Toc196654633)

[**Supplementary Figure 19: Network plot for pathologic complete response in the overall population, excluding the UK MRCOE05 and KEYNOTE-585 trials** 25](#_Toc196654634)

[**Supplementary Figure 20: Mixed treatment comparisons for pathologic complete response in the overall population, excluding the UK MRCOE05 and KEYNOTE-585 trials** 26](#_Toc196654635)

[**Supplementary Table 1: Additional patient characteristics** 27](#_Toc196654636)

[**Supplementary Table 2: Summary of adverse events** 29](#_Toc196654637)

[**Supplementary Table 3: GRADE summary of findings table outlining certainty of evidence and absolute risks with nCROSS and pFLOT compared to other treatment regimens for overall survival and disease-free survival in the overall population** 30](#_Toc196654638)

[**Supplementary Table 4: GRADE summary of findings table outlining certainty of evidence and absolute risks with nCROSS and pFLOT compared to other treatment regimens for overall survival and disease-free survival in the GEJ cohort** 31](#_Toc196654639)

# **Supplementary Methods 1: PRISMA checklist**

| **Section/Topic** | **Item #** | **Checklist Item** | **Reported on Page #** |
| --- | --- | --- | --- |
| **TITLE** |  |  |  |
| Title | 1 | Identify the report as a systematic review *incorporating a network meta-analysis (or related form of meta-analysis).* | 1 |
| **ABSTRACT** |  |  |  |
| Structured summary | 2 | Provide a structured summary including, as applicable:  **Background:** main objectives  **Methods:** data sources; study eligibility criteria, participants, and interventions; study appraisal; and *synthesis methods, such as network meta-analysis.*  **Results:** number of studies and participants identified; summary estimates with corresponding confidence/credible intervals; *treatment rankings may also be discussed. Authors may choose to summarize pairwise comparisons against a chosen treatment included in their analyses for brevity.*  **Discussion/Conclusions:** limitations; conclusions and implications of findings.  **Other:** primary source of funding; systematic review registration number with registry name. | 3-4 |
| **INTRODUCTION** |  |  |  |
| Rationale | 3 | Describe the rationale for the review in the context of what is already known*, including mention of why a network meta-analysis has been conducted.* | 5-6 |
| Objectives | 4 | Provide an explicit statement of questions being addressed, with reference to participants, interventions, comparisons, outcomes, and study design (PICOS). | 5-6 |
| **METHODS** |  |  |  |
| Protocol and registration | 5 | Indicate whether a review protocol exists and if and where it can be accessed (e.g., Web address); and, if available, provide registration information, including registration number. | 7 |
| Eligibility criteria | 6 | Specify study characteristics (e.g., PICOS, length of follow-up) and report characteristics (e.g., years considered, language, publication status) used as criteria for eligibility, giving rationale. *Clearly describe eligible treatments included in the treatment network and note whether any have been clustered or merged into the same node (with justification).* | 7-8 |
| Information sources | 7 | Describe all information sources (e.g., databases with dates of coverage, contact with study authors to identify additional studies) in the search and date last searched. | 7 |
| Search | 8 | Present full electronic search strategy for at least one database, including any limits used, such that it could be repeated. | Supplement |
| Study selection | 9 | State the process for selecting studies (i.e., screening, eligibility, included in systematic review, and, if applicable, included in the meta-analysis). | 7-8 |
| Data collection process | 10 | Describe method of data extraction from reports (e.g., piloted forms, independently, in duplicate) and any processes for obtaining and confirming data from investigators. | 8 |
| Data items | 11 | List and define all variables for which data were sought (e.g., PICOS, funding sources) and any assumptions and simplifications made. | 8-9 |
| **Geometry of the network** | **S1** | Describe methods used to explore the geometry of the treatment network under study and potential biases related to it. This should include how the evidence base has been graphically summarized for presentation, and what characteristics were compiled and used to describe the evidence base to readers. | 9 |
| Risk of bias within individual studies | 12 | Describe methods used for assessing risk of bias of individual studies (including specification of whether this was done at the study or outcome level), and how this information is to be used in any data synthesis. | 8 |
| Summary measures | 13 | State the principal summary measures (e.g., risk ratio, difference in means). *Also describe the use of additional summary measures assessed, such as treatment rankings and surface under the cumulative ranking curve (SUCRA) values, as well as modified approaches used to present summary findings from meta-analyses.* | 9 |
| Planned methods of analysis | 14 | Describe the methods of handling data and combining results of studies for each network meta-analysis. This should include, but not be limited to:   - *Handling of multi-arm trials;* - *Selection of variance structure;* - *Selection of prior distributions in Bayesian analyses; and* - *Assessment of model fit.* | 9-10 |
| **Assessment of Inconsistency** | **S2** | Describe the statistical methods used to evaluate the agreement of direct and indirect evidence in the treatment network(s) studied. Describe efforts taken to address its presence when found. | Not applicable |
| Risk of bias across studies | 15 | Specify any assessment of risk of bias that may affect the cumulative evidence (e.g., publication bias, selective reporting within studies). | 9 |
| Additional analyses | 16 | Describe methods of additional analyses if done, indicating which were pre-specified. This may include, but not be limited to, the following:   - Sensitivity or subgroup analyses; - Meta-regression analyses; - *Alternative formulations of the treatment network; and* - *Use of alternative prior distributions for Bayesian analyses (if applicable).* | 8-9 |
| **RESULTS†** |  |  |  |
| Study selection | 17 | Give numbers of studies screened, assessed for eligibility, and included in the review, with reasons for exclusions at each stage, ideally with a flow diagram. | 11, Supplement |
| **Presentation of network structure** | **S3** | Provide a network graph of the included studies to enable visualization of the geometry of the treatment network. | Supplement |
| **Summary of network geometry** | **S4** | Provide a brief overview of characteristics of the treatment network. This may include commentary on the abundance of trials and randomized patients for the different interventions and pairwise comparisons in the network, gaps of evidence in the treatment network, and potential biases reflected by the network structure. | 12-15 |
| Study characteristics | 18 | For each study, present characteristics for which data were extracted (e.g., study size, PICOS, follow-up period) and provide the citations. | 11-12 |
| Risk of bias within studies | 19 | Present data on risk of bias of each study and, if available, any outcome level assessment. | 12, Supplement |
| Results of individual studies | 20 | For all outcomes considered (benefits or harms), present, for each study: 1) simple summary data for each intervention group, and 2) effect estimates and confidence intervals. *Modified approaches may be needed to deal with information from larger networks.* | 12-16, Figures 1-2, Supplement |
| Synthesis of results | 21 | Present results of each meta-analysis done, including confidence/credible intervals. *In larger networks, authors may focus on comparisons versus a particular comparator (e.g. placebo or standard care), with full findings presented in an appendix. League tables and forest plots may be considered to summarize pairwise comparisons.* If additional summary measures were explored (such as treatment rankings), these should also be presented. | 12-16, Figures 1-2, Supplement |
| **Exploration for inconsistency** | **S5** | Describe results from investigations of inconsistency. This may include such information as measures of model fit to compare consistency and inconsistency models, *P* values from statistical tests, or summary of inconsistency estimates from different parts of the treatment network. | Not applicable |
| Risk of bias across studies | 22 | Present results of any assessment of risk of bias across studies for the evidence base being studied. | Table 2, Supplement |
| Results of additional analyses | 23 | Give results of additional analyses, if done (e.g., sensitivity or subgroup analyses, meta-regression analyses*, alternative network geometries studied, alternative choice of prior distributions for Bayesian analyses,* and so forth). | Supplement |
| **DISCUSSION** |  |  |  |
| Summary of evidence | 24 | Summarize the main findings, including the strength of evidence for each main outcome; consider their relevance to key groups (e.g., healthcare providers, users, and policy-makers). | 17-19 |
| Limitations | 25 | Discuss limitations at study and outcome level (e.g., risk of bias), and at review level (e.g., incomplete retrieval of identified research, reporting bias). *Comment on the validity of the assumptions, such as transitivity and consistency. Comment on any concerns regarding network geometry (e.g., avoidance of certain comparisons).* | 19 |
| Conclusions | 26 | Provide a general interpretation of the results in the context of other evidence, and implications for future research. | 20 |
| **FUNDING** |  |  |  |
| Funding | 27 | Describe sources of funding for the systematic review and other support (e.g., supply of data); role of funders for the systematic review. This should also include information regarding whether funding has been received from manufacturers of treatments in the network and/or whether some of the authors are content experts with professional conflicts of interest that could affect use of treatments in the network. | 21 |

Abbreviations: PICOS = population, intervention, comparators, outcomes, study design.

# **Supplementary Methods 2: Search strategy**

**6.10.24 PubMed (397)** (((((((("Antineoplastic Combined Chemotherapy Protocols"[Mesh]) OR ("neoadjuvant chemotherap*" AND "neoadjuvant chemoradiation")) OR ("neoadjuvant chemotherap*")) OR ("neoadjuvant chemoradiation")) OR ("Chemoradiotherapy"[Mesh])) OR ("Neoadjuvant Therapy"[Mesh])) OR ("Chemotherapy, Adjuvant"[Mesh])) AND ((( "Esophagogastric Junction/drug effects"[Mesh] OR "Esophagogastric Junction/pathology"[Mesh] OR "Esophagogastric Junction/radiation effects"[Mesh] OR "Esophagogastric Junction/surgery"[Mesh] )) OR (("Esophageal Neoplasms"[Mesh]) OR ("gastroesophageal junction cancer*" OR "gastroesophageal junction carcinoma*"))) AND (clinicaltrialphaseiii[Filter])) OR ((((((((("Antineoplastic Combined Chemotherapy Protocols"[Mesh]) OR ("neoadjuvant chemotherap*" AND "neoadjuvant chemoradiation")) OR ("neoadjuvant chemotherap*")) OR ("neoadjuvant chemoradiation")) OR ("Chemoradiotherapy"[Mesh])) OR ("Neoadjuvant Therapy"[Mesh])) OR ("Chemotherapy, Adjuvant"[Mesh])) AND ((( "Esophagogastric Junction/drug effects"[Mesh] OR "Esophagogastric Junction/pathology"[Mesh] OR "Esophagogastric Junction/radiation effects"[Mesh] OR "Esophagogastric Junction/surgery"[Mesh] )) OR (("Esophageal Neoplasms"[Mesh]) OR ("gastroesophageal junction cancer*" OR "gastroesophageal junction carcinoma*")))) AND ("phase 2/3" OR "phase III" OR "phase 3")) Filters: English

**6.10.24 Embase <1974 to 2024 June 07>**

1 (Antineoplastic adj1 Combined adj1 Chemotherapy adj1 Protocol*).mp. [mp=title, abstract, heading word, drug trade name, original title, device manufacturer, drug manufacturer, device trade name, keyword heading word, floating subheading word, candidate term word] 582

2 exp neoadjuvant chemotherapy/ 34832

3 (neoadjuvant adj1 chemoradiation).mp. [mp=title, abstract, heading word, drug trade name, original title, device manufacturer, drug manufacturer, device trade name, keyword heading word, floating subheading word, candidate term word] 4649

4 2 and 3 682

5 exp chemoradiotherapy/ 80450

6 exp neoadjuvant therapy/ 58818

7 exp adjuvant chemotherapy/ 75012

8 exp gastroesophageal junction/ 9179

9 (Esophagogastric adj1 Junction).mp. [mp=title, abstract, heading word, drug trade name, original title, device manufacturer, drug manufacturer, device trade name, keyword heading word, floating subheading word, candidate term word] 5373

10 exp esophagus tumor/ 109609

11 (Esophageal adj1 Neoplasm*).mp. [mp=title, abstract, heading word, drug trade name, original title, device manufacturer, drug manufacturer, device trade name, keyword heading word, floating subheading word, candidate term word] 4025

12 (Esophageal adj1 cancer*).mp. [mp=title, abstract, heading word, drug trade name, original title, device manufacturer, drug manufacturer, device trade name, keyword heading word, floating subheading word, candidate term word] 41565

13 (gastroesophageal adj1 junction adj1 carcinoma*).mp. [mp=title, abstract, heading word, drug trade name, original title, device manufacturer, drug manufacturer, device trade name, keyword heading word, floating subheading word, candidate term word] 202

14 (gastroesophageal adj1 junction adj1 cancer*).mp. [mp=title, abstract, heading word, drug trade name, original title, device manufacturer, drug manufacturer, device trade name, keyword heading word, floating subheading word, candidate term word] 1171

15 8 or 9 or 13 or 14 12713

16 1 or 4 or 5 or 6 or 7 187682

17 15 and 16 1843

18 (surger* or surgic*).mp. [mp=title, abstract, heading word, drug trade name, original title, device manufacturer, drug manufacturer, device trade name, keyword heading word, floating subheading word, candidate term word] 5062080

19 17 and 18 1545

20 limit 19 to (clinical trial or randomized controlled trial or phase 3 clinical trial) 242

21 limit 20 to english language 238

<https://ovidsp.ovid.com/ovidweb.cgi?T=JS&NEWS=N&PAGE=main&SHAREDSEARCHID=4J7cDdKU5oJEDhsnoOsikczmeLQF20Vobs8BWTVIf7sJAmuBPJJw3TGVuhffovsCh>

**6.10.24 Scopus (346**) ( TITLE-ABS-KEY ( "phase iii" OR "phase 3" OR "phase three" ) ) AND ( ( ( TITLE-ABS-KEY ( "esophagogastric junction" ) ) OR ( TITLE-ABS-KEY ( "esophageal neoplasm*" OR "esophageal cancer*" ) ) OR ( TITLE-ABS-KEY ( "gastroesophageal junction cancer*" OR "gastroesophageal junction carcinoma*" ) ) ) AND ( ( TITLE-ABS-KEY ( "adjuvant chemotherap*" ) ) OR ( TITLE-ABS-KEY ( "neoadjuvant therapy" ) ) OR ( TITLE-ABS-KEY ( chemotherapy ) ) OR ( TITLE-ABS-KEY ( "neoadjuvant chemoradiation*" ) ) OR ( TITLE-ABS-KEY ( "neoadjuvant chemotherap*" ) ) AND ( ( TITLE-ABS-KEY ( "neoadjuvant chemotherap*" ) AND TITLE-ABS-KEY ( "neoadjuvant chemoradiation" ) ) ) OR ( TITLE-ABS-KEY ( "antineoplastic combined chemotherapy protocol*" ) ) ) ) AND ( LIMIT-TO ( LANGUAGE , "english" ) )

**6.10.24 Web of Science (302)**

<https://www.webofscience.com/wos/woscc/summary/64bd9ca2-c9df-4e4b-acde-c6ab7182629a-f2b4a6ce/relevance/1> "esophagogastric junction" OR "esophageal neoplasm*" OR "esophageal cancer*" (Topic) OR "gastroesophageal junction cancer*" OR "gastroesophageal junction carcinoma*" (Topic)

**AND**

"adjuvant chemotherap*" OR "neoadjuvant therapy" (Topic) OR "antineoplastic combined chemotherapy protocol*" (Topic) OR "neoadjuvant chemotherap*" AND "neoadjuvant chemoradiation" (Topic) or "neoadjuvant chemotherap*" OR "neoadjuvant chemoradiation" (Topic)

**AND**

"phase iii" OR "phase 3" OR "phase three" (All Fields) and English (Languages)

**6.10.24 EBM Reviews - Cochrane Central Register of Controlled Trials <May 2024>**

1 exp Esophagogastric Junction/ 711

2 (Esophageal adj1 Neoplasm*).mp. 2575

3 (gastroesophageal adj1 junction adj1 cancer*).mp. 274

4 (gastroesophageal adj1 junction adj1 carcinoma*).mp. 21

5 1 or 2 or 3 or 4 3274

6 (Antineoplastic adj1 Combined adj1 Chemotherapy adj1 Protocol*).ti,ab. 2

7 (neoadjuvant adj1 chemotherap*).mp. 4972

8 (neoadjuvant adj1 chemoradiation).mp 274

9 exp Chemoradiotherapy, Adjuvant/ or exp Chemoradiotherapy/ 2094

10 7 and 8 36

11 6 or 7 or 8 or 9 or 10 7136

12 5 and 11 375

13 (phase adj1 III).mp. 48976

14 12 and 13 72

15 limit 12 to english language 351

| **DATABASE** | **RESULTS** | **DUPLICATES** | **REMAINING** |
| --- | --- | --- | --- |
| PubMed | 397 | 6 | 391 |
| Embase | 238 | 24 | 214 |
| Scopus | 346 | 291 | 55 |
| Web of Science | 302 | 52 | 250 |
| Cochrane Central Register of Controlled Trials | 351 | 126 | 225 |
| Author Supplied | 5 | 0 | 5 |
| **TOTAL** | **1639** | **499** | **1140** |

# **Supplementary Figure 1: PRISMA flow diagram showing the screening and selection process**


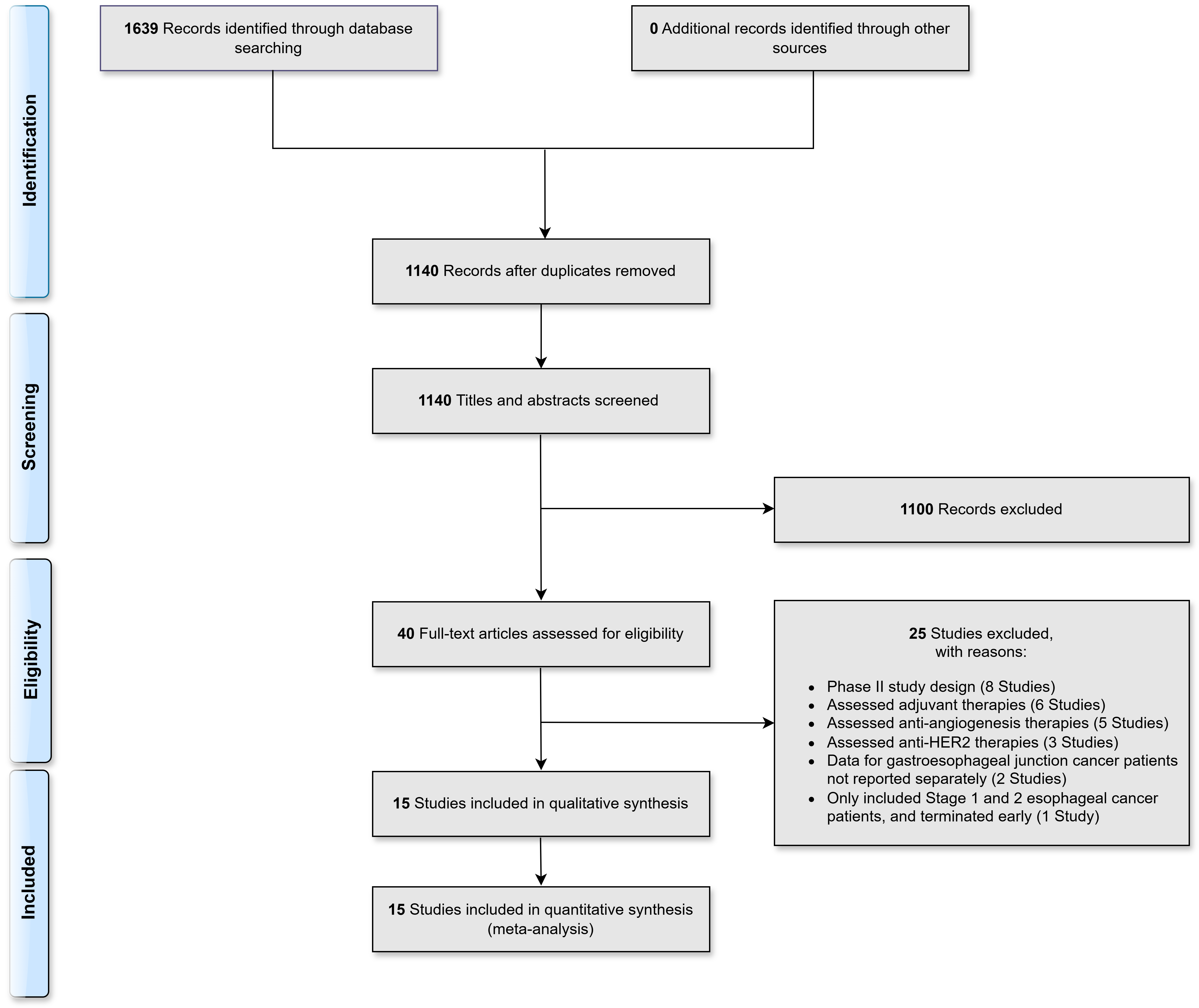


# **Supplementary Figure 2: Risk of bias in the included studies**

1. **Overall survival**

**
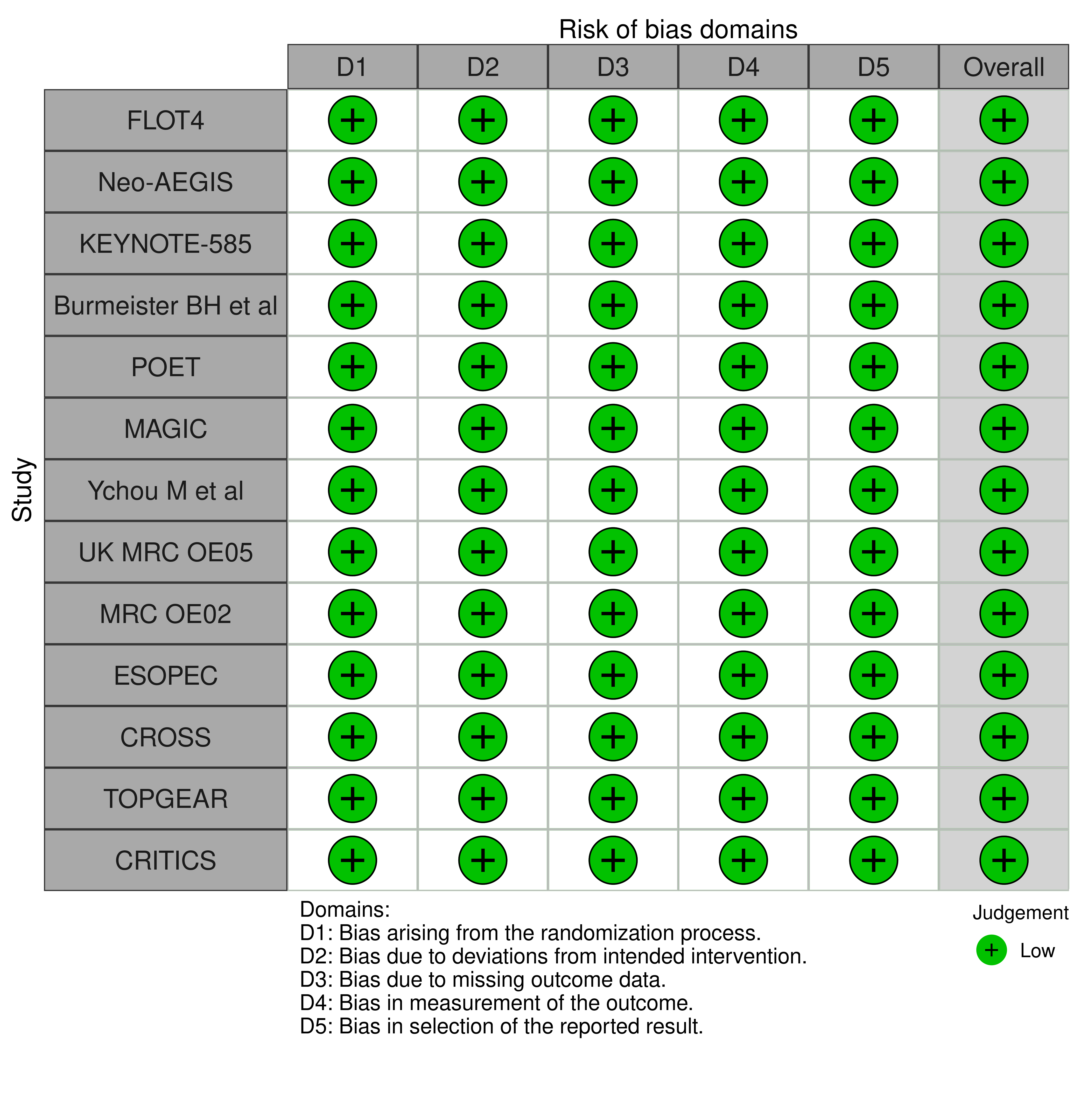
**

1. **Disease-free survival**

**
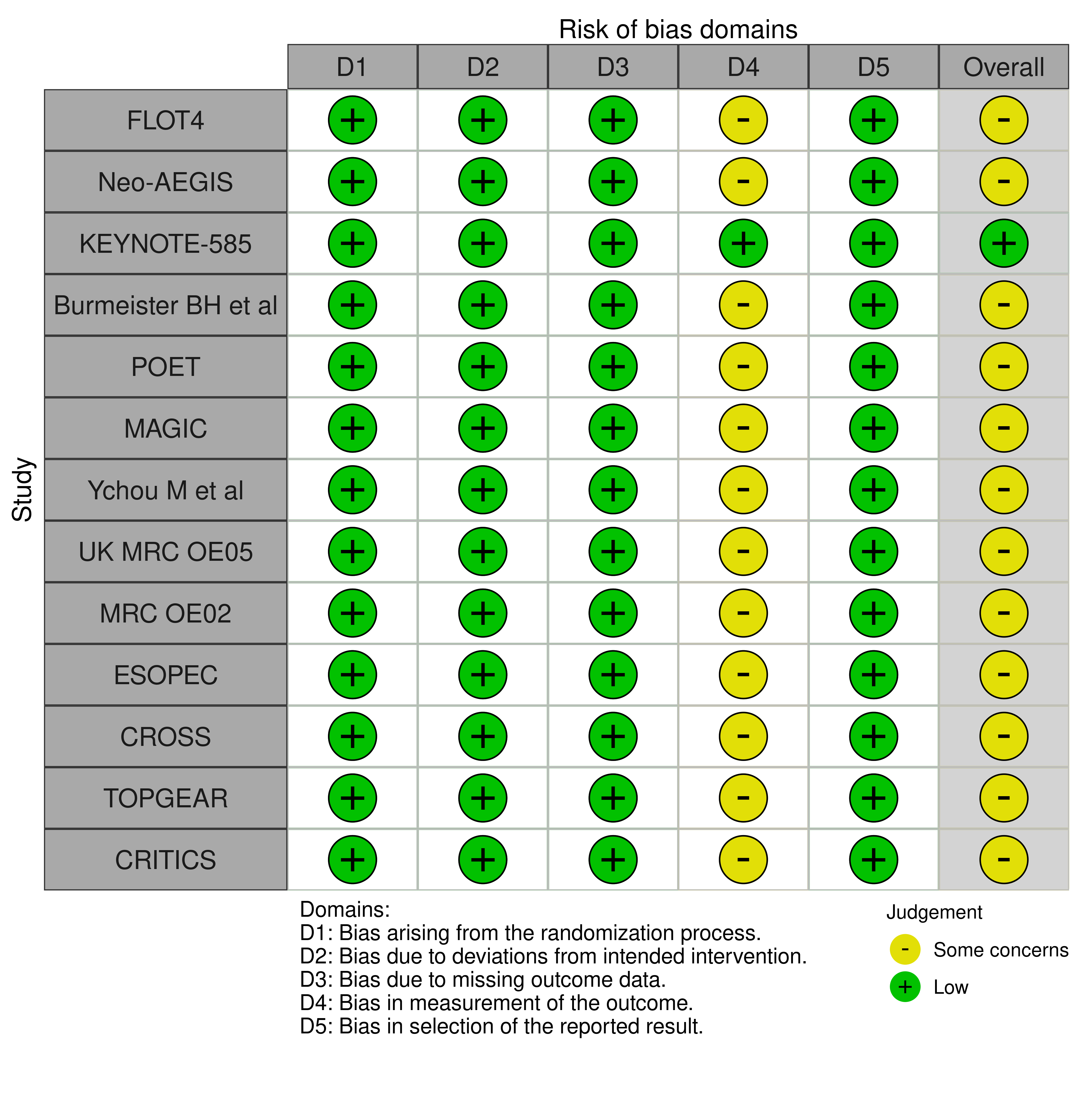
**

1. **Pathologic complete response**


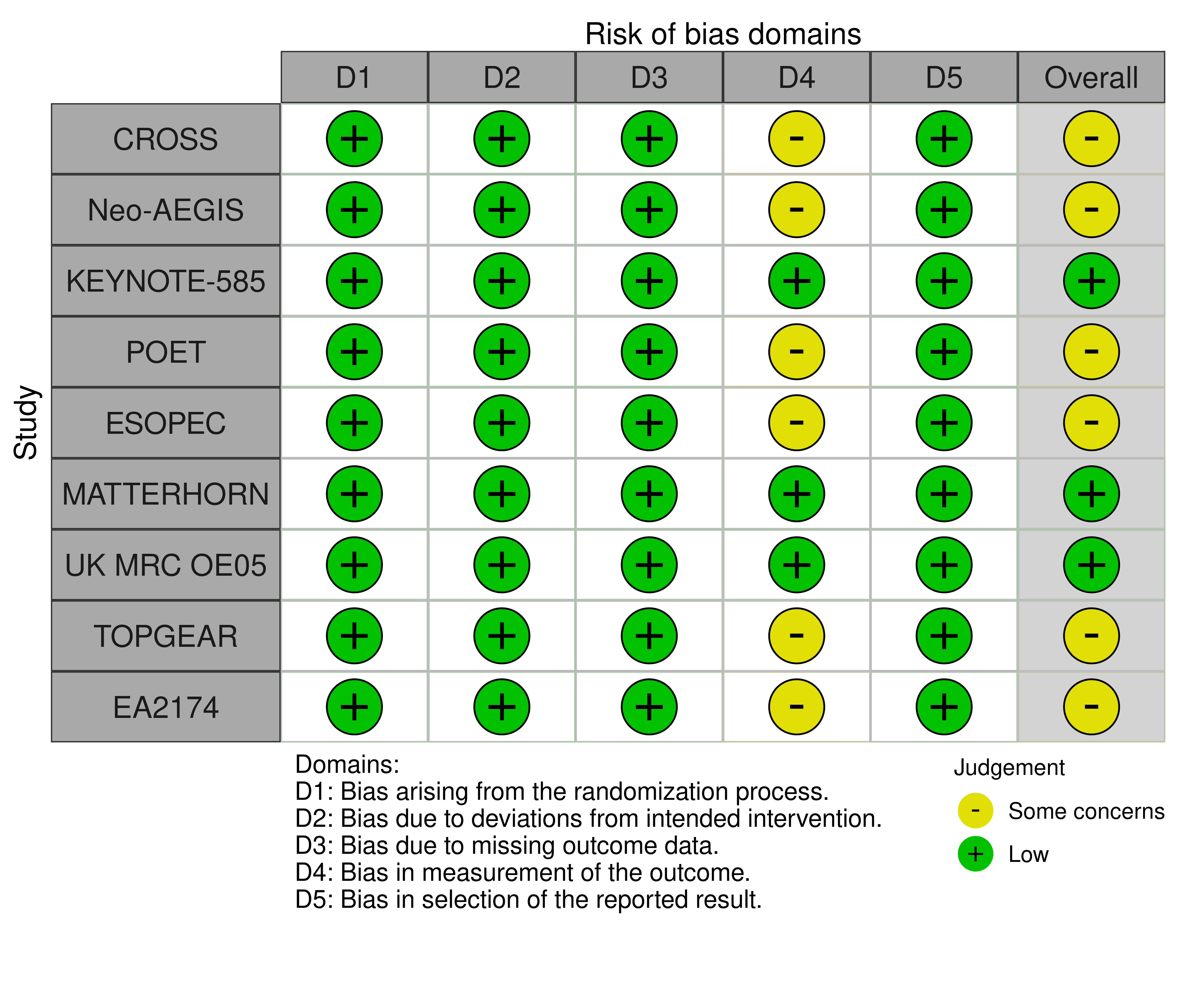


# **Supplementary Figure 3: Network plot for disease-free survival in the overall population**


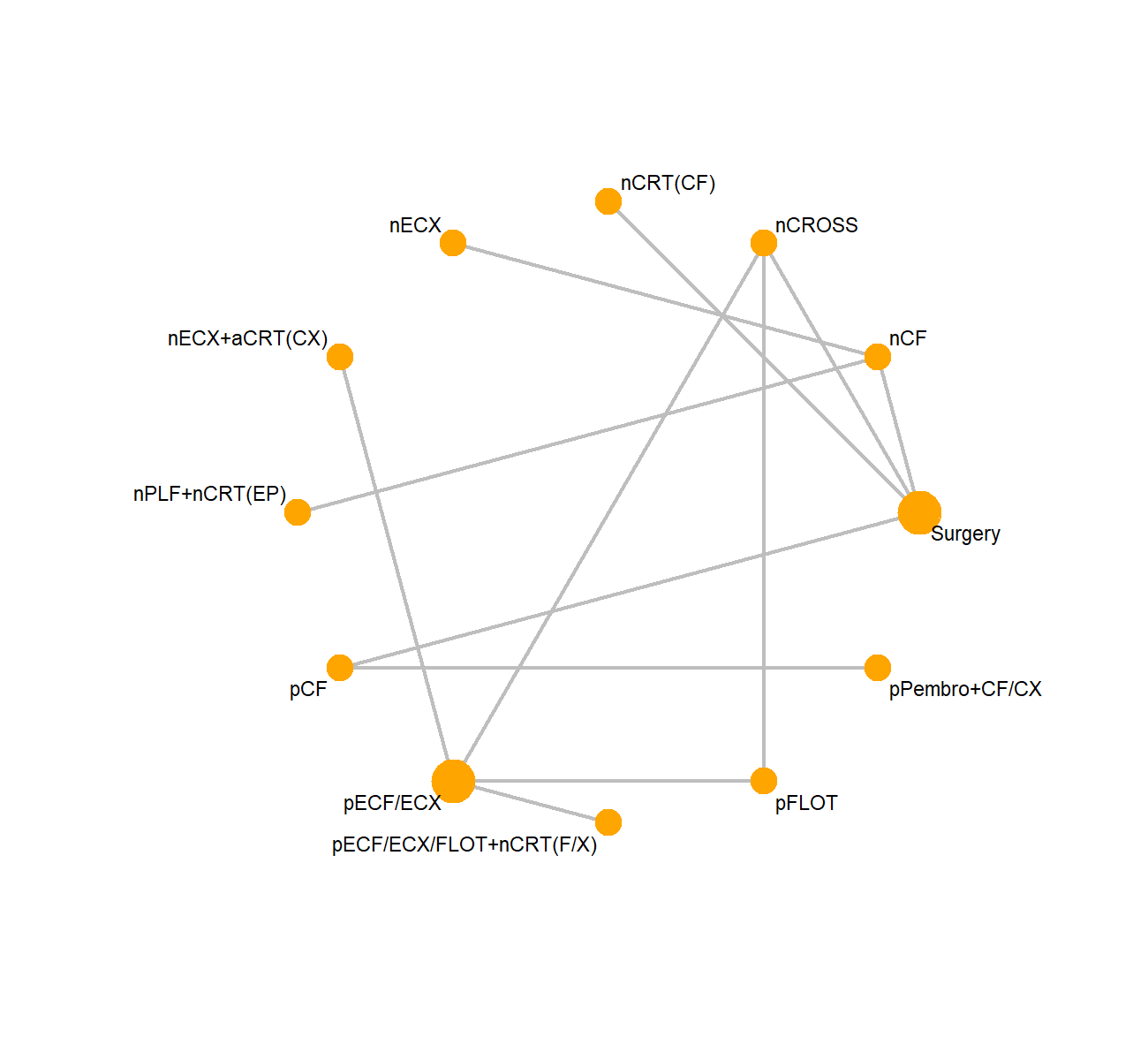


Each node represents a treatment. Each edge (line) represents direct comparison between the treatments/controls. Larger node and thicker edge represent relatively higher number of trials for that comparison.

Abbreviations: nECX: neoadjuvant epirubicin, cisplatin, and capecitabine; nCRT(CF): neoadjuvant cisplatin and fluorouracil with radiotherapy; nCROSS: neoadjuvant paclitaxel and carboplatin with radiotherapy; nCF: neoadjuvant cisplatin and fluorouracil; pPembro+CF/CX: perioperative pembrolizumab with cisplatin and fluorouracil/capecitabine; pFLOT: perioperative fluorouracil, leucovorin, oxaliplatin, and docetaxel; pECF/ECX/FLOT+nCRT(F/X): either perioperative epirubicin with cisplatin and fluorouracil/capecitabine, or perioperative fluorouracil, leucovorin, oxaliplatin, and docetaxel, along with neoadjuvant chemoradiotherapy (fluorouracil/capecitabine and radiotherapy); pECF/ECX: perioperative epirubicin, cisplatin, and fluorouracil/capecitabine; pCF: perioperative cisplatin and fluorouracil; nPLF+nCRT(EP): neoadjuvant cisplatin, leucovorin, and fluorouracil for induction, followed by etoposide and cisplatin with concomitant radiotherapy; nECX+aCRT(CX): neoadjuvant epirubicin, cisplatin/oxaliplatin and capecitabine, followed by adjuvant chemoradiotherapy with radiotherapy, cisplatin and capecitabine

# **Supplementary Figure 4: Network plot for disease-free Survival in the overall population, with data for TOPGEAR trial stratified according to each treatment combination administered**


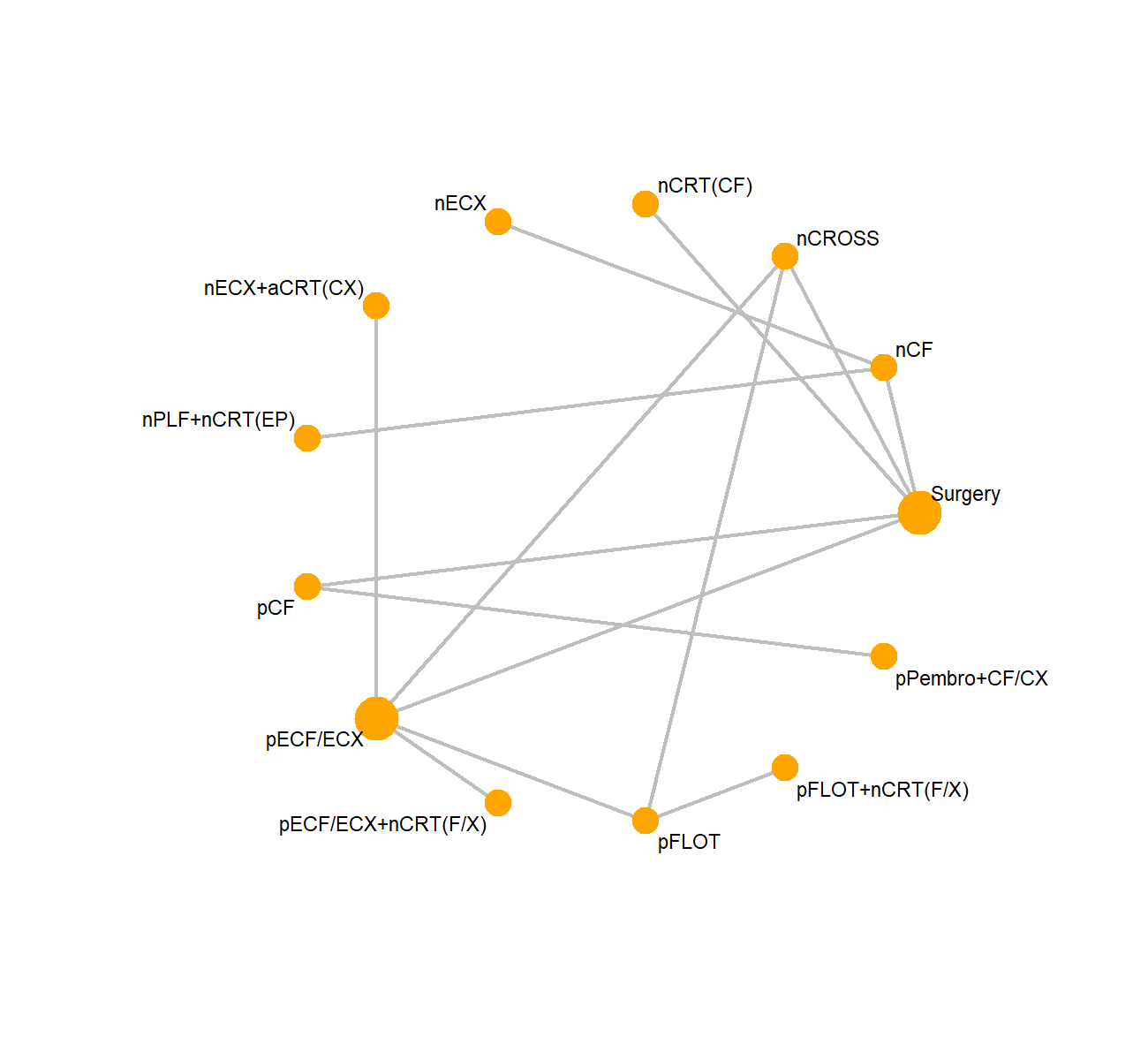


Each node represents a treatment. Each edge (line) represents direct comparison between the treatments/controls. Larger node and thicker edge represent relatively higher number of trials for that comparison.

Abbreviations: nECX: neoadjuvant epirubicin, cisplatin, and capecitabine; nCRT(CF): neoadjuvant cisplatin and fluorouracil with radiotherapy; nCROSS: neoadjuvant paclitaxel and carboplatin with radiotherapy; nCF: neoadjuvant cisplatin and fluorouracil; pPembro+CF/CX: perioperative pembrolizumab with cisplatin and fluorouracil/capecitabine; pFLOT+nCRT(F/X): perioperative fluorouracil, leucovorin, oxaliplatin, and docetaxel, along with neoadjuvant chemoradiotherapy (fluorouracil/capecitabine and radiotherapy); pFLOT: perioperative fluorouracil, leucovorin, oxaliplatin, and docetaxel; pECF/ECX+nCRT(F/X): perioperative epirubicin with cisplatin and fluorouracil/capecitabine, along with neoadjuvant chemoradiotherapy (fluorouracil/capecitabine and radiotherapy); pECF/ECX: perioperative epirubicin, cisplatin, and fluorouracil/capecitabine; pCF: perioperative cisplatin and fluorouracil; nPLF+nCRT(EP): neoadjuvant cisplatin, leucovorin, and fluorouracil for induction, followed by etoposide and cisplatin with concomitant radiotherapy; nECX+aCRT(CX): neoadjuvant epirubicin, cisplatin/oxaliplatin and capecitabine, followed by adjuvant chemoradiotherapy with radiotherapy, cisplatin and capecitabine

# **Supplementary Figure 5: Network plot for disease-free survival in the overall population, excluding the POET trial**


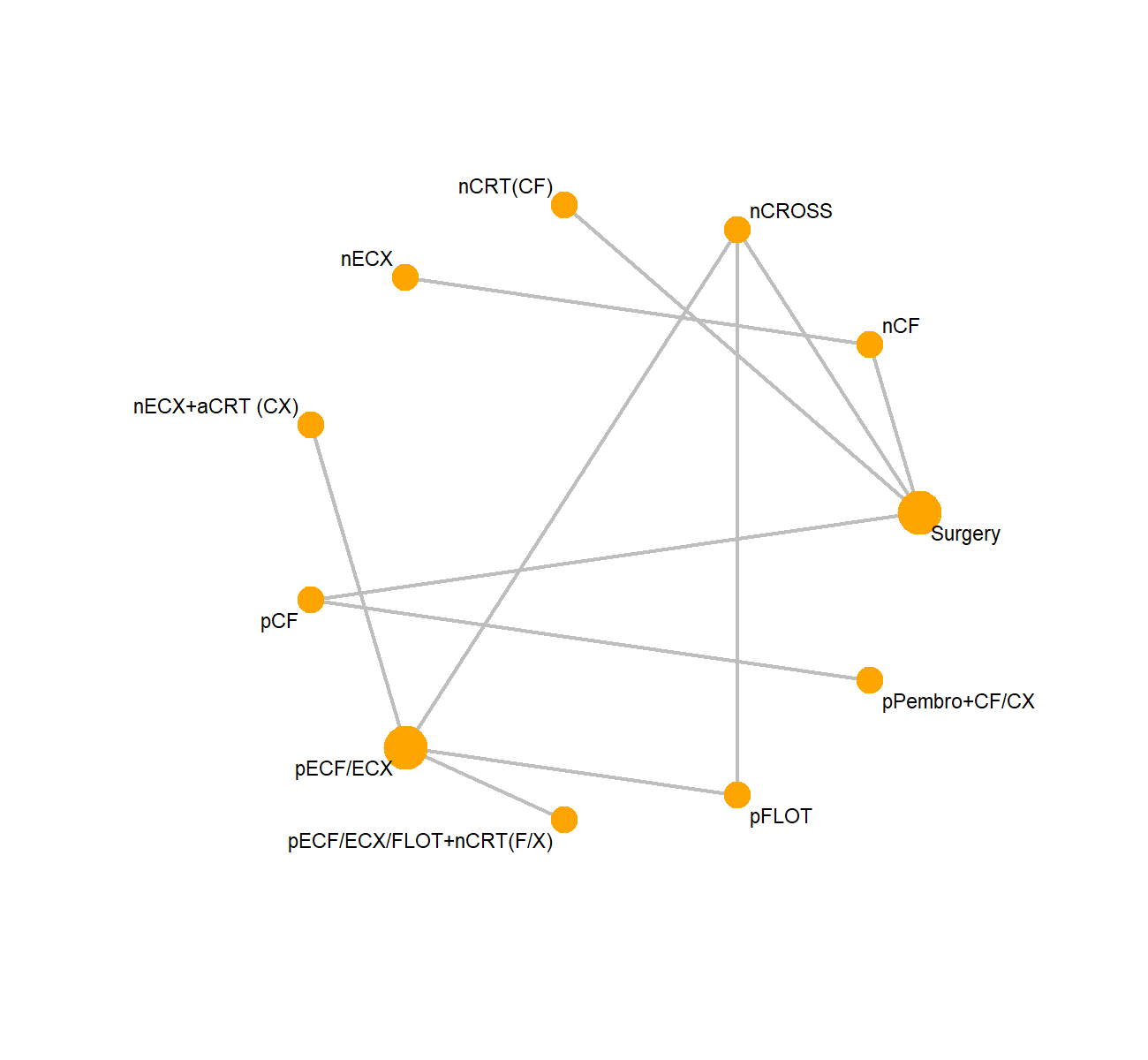


Each node represents a treatment. Each edge (line) represents direct comparison between the treatments/controls. Larger node and thicker edge represent relatively higher number of trials for that comparison.

Abbreviations: nECX: neoadjuvant epirubicin, cisplatin, and capecitabine; nCRT(CF): neoadjuvant cisplatin and fluorouracil with radiotherapy; nCROSS: neoadjuvant paclitaxel and carboplatin with radiotherapy; nCF: neoadjuvant cisplatin and fluorouracil; pPembro+CF/CX: perioperative pembrolizumab with cisplatin and fluorouracil/capecitabine; pFLOT: perioperative fluorouracil, leucovorin, oxaliplatin, and docetaxel; pECF/ECX/FLOT+nCRT(F/X): either perioperative epirubicin with cisplatin and fluorouracil/capecitabine, or perioperative fluorouracil, leucovorin, oxaliplatin, and docetaxel, along with neoadjuvant chemoradiotherapy (fluorouracil/capecitabine and radiotherapy); pECF/ECX: perioperative epirubicin, cisplatin, and fluorouracil/capecitabine; pCF: perioperative cisplatin and fluorouracil; nECX+aCRT(CX): neoadjuvant epirubicin, cisplatin/oxaliplatin and capecitabine, followed by adjuvant chemoradiotherapy with radiotherapy, cisplatin and capecitabine

# **Supplementary Figure 6: Mixed treatment comparisons for disease-free survival in the overall population**

**
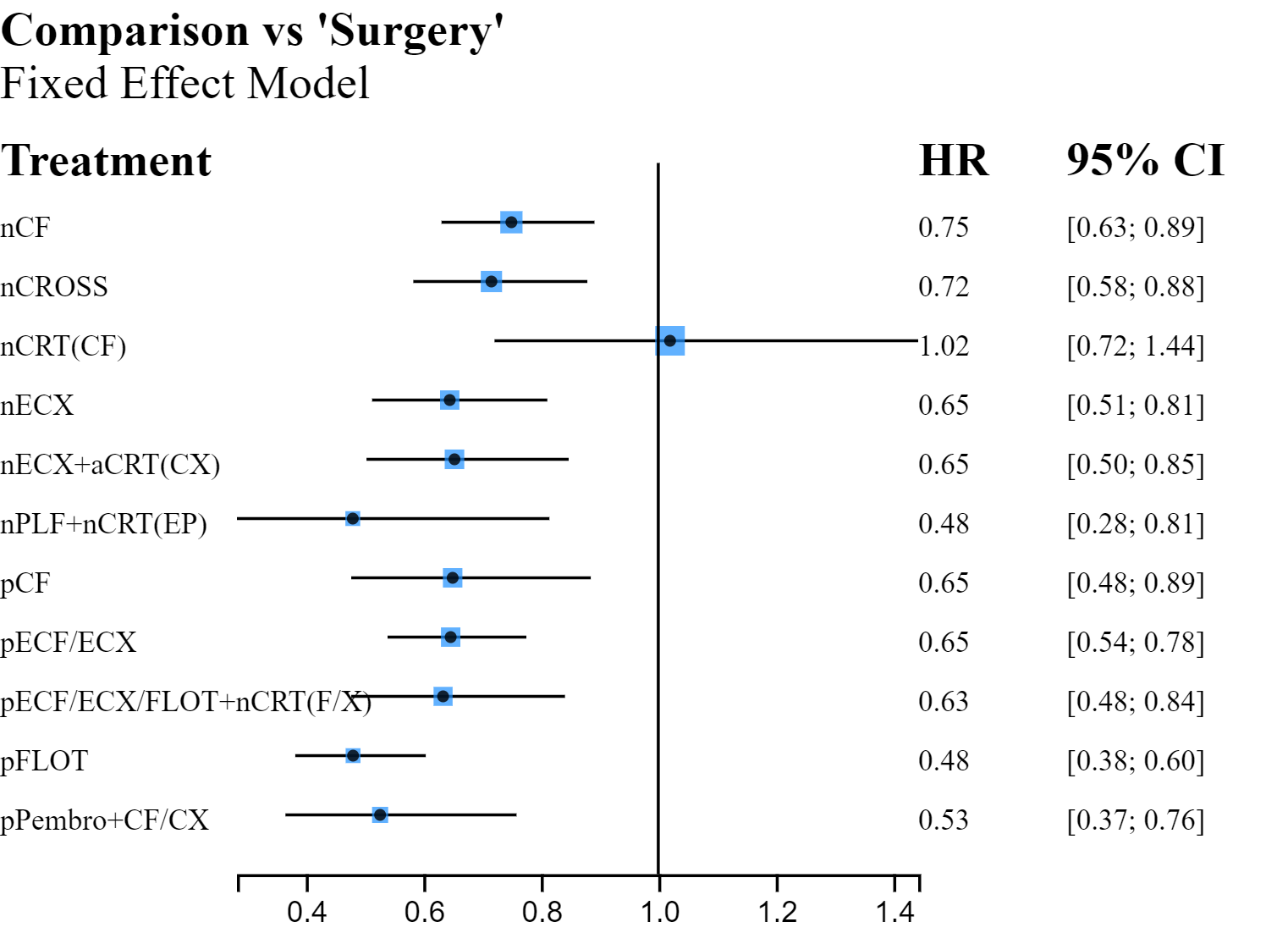
**


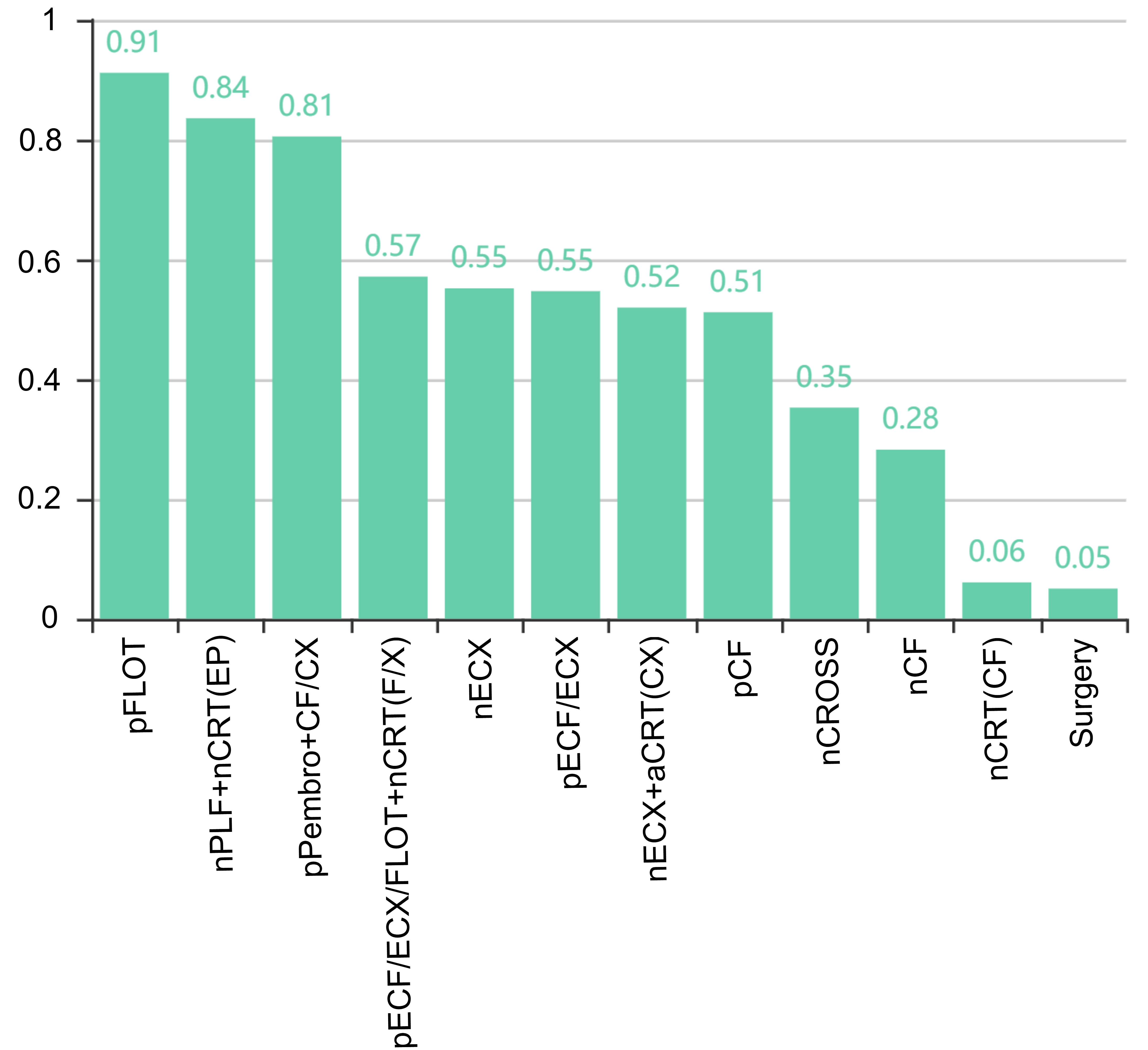


# **Supplementary Figure 7: Mixed treatment comparisons for disease-free survival in the overall population, with data for TOPGEAR trial stratified according to each treatment combination administered**


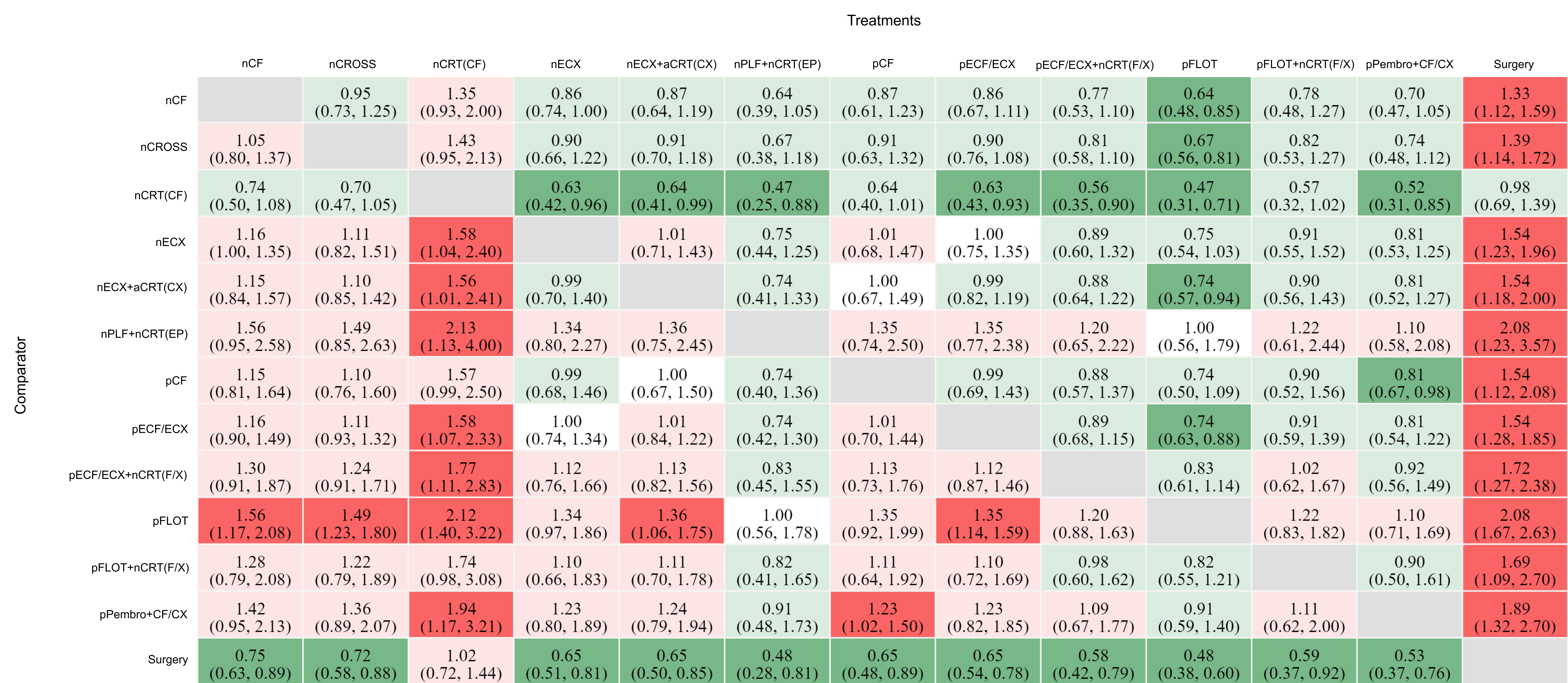


The values in each cell represent the relative treatment effect (and 95% CI) of the treatment on the top, compared to the treatment on the left. Green color suggests relative treatment benefit. Light green suggests non-significant benefit and dark green suggests significant benefit. Red color suggests relative treatment harm. Light red suggests non-significant harm and dark red suggests significant harm.


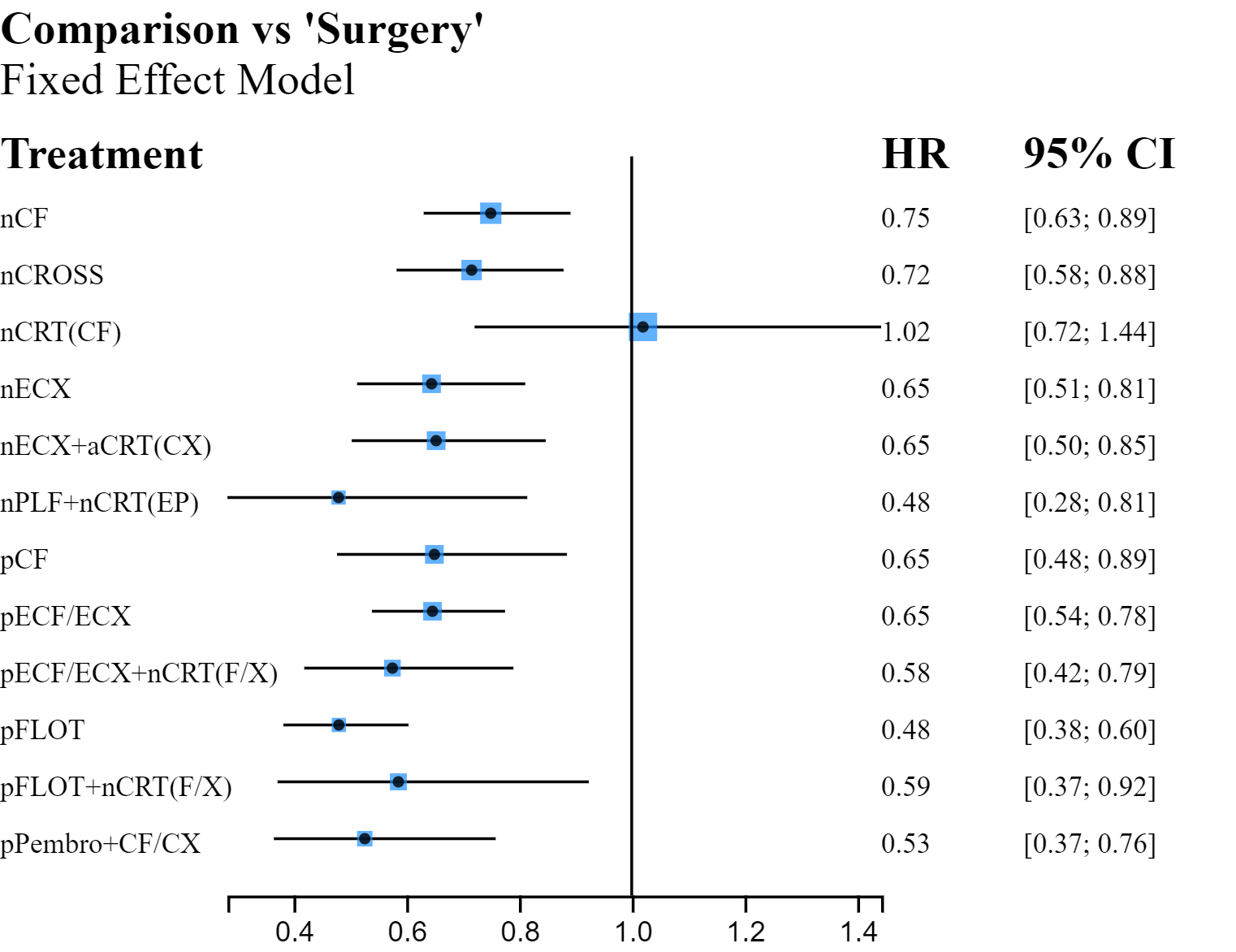


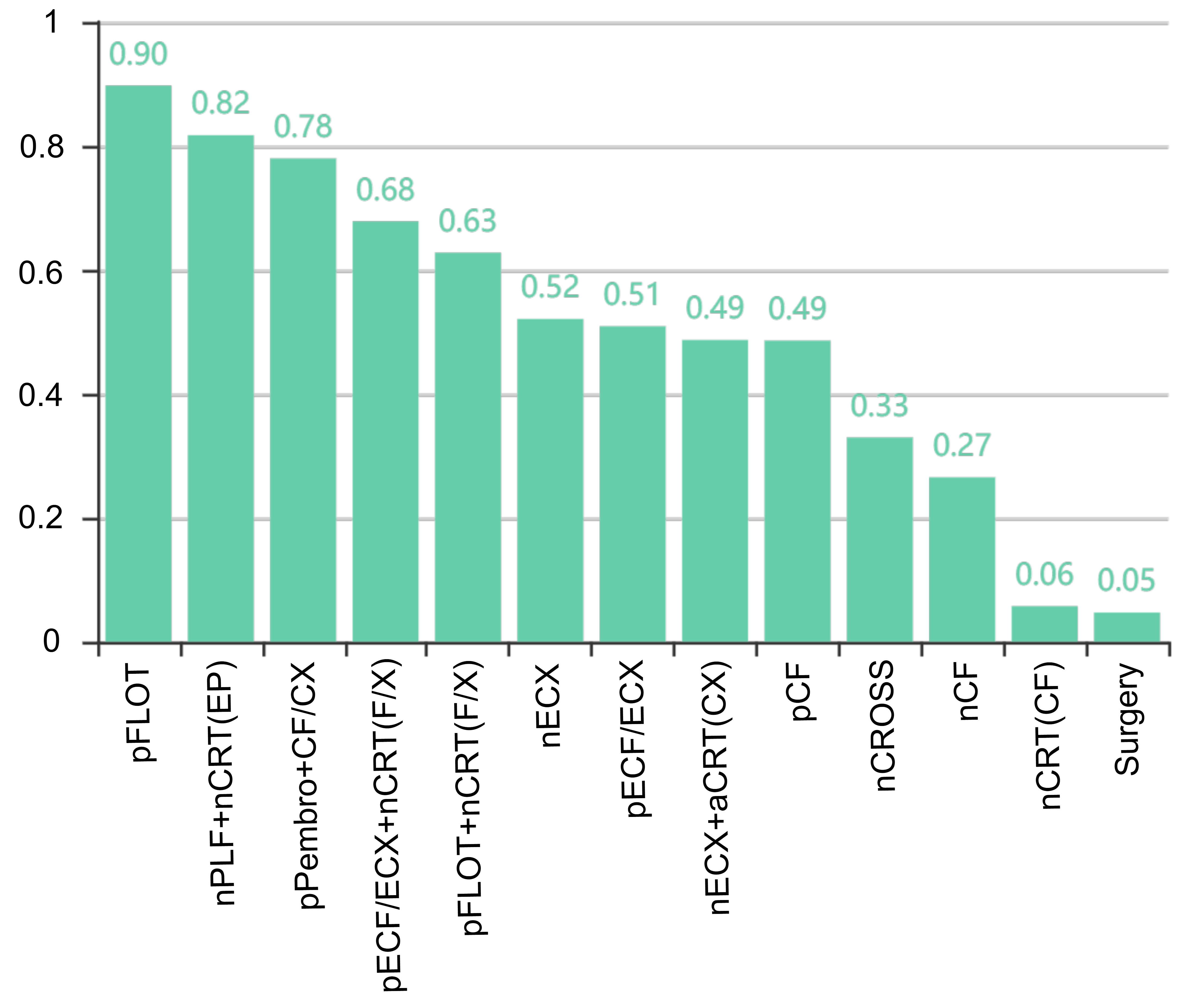


# **Supplementary Figure 8: Mixed treatment comparisons for disease-free survival in the overall population, excluding the POET trial**


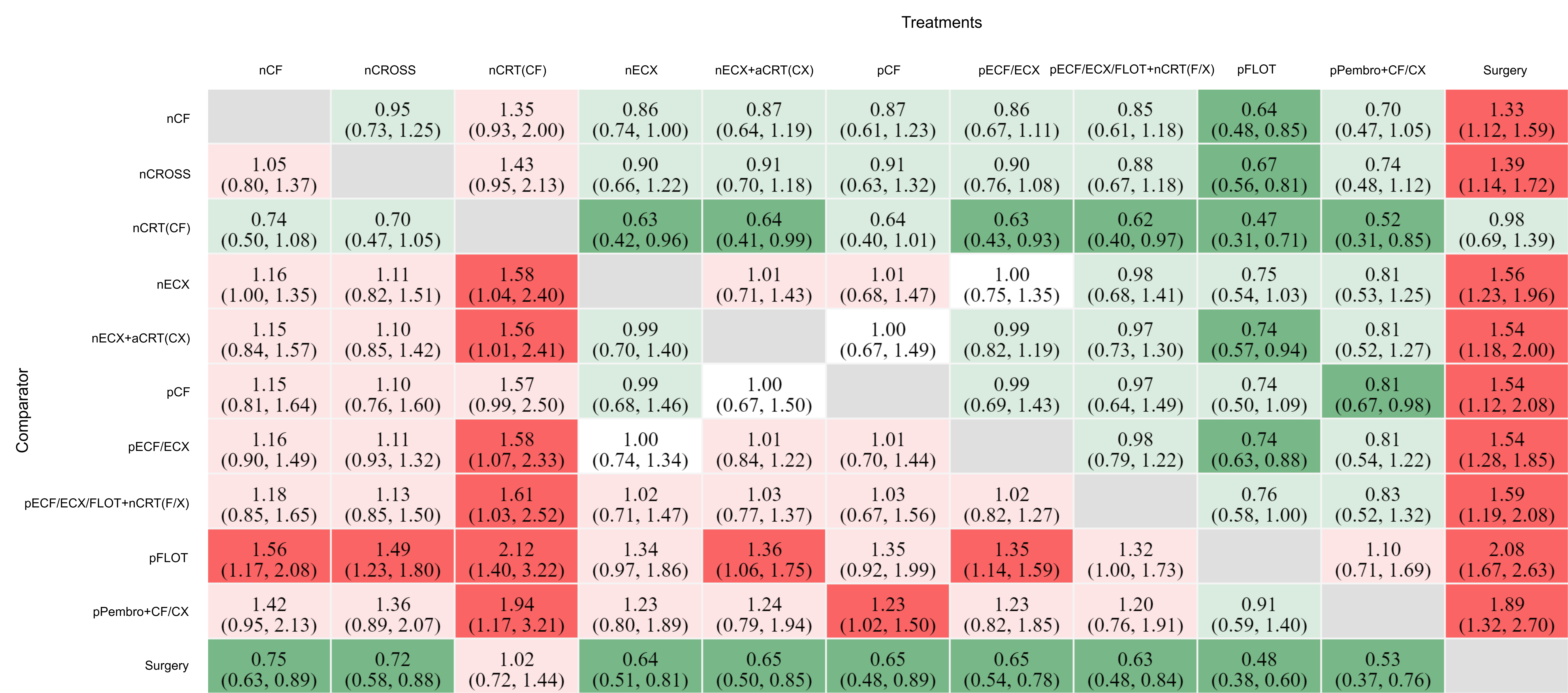


The values in each cell represent the relative treatment effect (and 95% CI) of the treatment on the top, compared to the treatment on the left. Green color suggests relative treatment benefit. Light green suggests non-significant benefit and dark green suggests significant benefit. Red color suggests relative treatment harm. Light red suggests non-significant harm and dark red suggests significant harm.


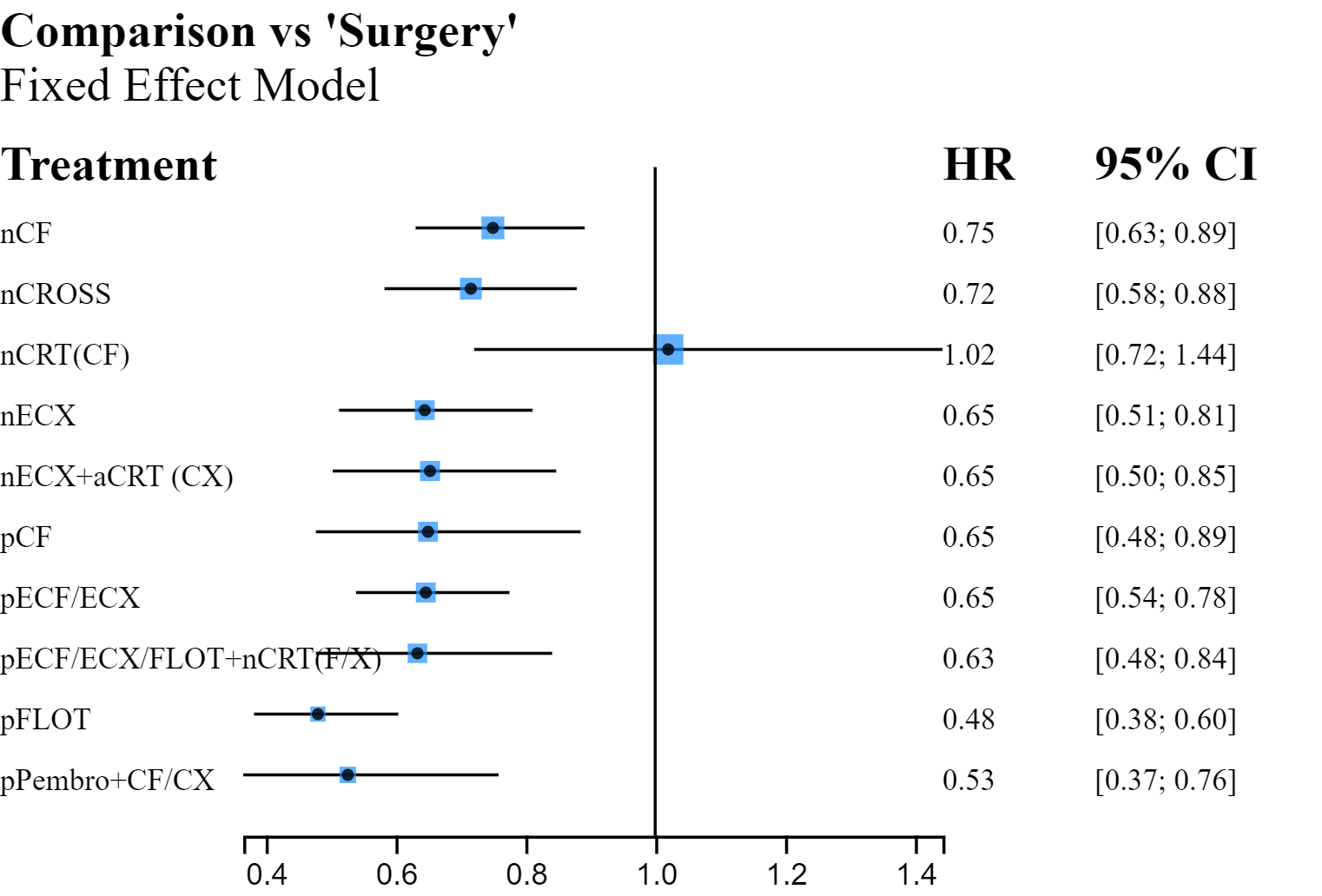


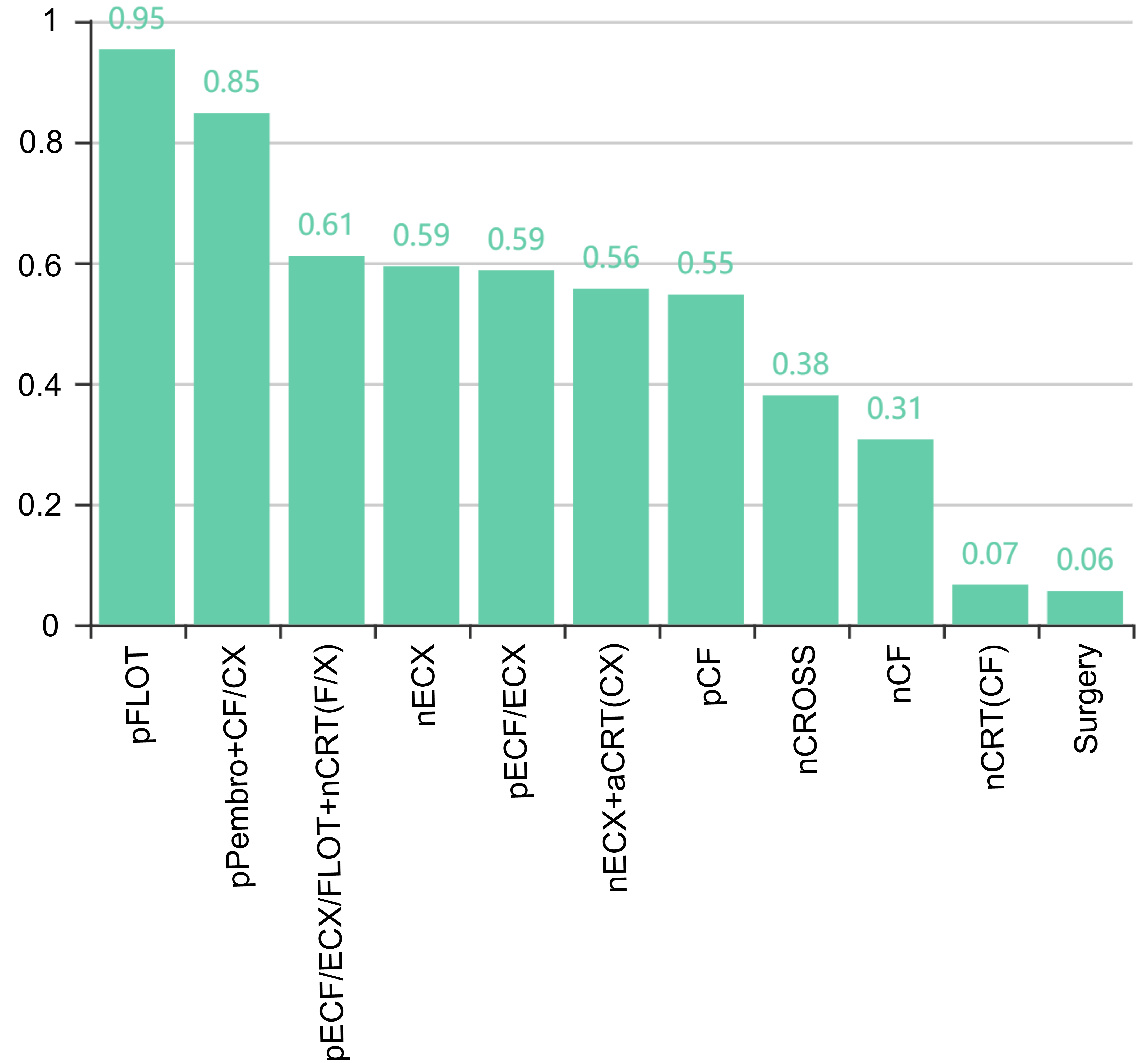


# **Supplementary Figure 9: Network plot for disease-free survival in the GEJ cohort, excluding the POET and KEYNOTE 585 trials**


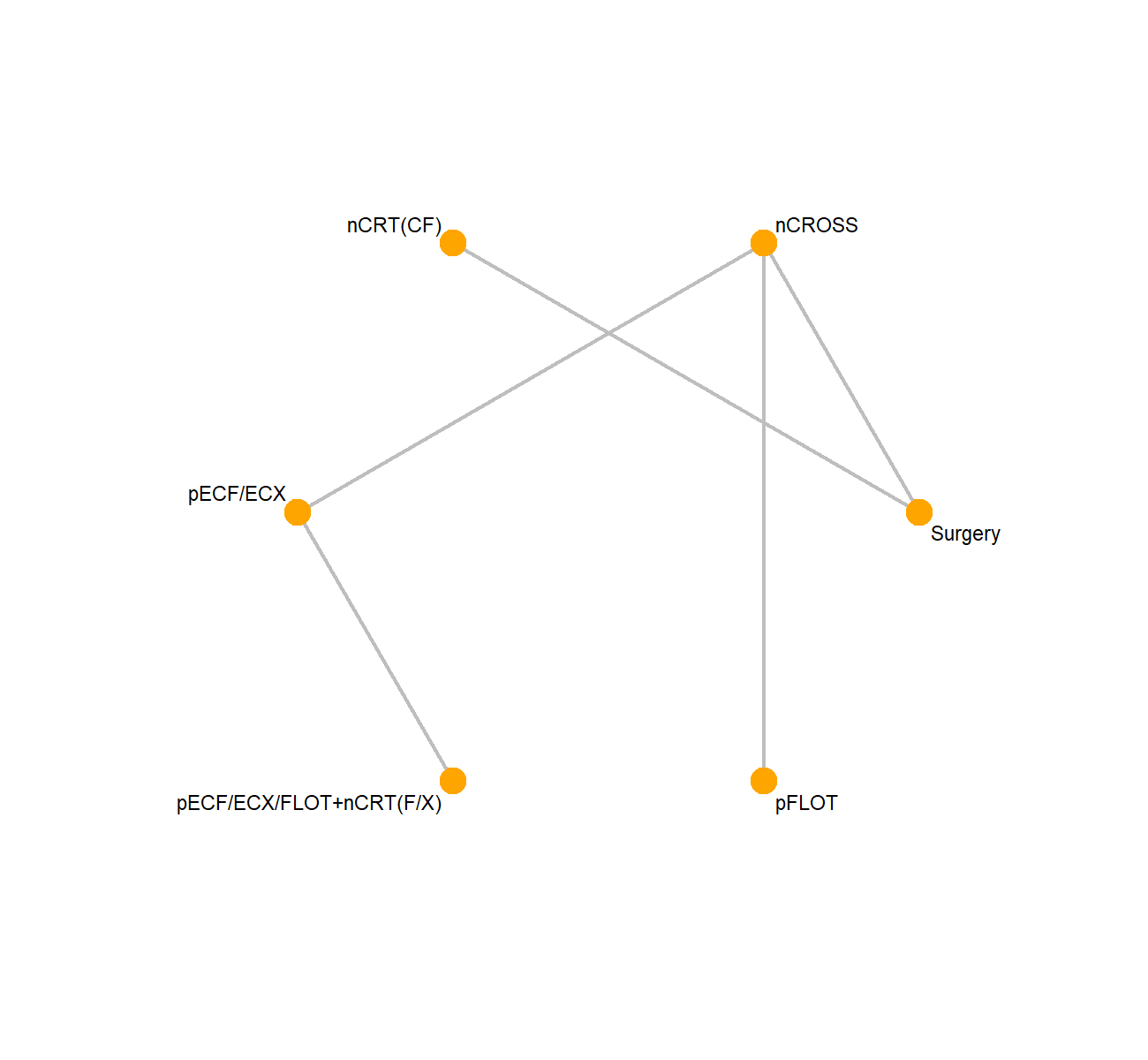


Each node represents a treatment. Each edge (line) represents direct comparison between the treatments/controls. Larger node and thicker edge represent relatively higher number of trials for that comparison.

Abbreviations: nCRT(CF): neoadjuvant cisplatin and fluorouracil with radiotherapy; nCROSS: neoadjuvant paclitaxel and carboplatin with radiotherapy; pFLOT: perioperative fluorouracil, leucovorin, oxaliplatin, and docetaxel; pECF/ECX/FLOT+nCRT(F/X): either perioperative epirubicin with cisplatin and fluorouracil/capecitabine, or perioperative fluorouracil, leucovorin, oxaliplatin, and docetaxel, along with neoadjuvant chemoradiotherapy (fluorouracil/capecitabine and radiotherapy); pECF/ECX: perioperative epirubicin, cisplatin, and fluorouracil/capecitabine

# **Supplementary Figure 10: Mixed treatment comparisons for disease-free survival in the GEJ cohort, excluding the POET and KEYNOTE 585 trials**


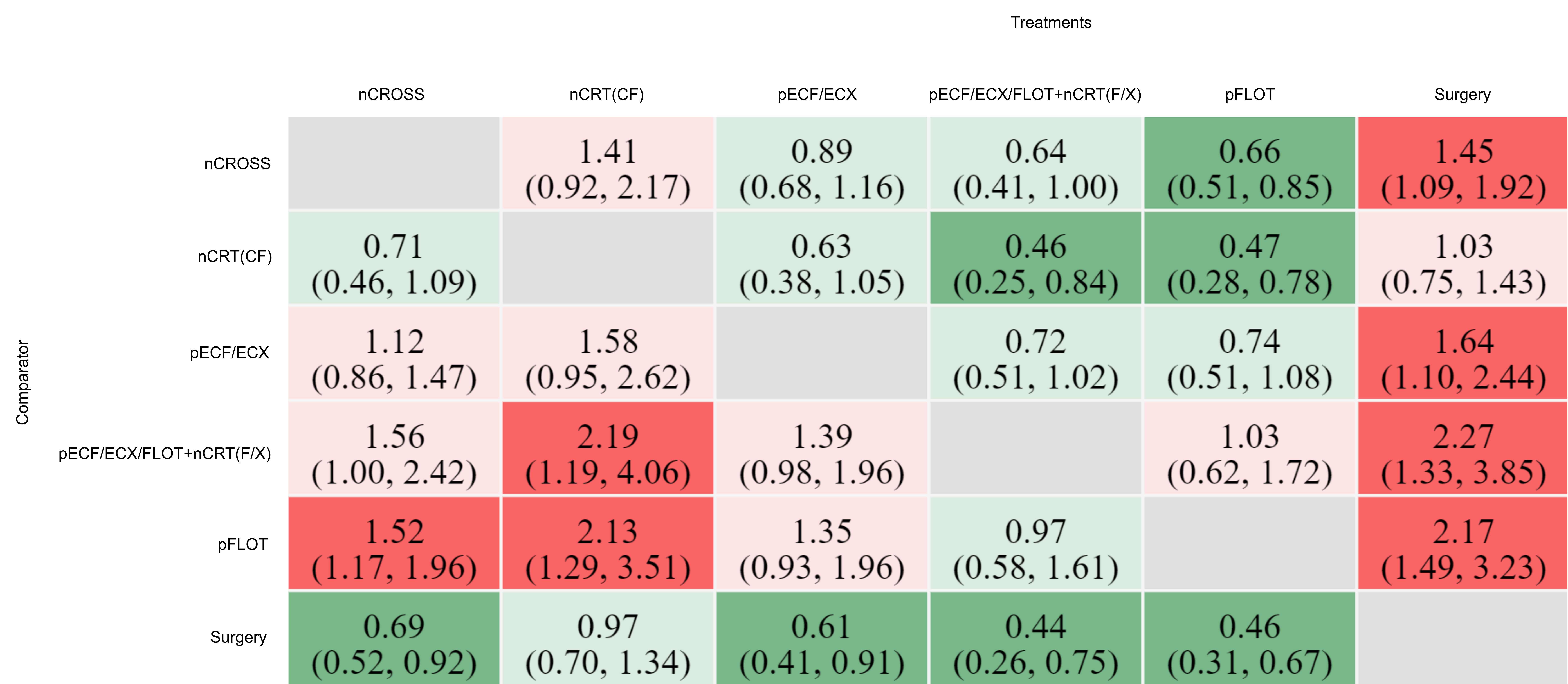


The values in each cell represent the relative treatment effect (and 95% CI) of the treatment on the top, compared to the treatment on the left. Green color suggests relative treatment benefit. Light green suggests non-significant benefit and dark green suggests significant benefit. Red color suggests relative treatment harm. Light red suggests non-significant harm and dark red suggests significant harm.


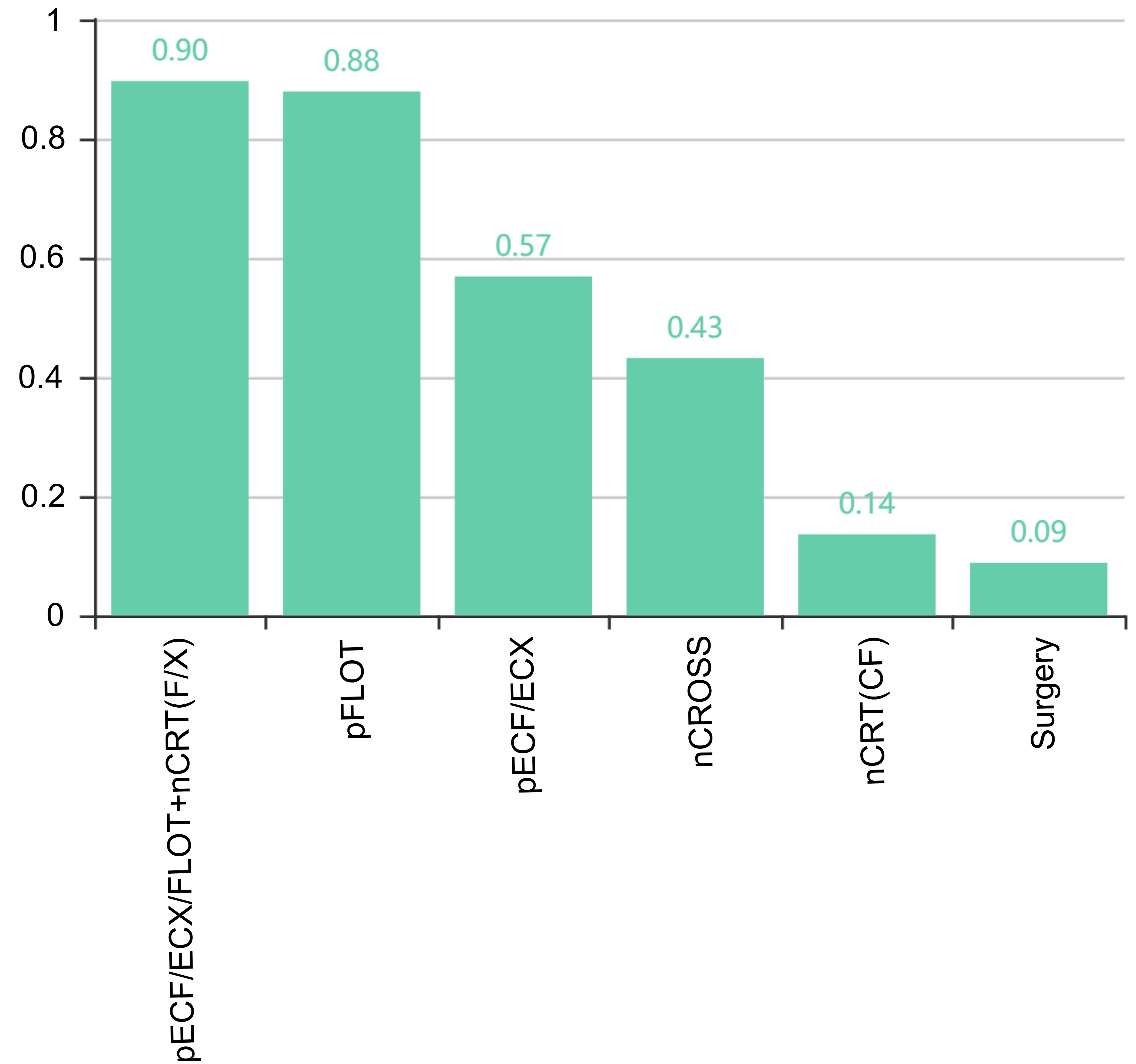


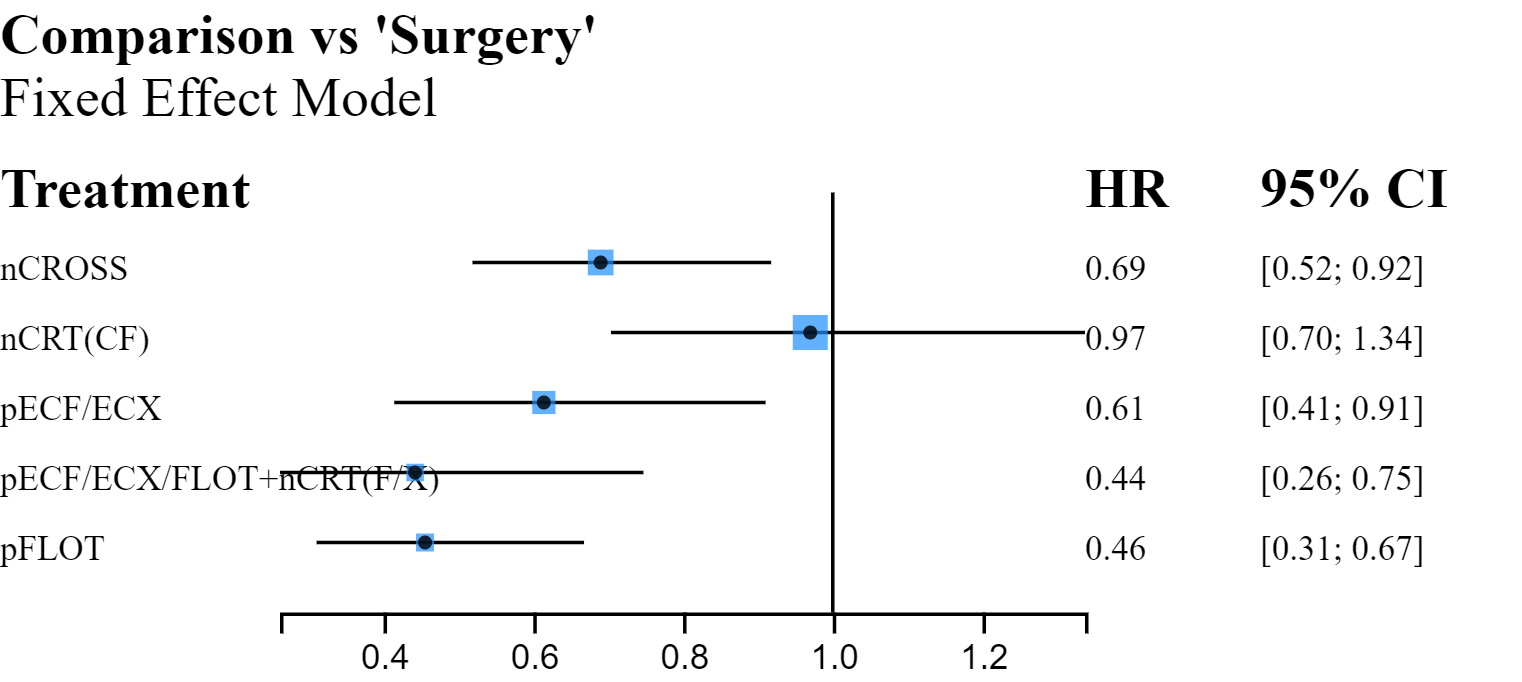


# **Supplementary Figure 11: Network plot for overall survival in the overall population (left) and the GEJ cohort (right)**

**
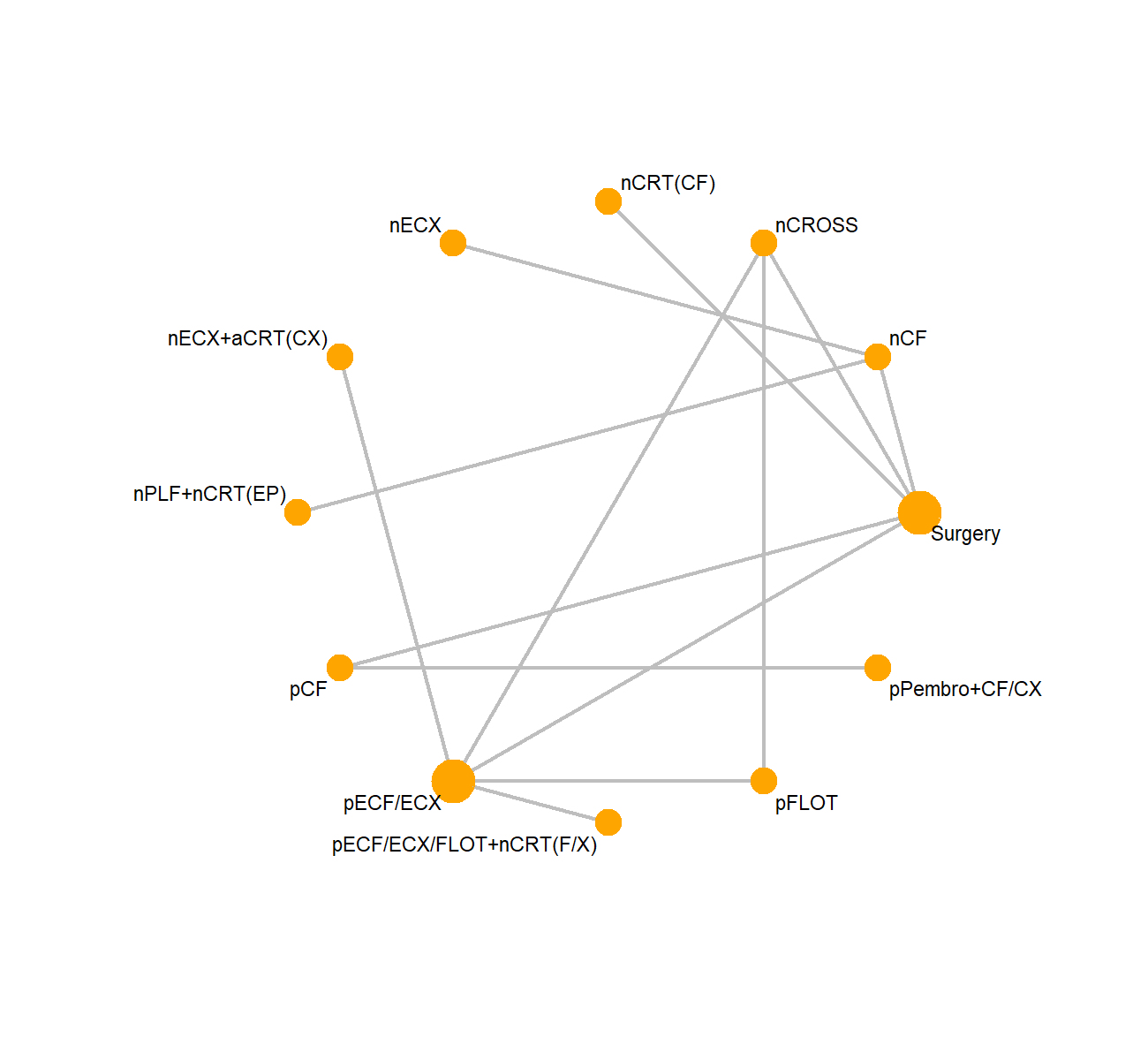
**

**
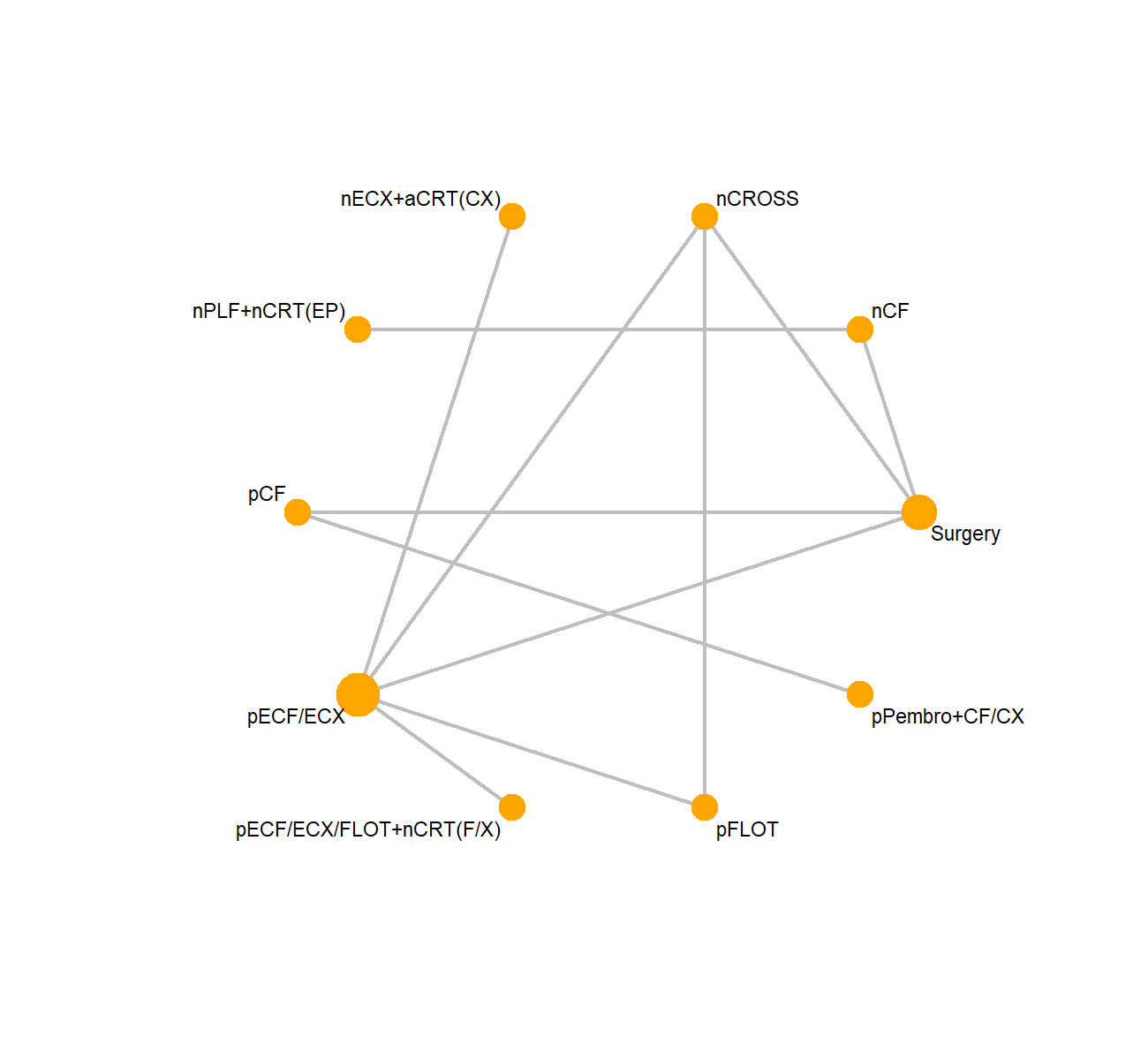
**

Each node represents a treatment. Each edge (line) represents direct comparison between the treatments/controls. Larger node and thicker edge represent relatively higher number of trials for that comparison.

Abbreviations: nECX: neoadjuvant epirubicin, cisplatin, and capecitabine; nCRT(CF): neoadjuvant cisplatin and fluorouracil with radiotherapy; nCROSS: neoadjuvant paclitaxel and carboplatin with radiotherapy; nCF: neoadjuvant cisplatin and fluorouracil; pPembro+CF/CX: perioperative pembrolizumab with cisplatin and fluorouracil/capecitabine; pFLOT: perioperative fluorouracil, leucovorin, oxaliplatin, and docetaxel; pECF/ECX/FLOT+nCRT(F/X): either perioperative epirubicin with cisplatin and fluorouracil/capecitabine, or perioperative fluorouracil, leucovorin, oxaliplatin, and docetaxel, along with neoadjuvant chemoradiotherapy (fluorouracil/capecitabine and radiotherapy); pECF/ECX: perioperative epirubicin, cisplatin, and fluorouracil/capecitabine; pCF: perioperative cisplatin and fluorouracil; nPLF+nCRT(EP): neoadjuvant cisplatin, leucovorin, and fluorouracil for induction, followed by etoposide and cisplatin with concomitant radiotherapy; nECX+aCRT(CX): neoadjuvant epirubicin, cisplatin/oxaliplatin and capecitabine, followed by adjuvant chemoradiotherapy with radiotherapy, cisplatin and capecitabine

# **Supplementary Figure 12: Network plot for overall survival in the overall population, with data for TOPGEAR trial stratified according to each treatment combination administered**


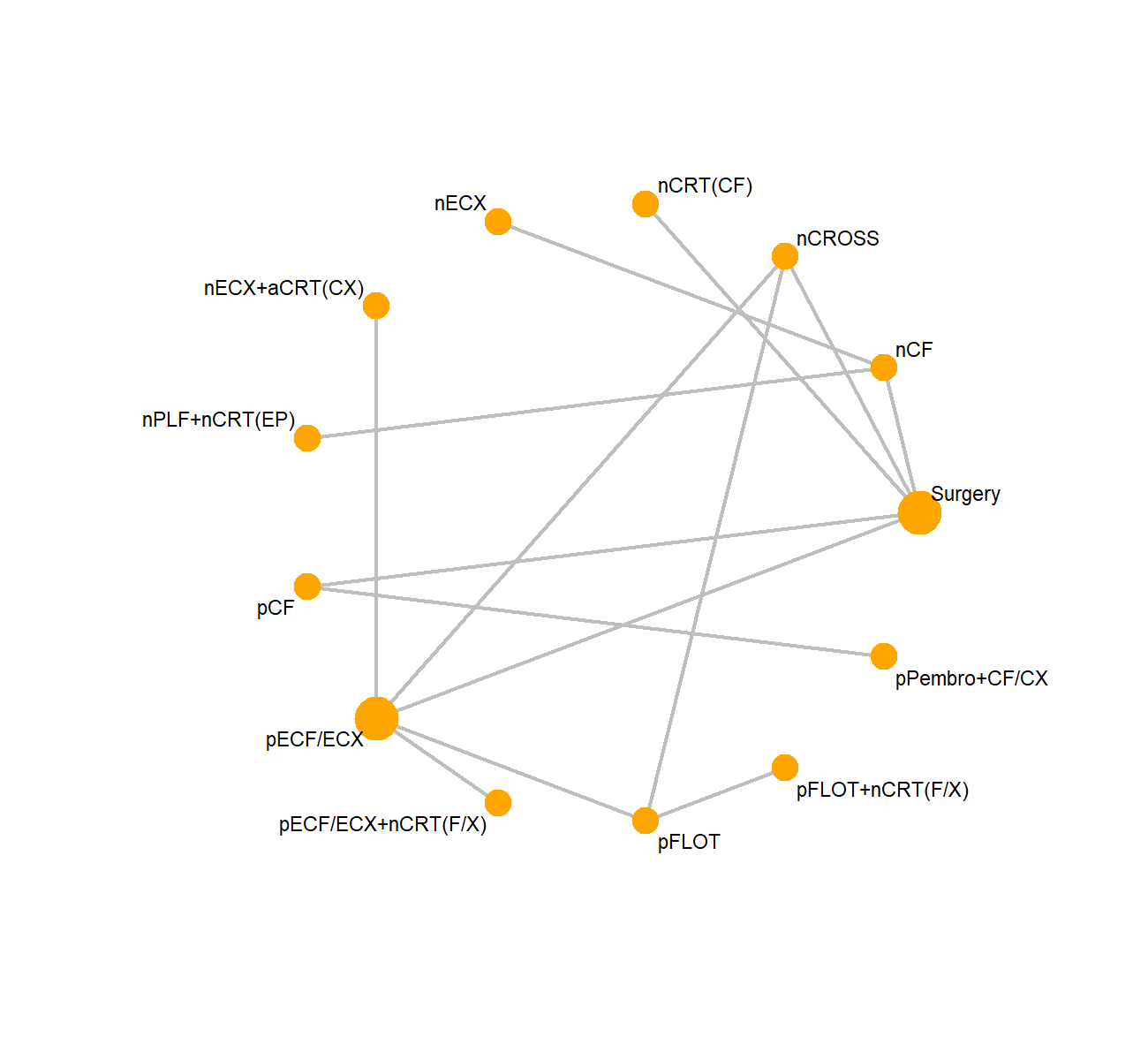


Each node represents a treatment. Each edge (line) represents direct comparison between the treatments/controls. Larger node and thicker edge represent relatively higher number of trials for that comparison.

Abbreviations: nECX: neoadjuvant epirubicin, cisplatin, and capecitabine; nCRT(CF): neoadjuvant cisplatin and fluorouracil with radiotherapy; nCROSS: neoadjuvant paclitaxel and carboplatin with radiotherapy; nCF: neoadjuvant cisplatin and fluorouracil; pPembro+CF/CX: perioperative pembrolizumab with cisplatin and fluorouracil/capecitabine; pFLOT+nCRT(F/X): perioperative fluorouracil, leucovorin, oxaliplatin, and docetaxel, along with neoadjuvant chemoradiotherapy (fluorouracil/capecitabine and radiotherapy); pFLOT: perioperative fluorouracil, leucovorin, oxaliplatin, and docetaxel; pECF/ECX+nCRT(F/X): perioperative epirubicin with cisplatin and fluorouracil/capecitabine, along with neoadjuvant chemoradiotherapy (fluorouracil/capecitabine and radiotherapy); pECF/ECX: perioperative epirubicin, cisplatin, and fluorouracil/capecitabine; pCF: perioperative cisplatin and fluorouracil; nPLF+nCRT(EP): neoadjuvant cisplatin, leucovorin, and fluorouracil for induction, followed by etoposide and cisplatin with concomitant radiotherapy; nECX+aCRT(CX): neoadjuvant epirubicin, cisplatin/oxaliplatin and capecitabine, followed by adjuvant chemoradiotherapy with radiotherapy, cisplatin and capecitabine

# **Supplementary Figure 13: Network plot for overall survival in the overall population (left) and the GEJ cohort (right), excluding the POET trial**

**
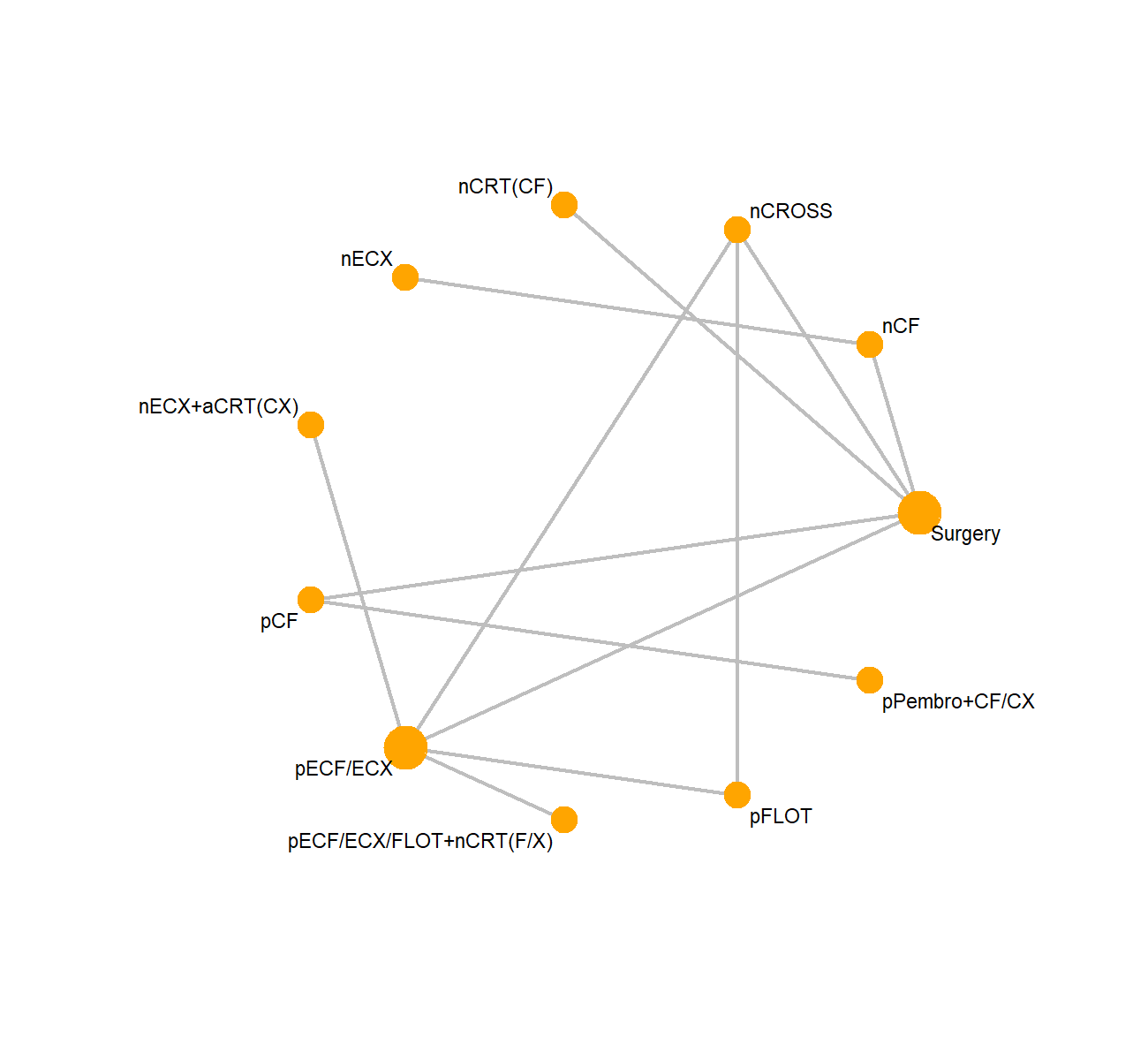
**

**
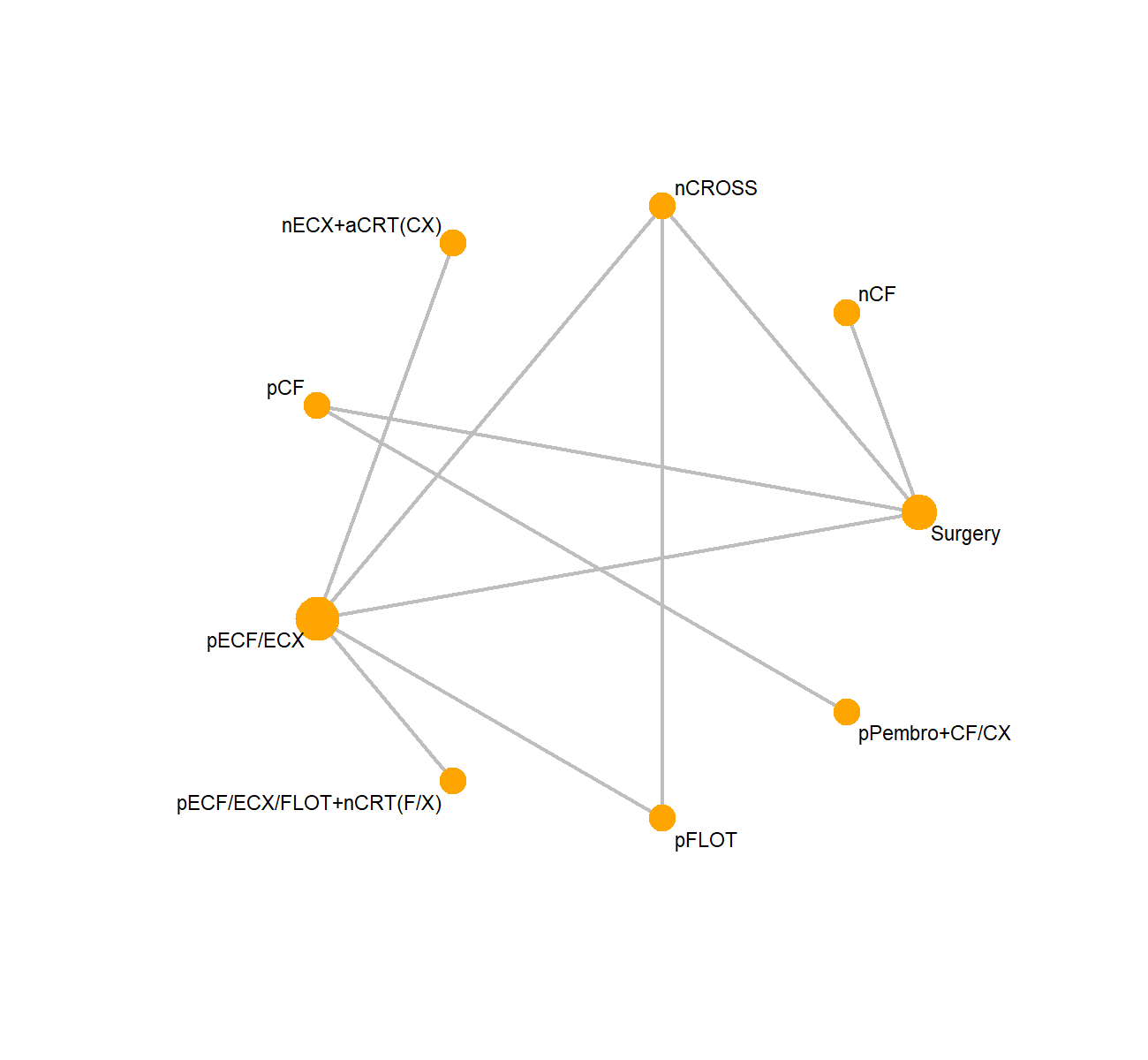
**

Each node represents a treatment. Each edge (line) represents direct comparison between the treatments/controls. Larger node and thicker edge represent relatively higher number of trials for that comparison.

Abbreviations: nECX: neoadjuvant epirubicin, cisplatin, and capecitabine; nCRT(CF): neoadjuvant cisplatin and fluorouracil with radiotherapy; nCROSS: neoadjuvant paclitaxel and carboplatin with radiotherapy; nCF: neoadjuvant cisplatin and fluorouracil; pPembro+CF/CX: perioperative pembrolizumab with cisplatin and fluorouracil/capecitabine; pFLOT: perioperative fluorouracil, leucovorin, oxaliplatin, and docetaxel; pECF/ECX/FLOT+nCRT(F/X): either perioperative epirubicin with cisplatin and fluorouracil/capecitabine, or perioperative fluorouracil, leucovorin, oxaliplatin, and docetaxel, along with neoadjuvant chemoradiotherapy (fluorouracil/capecitabine and radiotherapy); pECF/ECX: perioperative epirubicin, cisplatin, and fluorouracil/capecitabine; pCF: perioperative cisplatin and fluorouracil; nECX+aCRT(CX): neoadjuvant epirubicin, cisplatin/oxaliplatin and capecitabine, followed by adjuvant chemoradiotherapy with radiotherapy, cisplatin and capecitabine

# **Supplementary Figure 14: Mixed treatment comparisons for overall survival in the overall population**

**
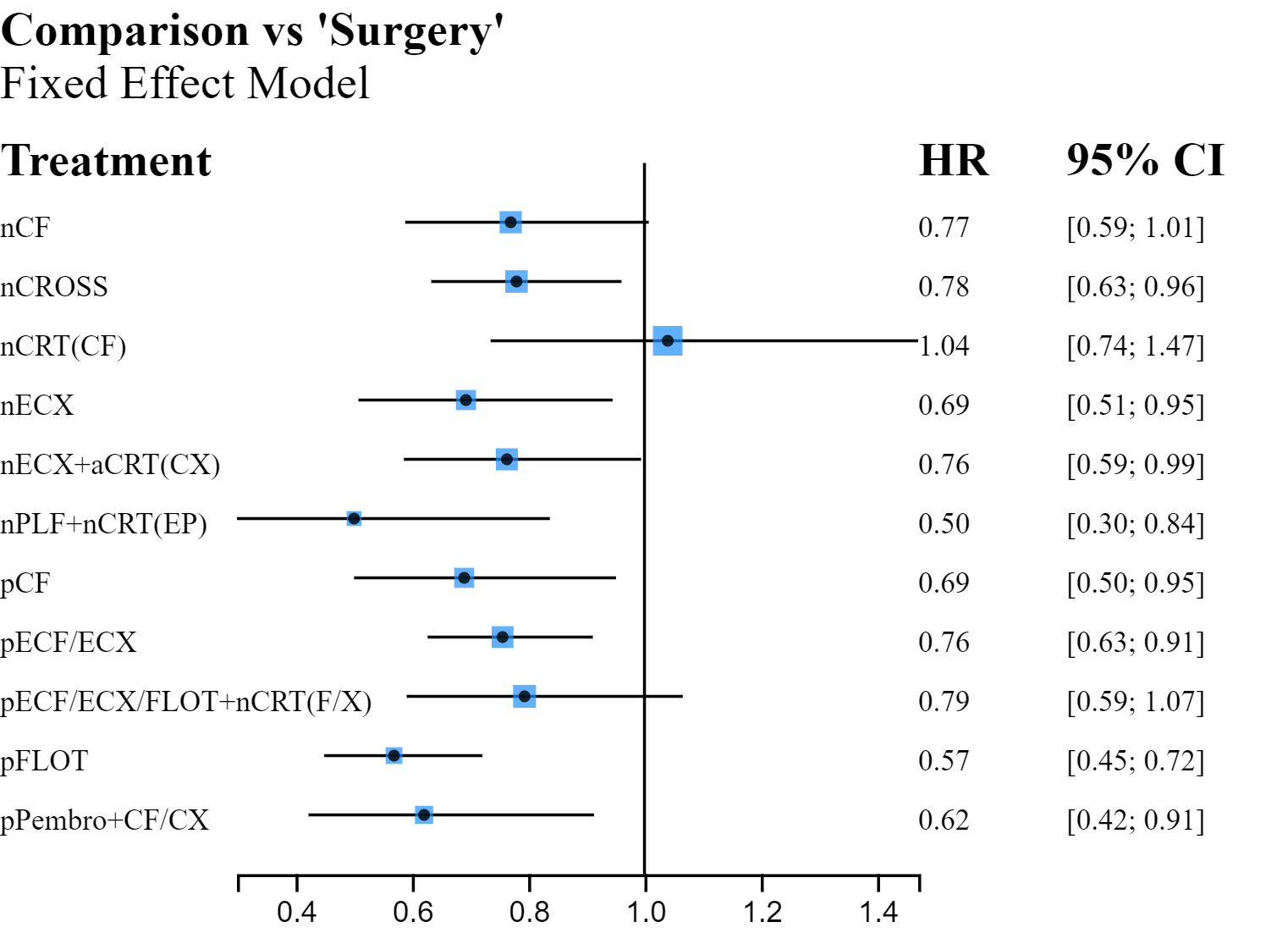
**

**
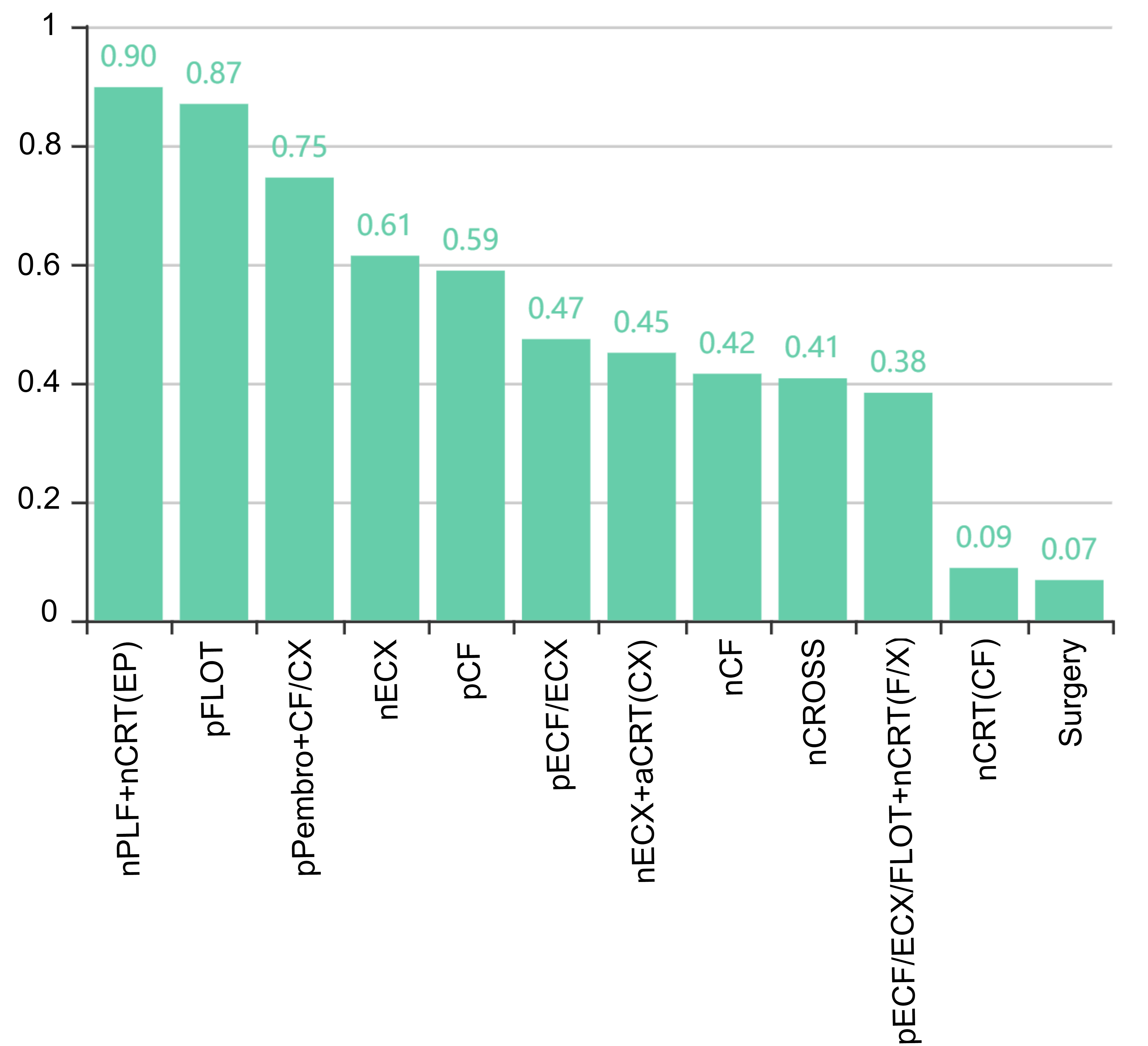
**

# **Supplementary Figure 15: Mixed treatment comparisons for overall survival in the overall population, with data for TOPGEAR trial stratified according to each treatment combination administered**


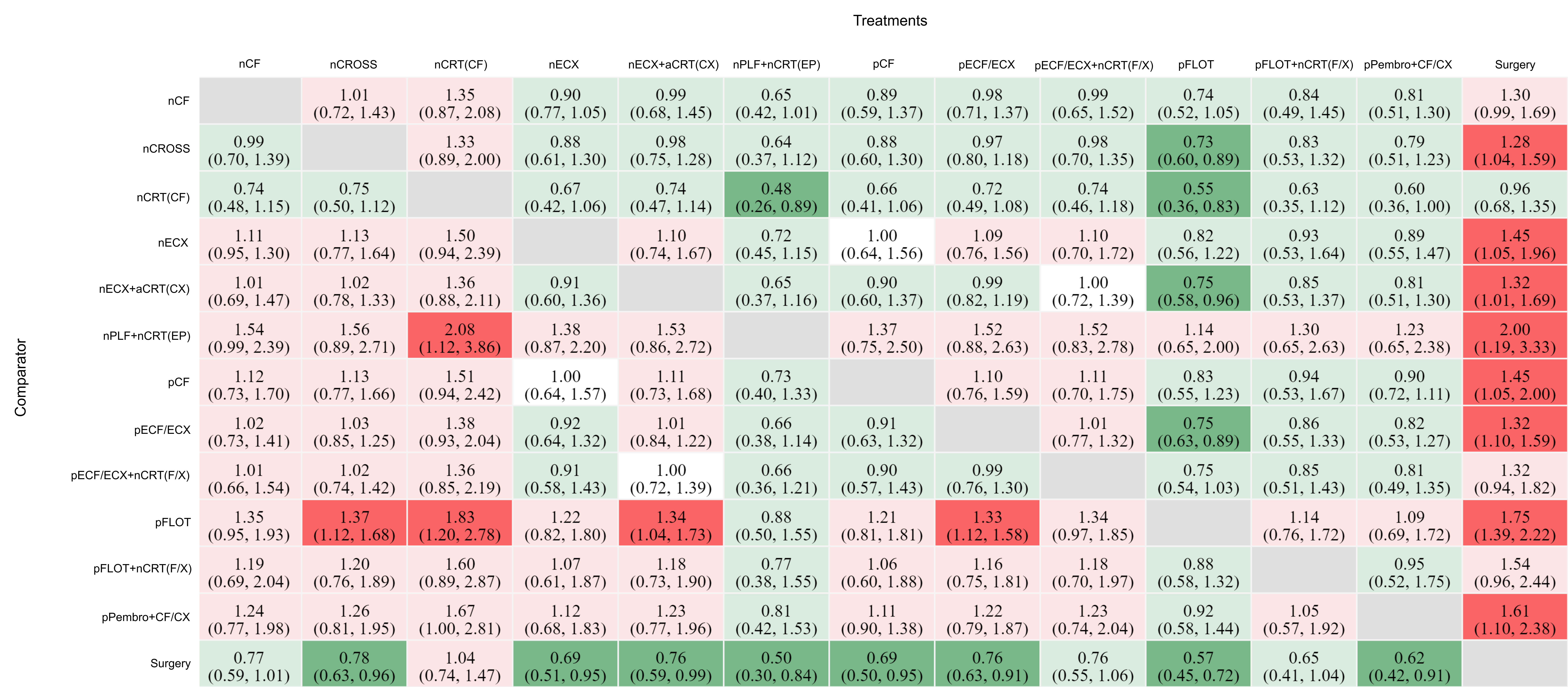


The values in each cell represent the relative treatment effect (and 95% CI) of the treatment on the top, compared to the treatment on the left. Green color suggests relative treatment benefit. Light green suggests non-significant benefit and dark green suggests significant benefit. Red color suggests relative treatment harm. Light red suggests non-significant harm and dark red suggests significant harm.


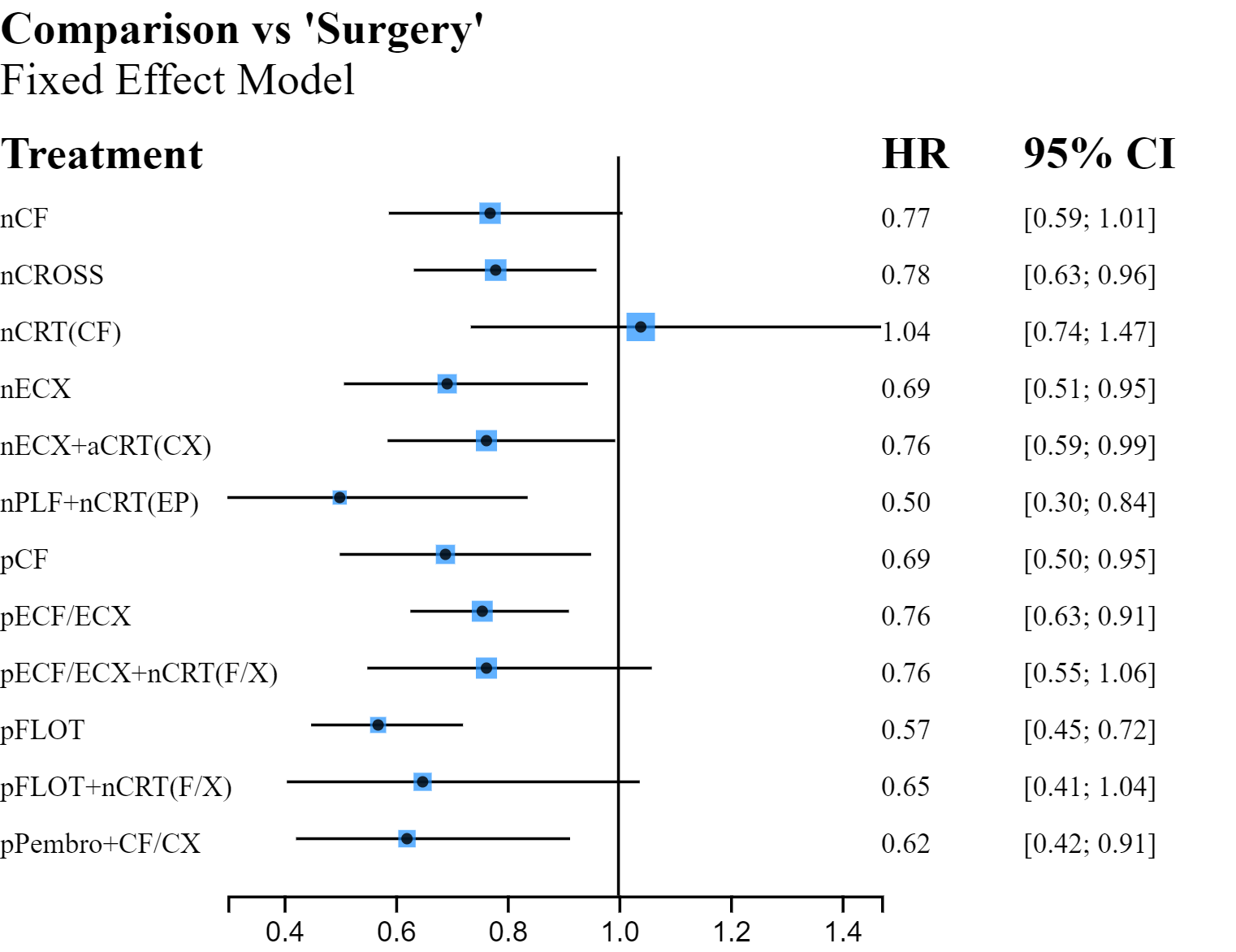


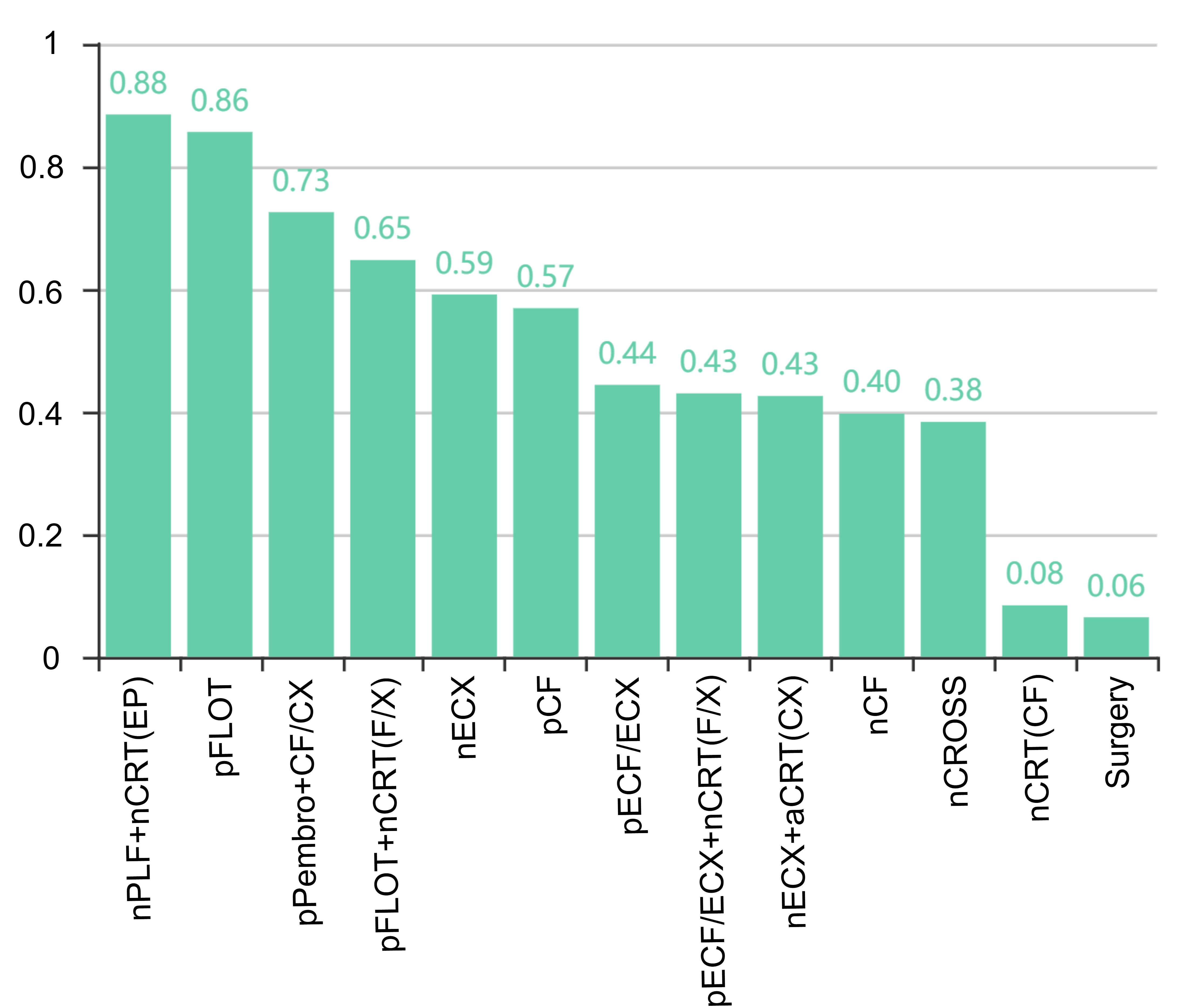


# **Supplementary Figure 16: Mixed treatment comparisons for overall survival in the overall population, excluding the POET trial**

**
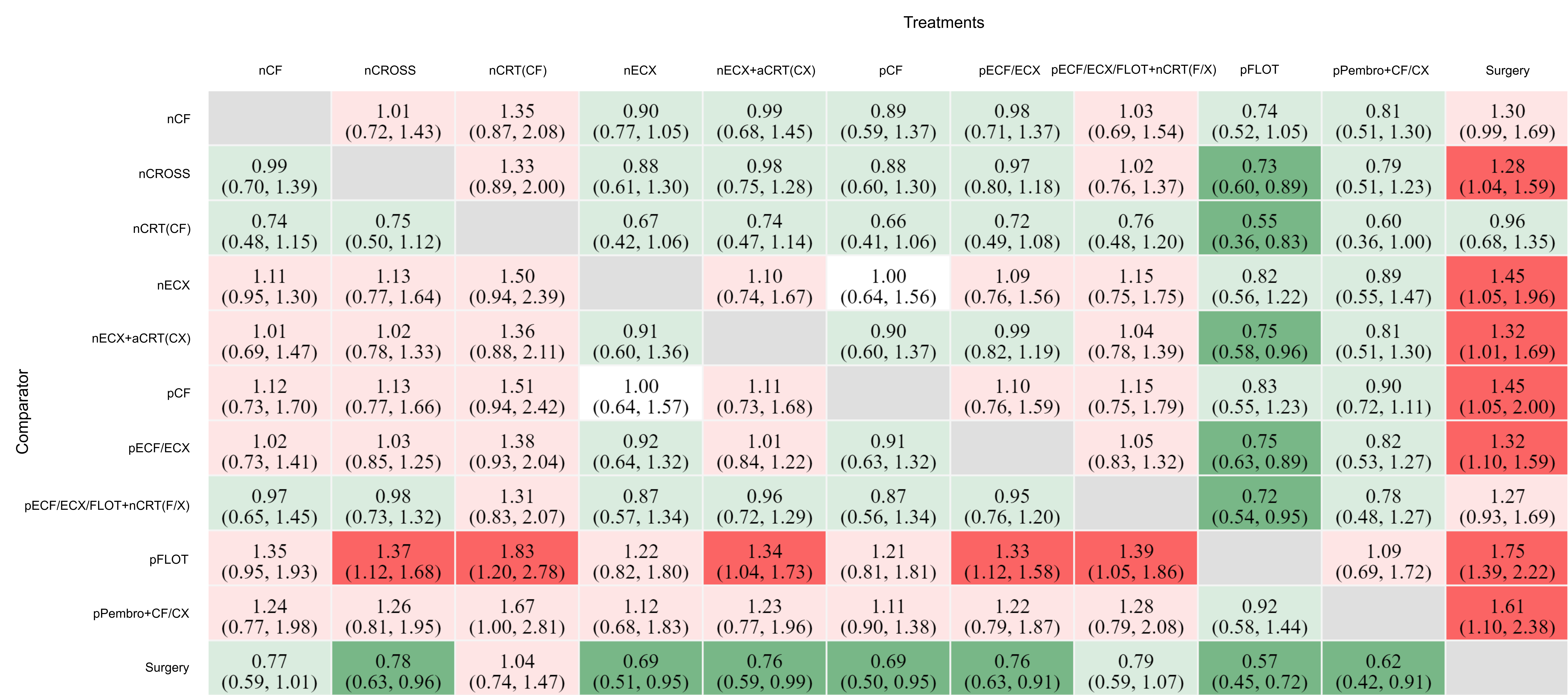
**

The values in each cell represent the relative treatment effect (and 95% CI) of the treatment on the top, compared to the treatment on the left. Green color suggests relative treatment benefit. Light green suggests non-significant benefit and dark green suggests significant benefit. Red color suggests relative treatment harm. Light red suggests non-significant harm and dark red suggests significant harm.


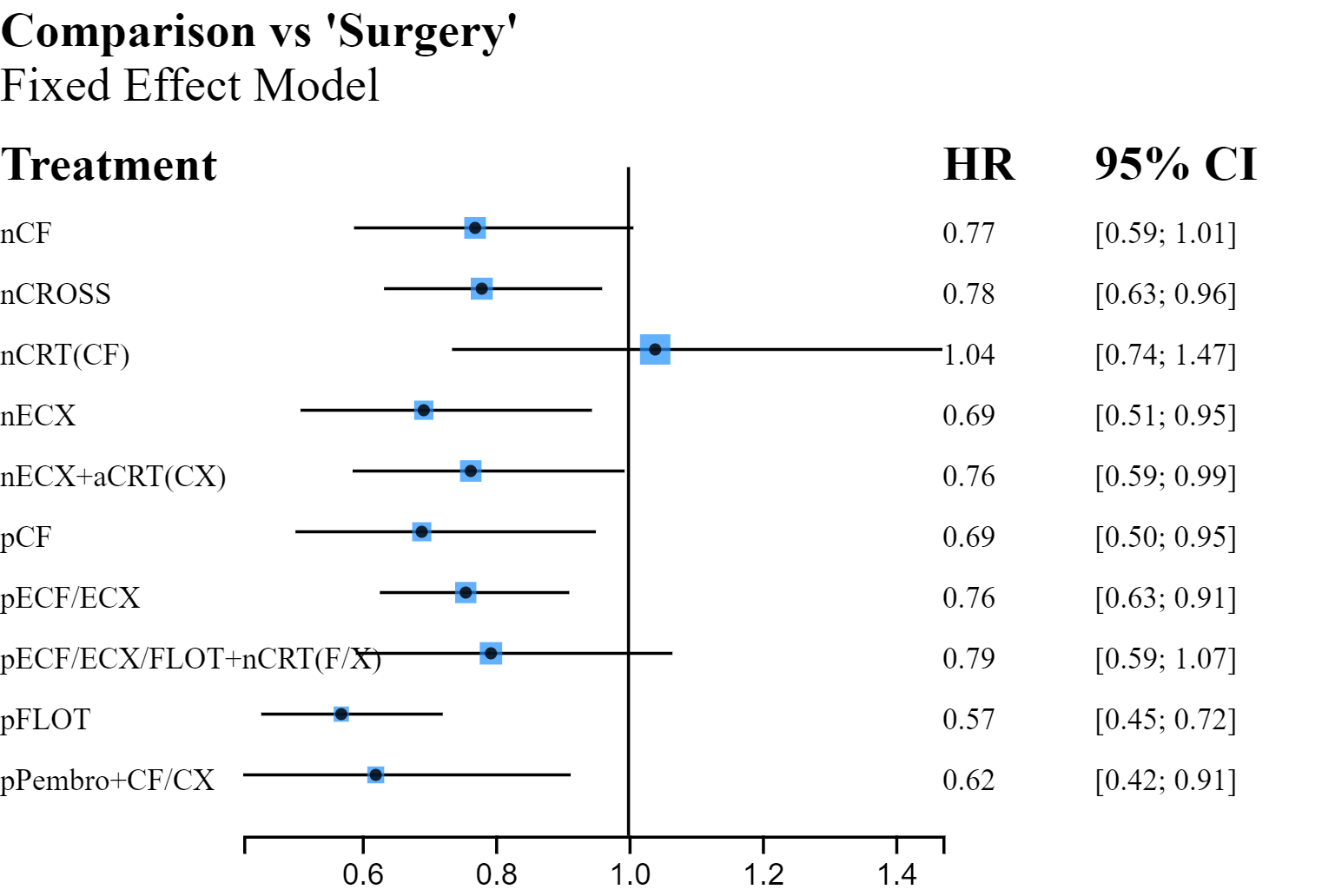


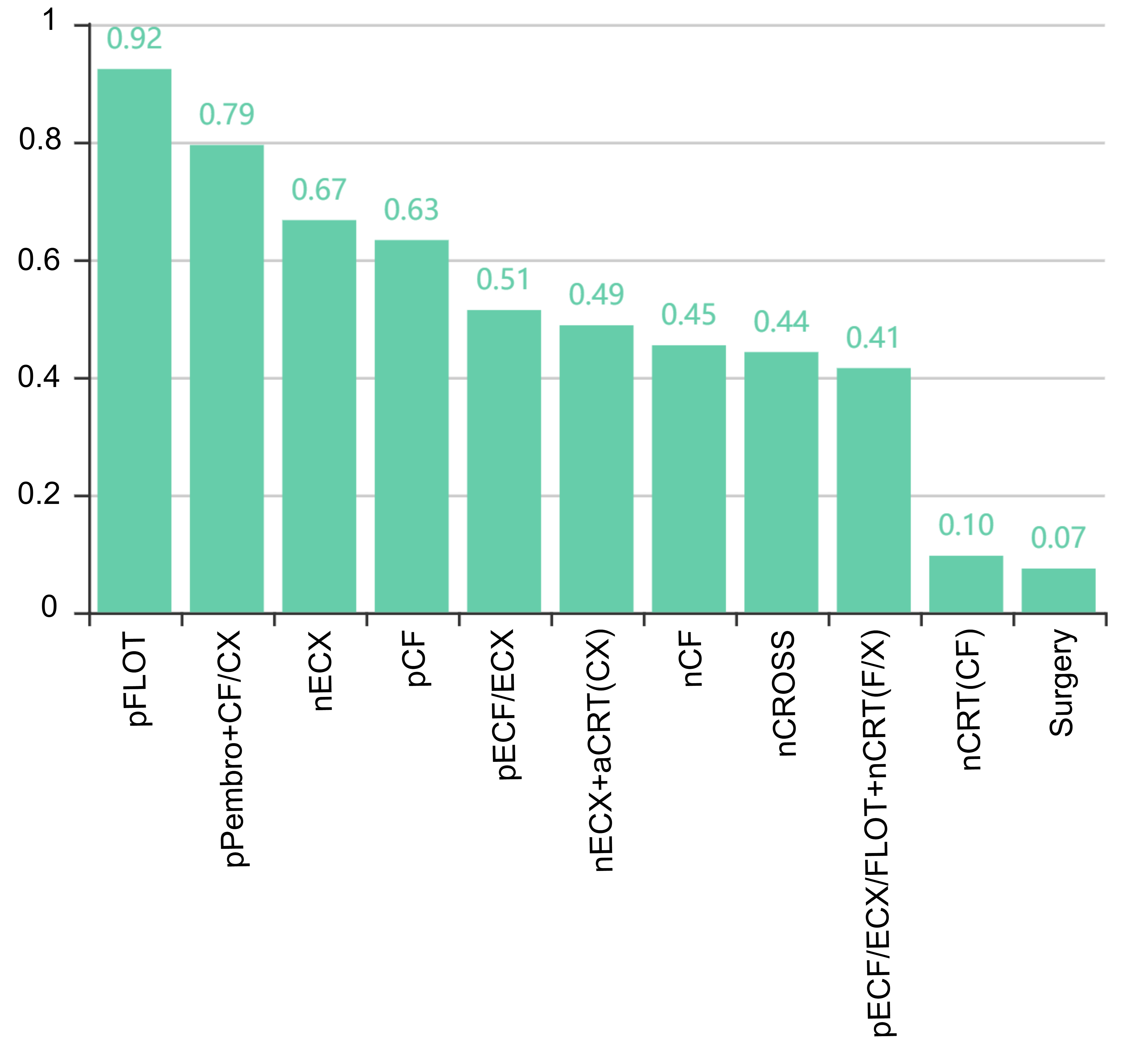


# **Supplementary Figure 17: Mixed treatment comparisons for overall survival in the GEJ cohort**


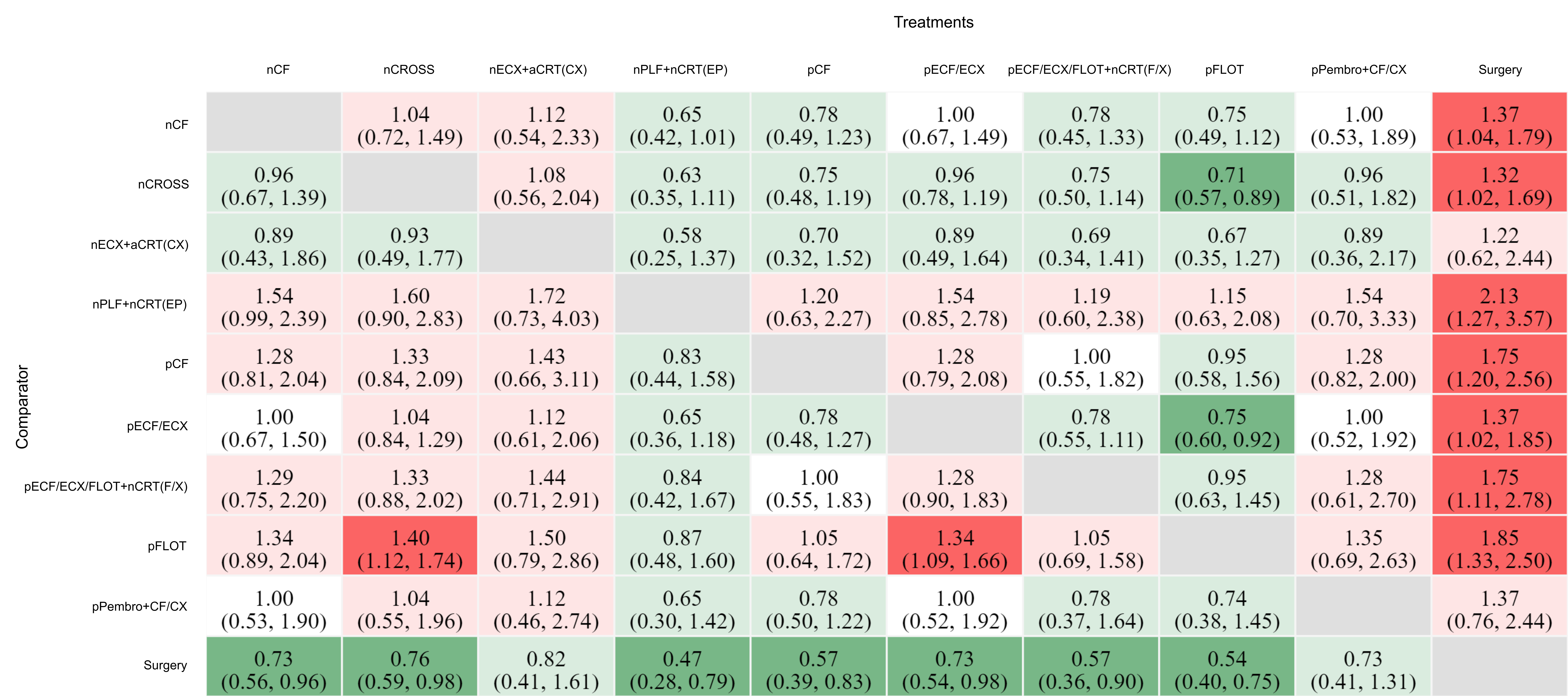


The values in each cell represent the relative treatment effect (and 95% CI) of the treatment on the top, compared to the treatment on the left. Green color suggests relative treatment benefit. Light green suggests non-significant benefit and dark green suggests significant benefit. Red color suggests relative treatment harm. Light red suggests non-significant harm and dark red suggests significant harm.


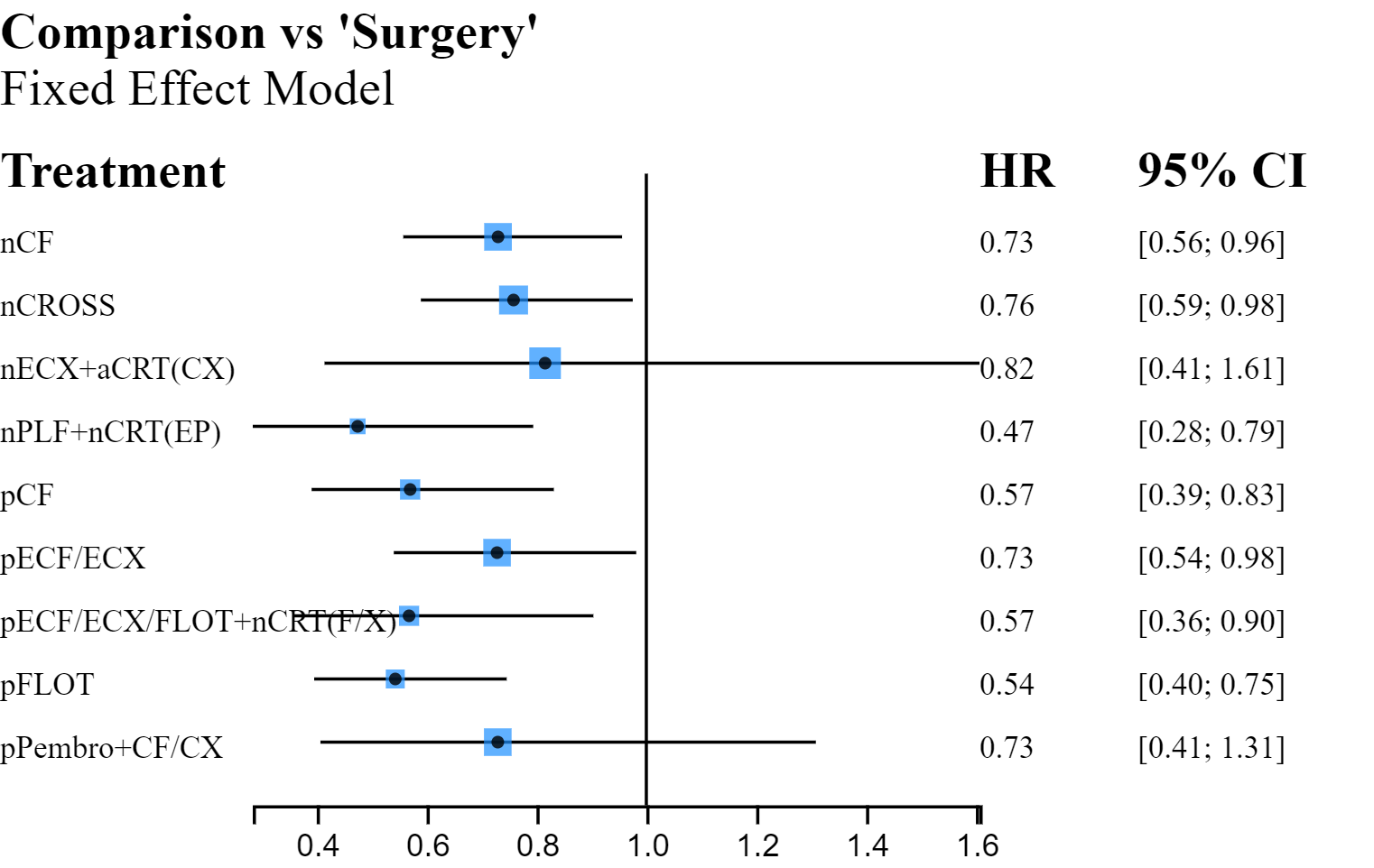

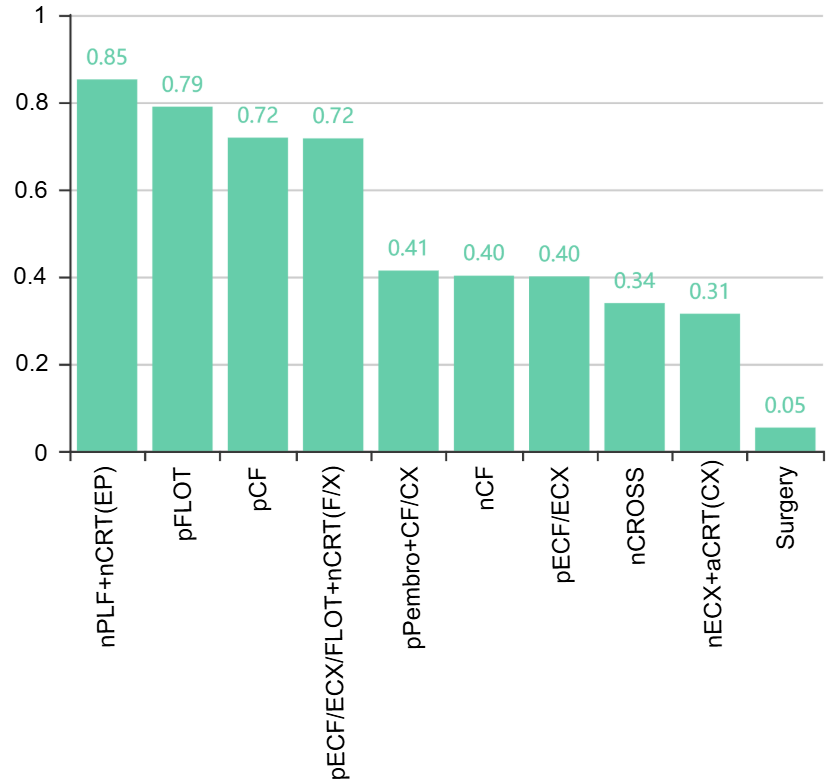


# **Supplementary Figure 18: Mixed treatment comparisons for overall survival in the GEJ cohort excluding the POET trial**

**
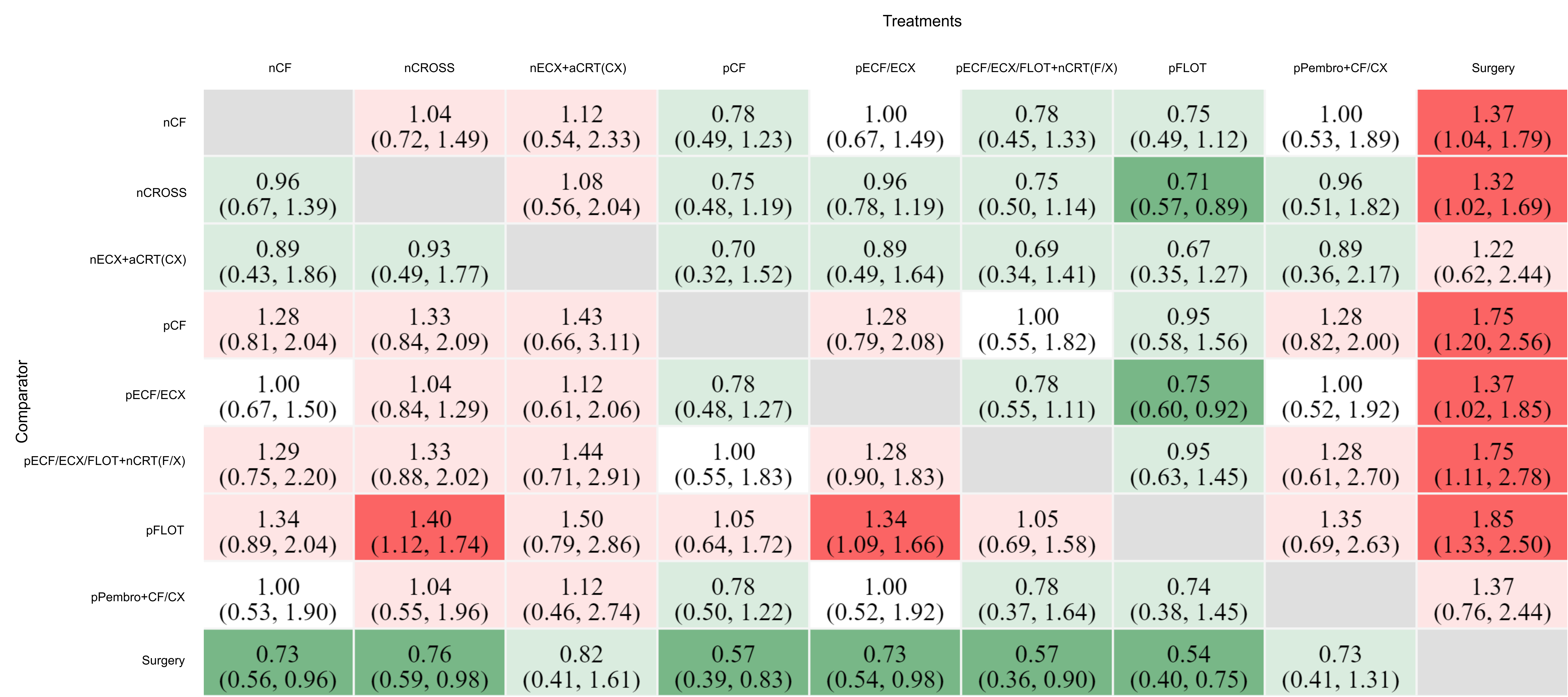
**

The values in each cell represent the relative treatment effect (and 95% CI) of the treatment on the top, compared to the treatment on the left. Green color suggests relative treatment benefit. Light green suggests non-significant benefit and dark green suggests significant benefit. Red color suggests relative treatment harm. Light red suggests non-significant harm and dark red suggests significant harm.


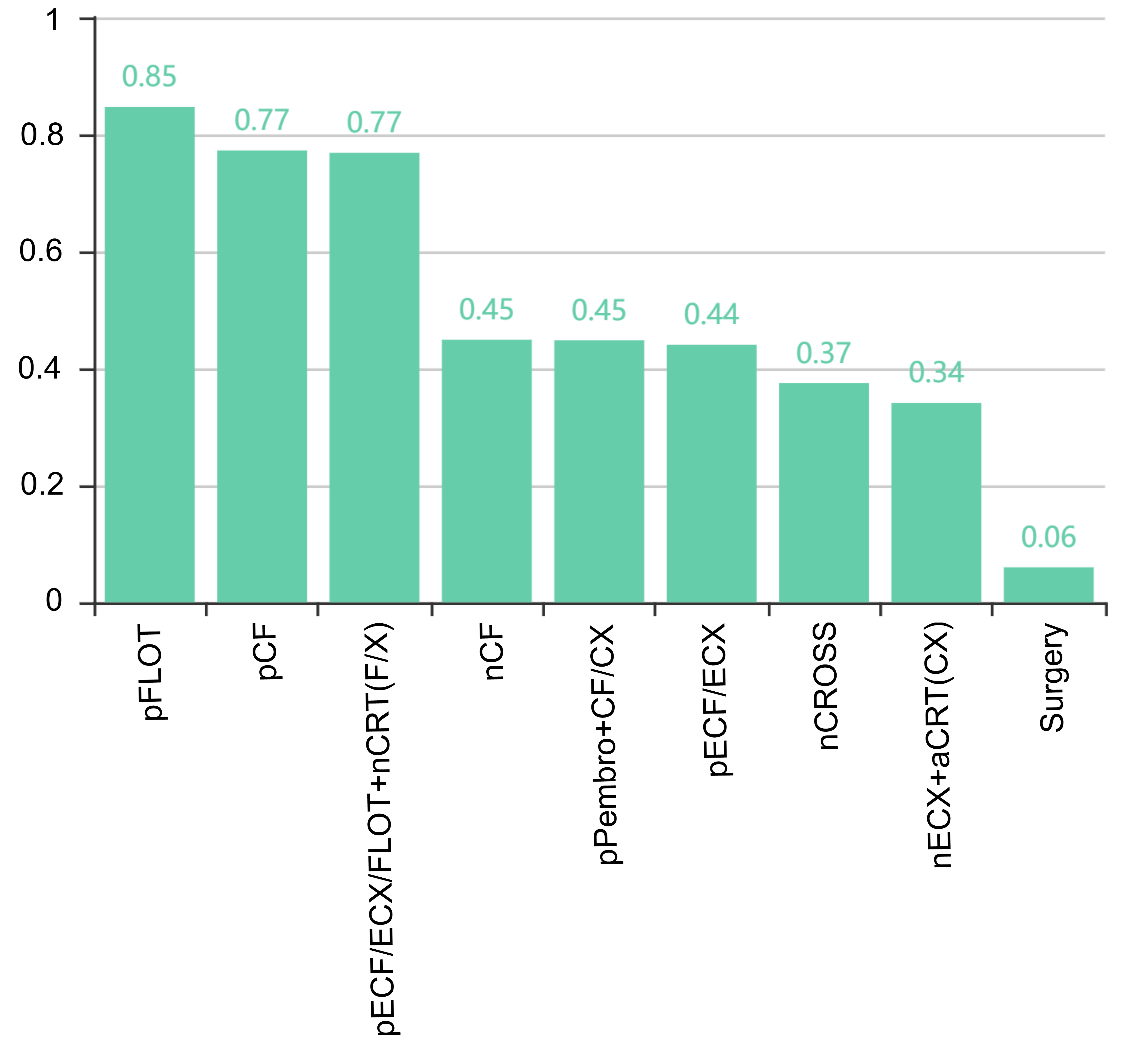

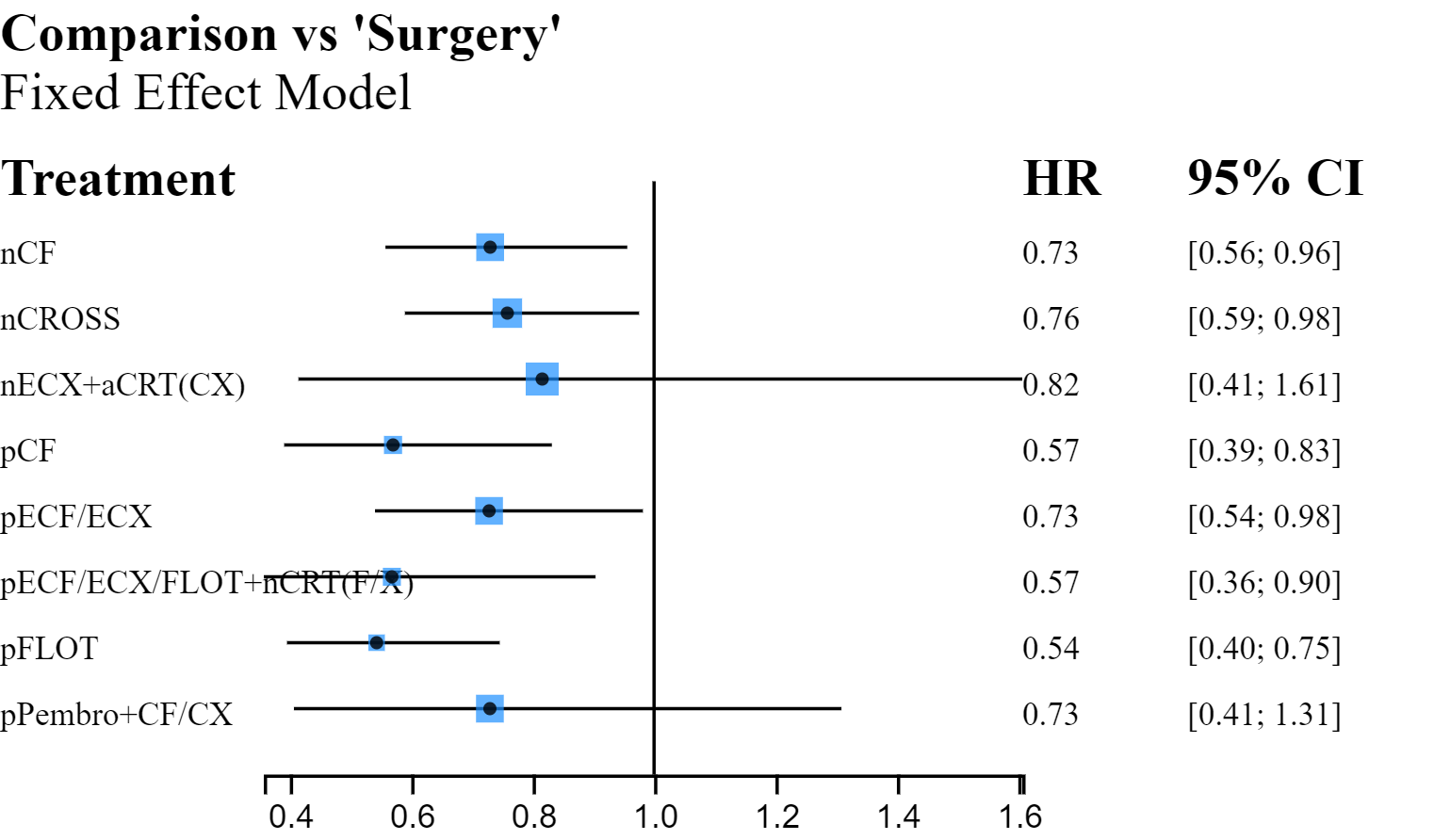


# **Supplementary Figure 19: Network plot for pathologic complete response in the overall population, excluding the UK MRCOE05 and KEYNOTE-585 trials**


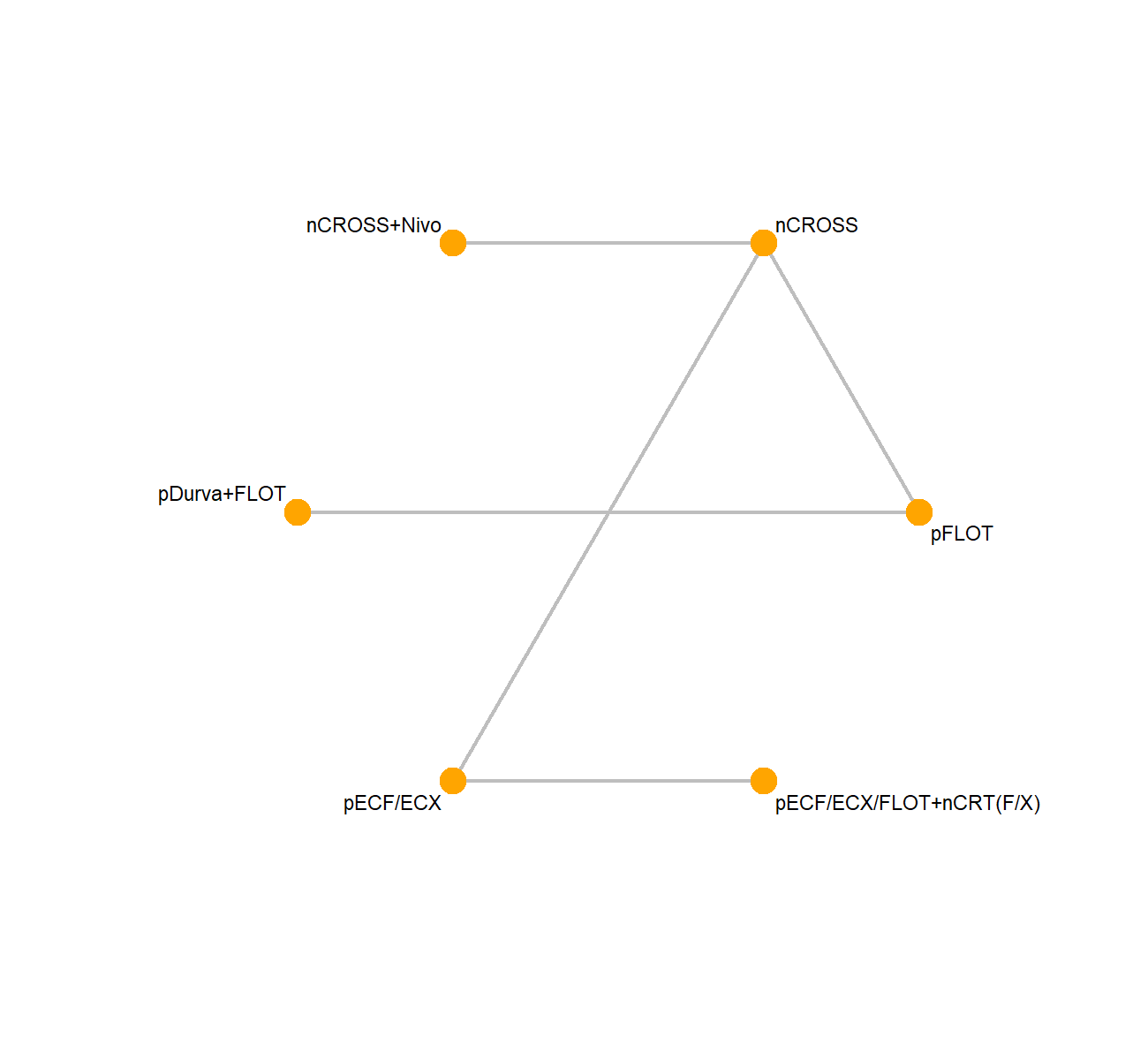


Each node represents a treatment. Each edge (line) represents direct comparison between the treatments/controls. Larger node and thicker edge represent relatively higher number of trials for that comparison.

Abbreviations: nCROSS+Nivo: neoadjuvant paclitaxel and carboplatin with radiotherapy, plus nivolumab; nCROSS: neoadjuvant paclitaxel and carboplatin with radiotherapy; pFLOT: perioperative fluorouracil, leucovorin, oxaliplatin, and docetaxel; pECF/ECX/FLOT+nCRT(F/X): either perioperative epirubicin with cisplatin and fluorouracil/capecitabine, or perioperative fluorouracil, leucovorin, oxaliplatin, and docetaxel, along with neoadjuvant chemoradiotherapy (fluorouracil/capecitabine and radiotherapy); pECF/ECX: perioperative epirubicin, cisplatin, and fluorouracil/capecitabine; pDurva+FLOT: perioperative durvalumab with fluorouracil, leucovorin, oxaliplatin and docetaxel

# **Supplementary Figure 20: Mixed treatment comparisons for pathologic complete response in the overall population, excluding the UK MRCOE05 and KEYNOTE-585 trials**


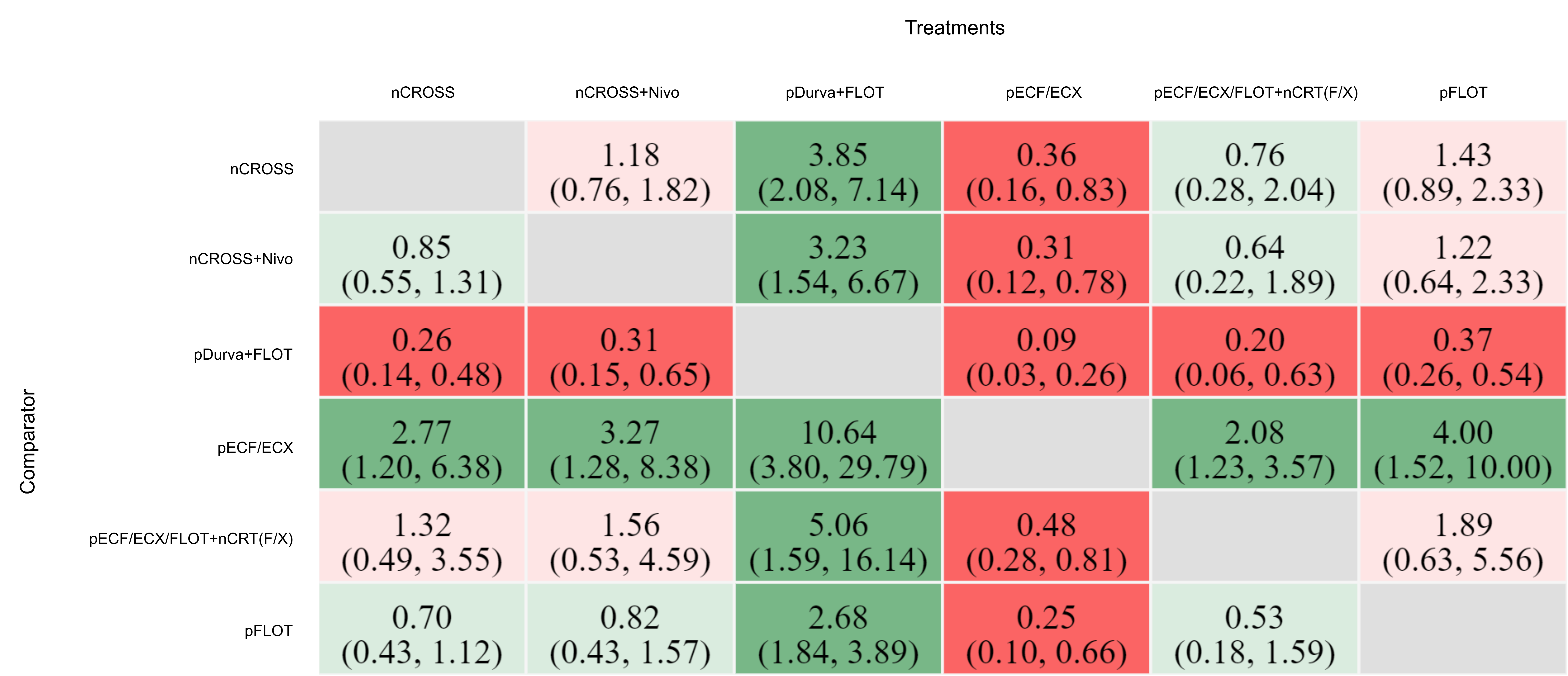


The values in each cell represent the relative treatment effect (and 95% CI) of the treatment on the top, compared to the treatment on the left. Green color suggests relative treatment benefit. Light green suggests non-significant benefit and dark green suggests significant benefit. Red color suggests relative treatment harm. Light red suggests non-significant harm and dark red suggests significant harm.


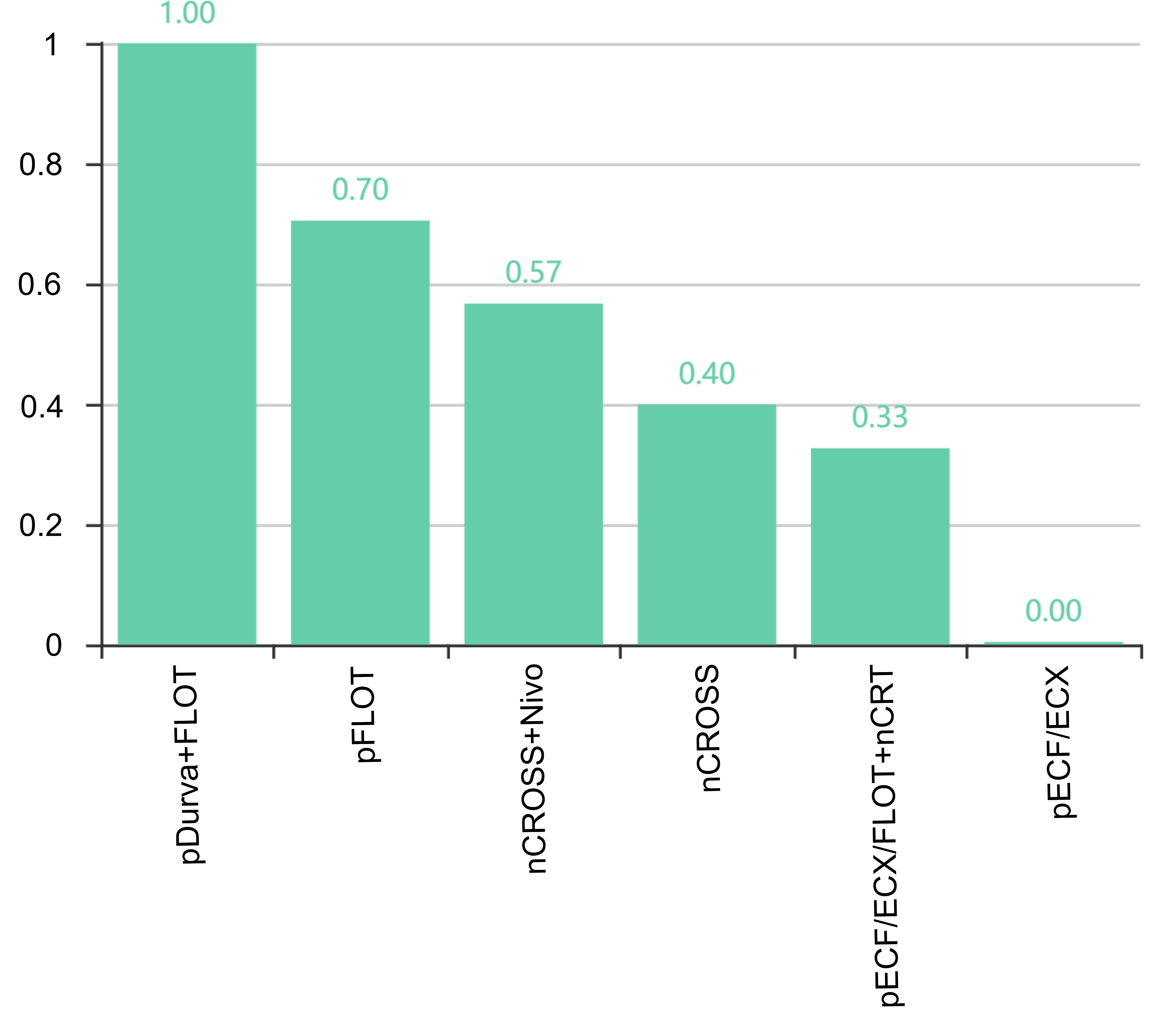


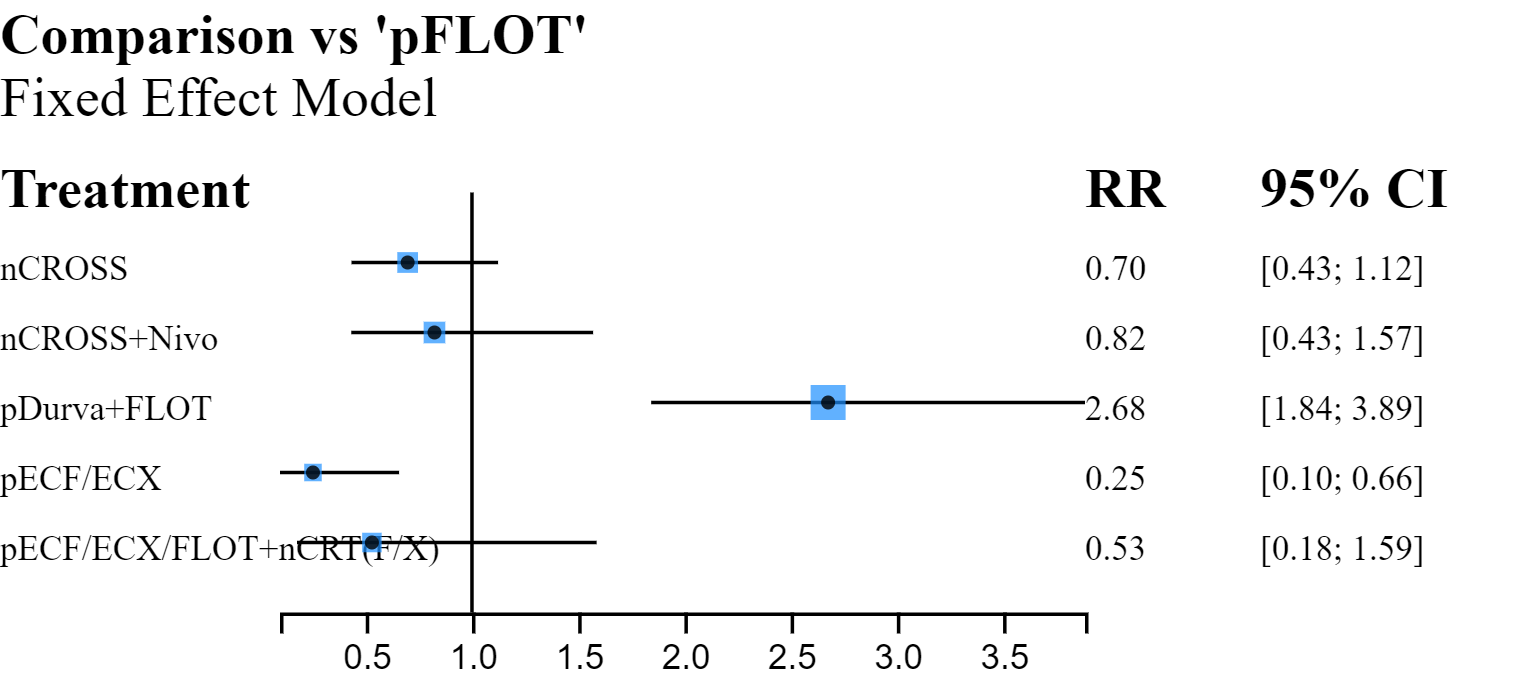


# **Supplementary Table 1: Additional patient characteristics**

| **Trial** | **Arm** | **Tumor Site \|  N (%)** | **Tumor Histology \|**  **N (%)** | **R0 resection \| N (%)** | **ypT0 \|  N (%)** | **ypN0 \|**  **N (%)** | **cT staging \| N (%)** | **cN staging \| N (%)** |
| --- | --- | --- | --- | --- | --- | --- | --- | --- |
|  |  |  |  |  |  |  |  |  |
| **Perioperative Setting** | | | | | | | | |
| FLOT4 | pFLOT | GEJ Siewert type 1: 80 (23),  GEJ Siewert type 2/3: 118 (33),  Stomach: 158 (44) | Adenocarcinoma: 356 (100) | 301 (85) | NA | 174 (49) | cT1: 3 (1),  cT2: 49 (14),  cT3: 267 (75),  cT4: 28 (8) | cN0: 77 (22),  cN+: 279 (78) |
|  | pECF/ECX | GEJ Siewert type 1: 85 (24),  GEJ Siewert type 2/3: 115 (32),  Stomach: 160 (44) | Adenocarcinoma: 360 (100) | 279 (78) | NA | 146 (41) | cT1: 2 (1),  cT2: 59 (16),  cT3: 253 (70),  cT4: 33 (9) | cN0: 70 (19),  cN+: 290 (81) |
| KEYNOTE-585 | pPembro+CF/CX | GEJ: 86 (21),  Stomach: 316 (79) | Adenocarcinoma: 402 (100) | NA | NA | NA | cT1: 1 (<1),  cT2: 23 (6),  cT3: 211 (53),  cT4: 9 (2),  cT4a: 142 (35),  cT4b: 15 (4) | cN0: 74 (18),  cN1: 177 (44),  cN2: 119 (30),  cN3: 27 (7) |
|  | Placebo+pCF/CX | GEJ: 79 (20),  Stomach: 322 (80) | Adenocarcinoma: 402 (100) | NA | NA | NA | cT1: 2 (<1),  cT2: 10 (2),  cT3: 219 (54),  cT4: 10 (2),  cT4a: 145 (36),  cT4b: 14 (3) | cN0: 70 (17),  cN1: 168 (42),  cN2: 132 (33),  cN3: 29 (7) |
| MAGIC | pECF | Lower esophagus: 37 (15),  GEJ: 28 (11),  Stomach: 185 (74) | Adenocarcinoma: 250 (100) | NA | NA | NA | NA | NA |
|  | Surgery alone | Lower esophagus: 36 (14),  GEJ: 30 (12),  Stomach 187 (74) | Adenocarcinoma: 253 (100) | NA | NA | NA | NA | NA |
| FNCLCC/FFCD Study | pCF | Lower esophagus: 15 (13),  GEJ: 70 (62),  Stomach: 28 (25) | Adenocarcinoma: 113 (100) | 95 (87) | NA | NA | NA | NA |
|  | Surgery alone | Lower esophagus: 10 (9),  GEJ: 74 (67),  Stomach: 27 (24) | Adenocarcinoma: 111 (100) | 81 (74) | NA | NA | NA | NA |
| MATTERHORN | pDurva+FLOT | Stomach: ~322 (68) | Adenocarcinoma: 474 (100) | 369 (86) | NA | NA | cT3: ~626 (66),  cT4: ~119 (25) | NA |
|  | Placebo+pFLOT | Stomach: ~322 (68) | Adenocarcinoma: 474 (100) | 362 (86) | NA | NA | cT3: ~626 (66), cT4: ~119 (25) | NA |
| TOPGEAR | pECF/ECX/FLOT+nCRT(F/X) | GEJ: 98 (34),  Upper/middle third stomach: 80 (28),  Lower third stomach: 72 (25),  Multiple sites: 36 (13) | Adenocarcinoma: 286 (100) | 208 (92) | 34 15) | 125 (54) | cT1/T2: 33 (12), cT3/T4: 252 (88) | cN0: 110 (38),  cN+: 176 (62) |
|  | pECF/ECX/FLOT | GEJ: 101 (35),  Upper/middle third stomach: 89 (31),  Lower third stomach: 70 (24),  Multiple sites: 28 (10) | Adenocarcinoma: 288 (100) | 206 (88) | 17 (7) | 104 (42) | cT1/T2: 31 (11), cT3/T4: 254 (88) | cN0: 111 (39),  cN+: 177 (61) |
| CRITICS | nECX+aCRT(CX) | GEJ: 67 (17),  Stomach: 328 (83) | Adenocarcinoma: 395 (100) | 267 (82) | 20 (6) | 161 (49) | NA | NA |
|  | pECX | GEJ: 68 (17),  Stomach: 325 (83) | Adenocarcinoma: 393 (100) | 248 (80) | 20 (6) | 150 (48) | NA | NA |
| **Neoadjuvant Setting** | | | | | | | | |
| CROSS | nCROSS | Esophagus: 133 (75),  GEJ: 39 (22) | Adenocarcinoma: 134 (75), Squamous cell carcinoma: 41 (23) | 148 (92) | 47 (29) | | cT1: 1 (1),  cT2: 26 (15),  cT3: 150 (84),  cT4: 0 (0) | cN0: 59 (33),  cN1: 116 (65) |
|  | Surgery alone | Esophagus: 135 (72),  GEJ: 49 (26) | Adenocarcinoma: 141 (75), Squamous cell carcinoma: 43 (23) | 111 (69) | 0 (0) | | cT1: 1 (1),  cT2: 35 (19),  cT3: 147 (78),  cT4: 1 (1) | cN0: 58 (31),  cN1: 120 (64) |
| Burmeister BH et al | nCRT(CF) | Upper/middle third esophagus: 29 (23),  Lower third esophagus: 99 (77) | Adenocarcinoma: 80 (63), Squamous cell carcinoma: 45 (35) | 103 (80) | NA | NA | NA | NA |
|  | Surgery alone | Upper/middle third esophagus: 24 (19),  Lower third esophagus: 104 (81) | Adenocarcinoma: 78 (61), Squamous cell carcinoma: 50 (39) | 76 (59) | NA | NA | NA | NA |
| POET | nPLF+nCRT(EP) | GEJ Siewert type 1: 33 (55),  GEJ Siewert type 2/3: 27 (45) | Adenocarcinoma: 60 (100) | 43 (72) | NA | NA | cT3: 55 (92),  cT4: 5 (8) | NA |
|  | nPLF | GEJ Siewert type 1: 32 (54),  GEJ Siewert type 2/3: 27 (46) | Adenocarcinoma: 59 (100) | 41 (69) | NA | NA | cT3: 54 (92),  cT4: 5 (8) | NA |
| UK MRC OE05 | nECX | Middle esophagus: 56 (15),  GEJ Siewert type 1: 208 (57),  GEJ Siewert type 2: 89 (24) | Adenocarcinoma: 446 (100) | 223 (66) | 19 (5) | 142 (39) | cT2: 40 (9),  cT3: 389 (87),  cT4: 12 (3) | cN0: 97 (22),  cN1: 345 (77) |
|  | nCF | Middle esophagus: 72 (19),  GEJ Siewert type 1: 227 (59),  GEJ Siewert type 2: 76 (20) | Adenocarcinoma: 451 (100) | 212 (59) | 6 (2) | 115 (30) | cT2: 47 (10),  cT3: 386 (86),  cT4: 15 (3) | cN0: 97 (22),  cN1: 350 (78) |
| MRC OE02 | nCF | Upper third esophagus: 3 (1),  Middle third esophagus: 97 (24),  Lower third esophagus: 260 (65),  Stomach: 40 (10) | Adenocarcinoma: 265 (66), Squamous cell carcinoma: 123 (31), Undifferentiated: 11 (3) | 233 (60) | NA | NA | NA | NA |
|  | Surgery alone | Upper third esophagus: 4 (1),  Middle third esophagus: 102 (25), Lower third esophagus: 254 (63),  Stomach: 42 (10) | Adenocarcinoma: 268 (67), Squamous cell carcinoma: 124 (31), Undifferentiated: 10 (2) | 215 (54) | NA | NA | NA | NA |
| EA2174 | nCROSS+Nivo | Esophagus: 166 (60),  GEJ: 108 (39) | Adenocarcinoma: 137 (100) | NA | NA | NA | NA | NA |
|  | nCROSS |  | Adenocarcinoma: 138 (100) | NA | NA | NA | NA | NA |
| **Perioperative with Neoadjuvant Setting** | | | | | | | | |
| Neo-AEGIS | pECF ^a^ | GEJ Siewert type 1: 123 (67),  GEJ Siewert type 2: 46 (25),  GEJ Siewert type 3: 15 (8) | Adenocarcinoma: 184 (100) | 119 (82) | 7 (4) | 71 (44) | cT2: 29 (16),  cT3: 155 (84) | cN0: 73 (40),  cN1: 83 (45),  cN2: 23 (13),  cN3: 5 (3) |
|  | nCROSS | GEJ Siewert type 1: 126 (71),  GEJ Siewert type 2: 38 (21),  GEJ Siewert type 3: 14 (8) | Adenocarcinoma: 178 (100) | 131 (96) | 23 (14) | 100 (60) | cT2: 28 (16),  cT3: 150 (84) | cN0: 78 (44),  cN1: 73 (41),  cN2: 27 (15),  cN3: 0 (0) |
| ESOPEC | pFLOT | Esophagus: 221 (100) | Adenocarcinoma: 221 (100) | 180 (94) | 32 (17) | | cT1/T2: 43 (20),  cT3/T4: 175 (79) | cN0: 49 (22),  cN+: 172 (78) |
|  | nCROSS | Esophagus: 217 (100) | Adenocarcinoma: 217 (100) | 171 (95) | 18 (10) | | cT1/T2: 37 (17),  cT3/T4: 178 (82) | cN0: 40 (18),  cN+: 177 (82) |

Abbreviations**:** N: number of patients; cT staging: clinical tumor staging; cN staging: clinical lymph-node staging; GEJ: gastroesophageal junction; pFLOT: perioperative fluorouracil, leucovorin, oxaliplatin, and docetaxel; pECF/ECX: perioperative epirubicin, cisplatin, and fluorouracil/capecitabine; pPembro+CF/CX: perioperative pembrolizumab with cisplatin and fluorouracil/capecitabine; pCF/CX: perioperative cisplatin and fluorouracil/capecitabine; pECF: perioperative epirubicin, cisplatin, and fluorouracil; pCF: perioperative cisplatin and fluorouracil; pDurva+FLOT: perioperative durvalumab with fluorouracil, leucovorin, oxaliplatin and docetaxel; pECF/ECX/FLOT+nCRT(F/X): either perioperative epirubicin with cisplatin and fluorouracil/capecitabine, or perioperative fluorouracil, leucovorin, oxaliplatin, and docetaxel, along with neoadjuvant chemoradiotherapy (fluorouracil/capecitabine and radiotherapy); pECF/ECX/FLOT: perioperative epirubicin, cisplatin and fluorouracil/capecitabine, or fluorouracil, leucovorin, oxaliplatin, and docetaxel; nECX+aCRT(CX): neoadjuvant epirubicin, cisplatin/oxaliplatin and capecitabine, followed by adjuvant chemoradiotherapy with radiotherapy, cisplatin and capecitabine; pECX: perioperative epirubicin, cisplatin/oxaliplatin and capecitabine; nCROSS: neoadjuvant paclitaxel and carboplatin with radiotherapy; nCRT(CF): neoadjuvant cisplatin and fluorouracil with radiotherapy; nPLF+nCRT (EP): neoadjuvant cisplatin, leucovorin, and fluorouracil for induction, followed by etoposide and cisplatin with concomitant radiotherapy; nPLF: neoadjuvant cisplatin, leucovorin, and fluorouracil; nECX: neoadjuvant epirubicin, cisplatin, and capecitabine; nCF: neoadjuvant cisplatin and fluorouracil: nCROSS+nivo: neoadjuvant paclitaxel and carboplatin with radiotherapy, plus nivolumab; NA: Not available

^a^ pECF was the predominant choice of chemotherapy administered in this group. However, 27 (15%) patients in the perioperative chemotherapy group received pFLOT instead after a protocol amendment.

# **Supplementary Table 2: Summary of adverse events**

| **Trial** | **Arm** | **Total Patients** | **Grade ≥3** | | | | | | | | | **Grade ≥5** |
| --- | --- | --- | --- | --- | --- | --- | --- | --- | --- | --- | --- | --- |
|  |  |  | **Any adverse**  **events \| N (%)** | **Treatment-related**  **adverse events \| N (%)** | **Diarrhea \| N (%)** | **Nausea \| N (%)** | **Vomiting \| N (%)** | **Neutropenia \| N (%)** | **Infection \| N (%)** | **Peripheral Neuropathy \| N (%)** | **Neutropenic Fever \| N (%)** | **Any adverse events \| N (%)** |
| **Perioperative Setting** | | | | | | | | | | | | |
| FLOT4 | pFLOT | 354 | NA | NA | 34 (10) | 26 (7) | 7 (2) | 181 (51) | 63 (18) | 24 (7) | NA | 2 (0.6) |
|  | pECF/ECX | 354 | NA | NA | 13 (4) | 55 (16) | 27 (8) | 139 (39) | 30 (8) | 7 (2) | NA | 2 (0.6) |
| KEYNOTE-585 | pPembro+CF/CX | 399 | 312 (78) | 259 (65) | 15 (4) | 24 (6) | 14 (4) | 70 (18) | 1 (0·3) | 1 (0.3) | 5 (1) | 4 (1) |
|  | Placebo+pCF/CX | 400 | 297 (74) | 252 (63) | 16 (4) | 22 (6) | 17 (4) | 68 (17) | 0 (0) | 0 (0) | 14 (4) | 2 (0.5) |
| MAGIC | pECF | 250 | NA | NA | NA | NA | NA | NA | NA | NA | NA | NA |
|  | Surgery alone | 253 | NA | NA | NA | NA | NA | NA | NA | NA | NA | NA |
| FNCLCC/FFCD Study | pCF | 109 | 41 (36) | NA | 2 (2) | 10 (9) | | 22 (20) | NA | NA | NA | 1 (1) |
|  | Surgery alone | 111 | NA | NA | NA | NA | NA | NA | NA | NA | NA | 1 (1) |
| MATTERHORN | pDurva+FLOT | 474 | 326 (69) | 275 (58) | NA | NA | NA | NA | NA | NA | NA | NA |
|  | Placebo+pFLOT | 474 | 317 (67) | 264 (56) | NA | NA | NA | NA | NA | NA | NA | NA |
| TOPGEAR | pECF/ECX/FLOT+nCRT(F/X) | 259 | 172 (66) | NA | 23 (9) | 28 (11) | 18 (7) | 101 (39) | 15 (6) | NA | 19 (7) | NA |
|  | pECF/ECX/FLOT | 287 | 176 (61) | NA | 28 (10) | 24 (8) | 20 (7) | 93 (32) | 15 (5) | NA | 26 (9) | NA |
| CRITICS | nECX+aCRT(CX) | 395 | NA | NA | NA | NA | NA | NA | NA | NA | NA | NA |
|  | pECX | 393 | NA | NA | NA | NA | NA | NA | NA | NA | NA | NA |
| **Neoadjuvant Setting** | | | | | | | | | | | | |
| CROSS | nCROSS | 171 | NA | NA | 2 (1) | 2 (1) | 1 (0.6) | 4 (2) | NA | NA | 1 (0.6) | 8 (4) |
|  | Surgery alone | 186 | NA | NA | NA | NA | NA | NA | NA | NA | NA | 7 (4) |
| Burmeister BH et al | nCRT(CF) | 123 | NA | NA | 1 (0.8) | 6 (5) | | 5 (4) | 3 (2) | NA | NA | 0 (0) |
|  | Surgery alone | 128 | NA | NA | NA | NA | NA | NA | NA | NA | NA | NA |
| POET | nPLF+nCRT(EP) | 60 | NA | NA | NA | NA | NA | NA | NA | NA | NA | 6 (10) |
|  | nPLF | 59 | NA | NA | NA | NA | NA | NA | NA | NA | NA | 4 (7) |
| UK MRC OE05 | nECX | 441 | 218 (49) | NA | 36 (8) | 27 (6) | 26 (6) | 101 (23) | 14 (3) | 3 (0.7) | 14 (3) | NA |
|  | nCF | 446 | 140 (31) | NA | 6 (1) | 16 (4) | 20 (4) | 74 (17) | 3 (0.7) | 1 (0.2) | 3 (0.7) | NA |
| MRC OE02 | nCF | 400 | NA | NA | NA | NA | NA | NA | NA | NA | NA | NA |
|  | Surgery alone | 402 | NA | NA | NA | NA | NA | NA | NA | NA | NA | NA |
| EA2174 | nCROSS+Nivo | 137 | NA | NA | NA | NA | NA | NA | NA | NA | NA | 2 (1) |
|  | nCROSS | 138 | NA | NA | NA | NA | NA | NA | NA | NA | NA | 3 (2) |
| **Perioperative with Neoadjuvant Setting** | | | | | | | | | | | | |
| Neo-AEGIS | pECF | 183 | 71 (39) | NA | 20 (11) | 10 (5) | 14 (8) | 49 (27) | 15 (8) | 4 (2) | NA | 6 (3) |
|  | nCROSS | 178 | 57 (32) | NA | 0 (0) | 8 (4) | 5 (3) | 11 (6) | 16 (9) | 0 (0) | NA | 8 (4) |
| ESOPEC | pFLOT | 221 | 120 (54) | NA | 14 (6) | 8 (4) | 7 (3) | 41 (19) | 5 (2) | NA | NA | NA |
|  | nCROSS | 217 | 98 (45) | NA | 0 (0) | 0 (0) | 1 (0.5) | 4 (2) | 2 (0.9) | NA | NA | NA |

Abbreviations**:** N: number of patients; pFLOT: perioperative fluorouracil, leucovorin, oxaliplatin, and docetaxel; pECF/ECX: perioperative epirubicin, cisplatin, and fluorouracil/capecitabine; pPembro+CF/CX: perioperative pembrolizumab with cisplatin and fluorouracil/capecitabine; pCF/CX: perioperative cisplatin and fluorouracil/capecitabine; pECF: perioperative epirubicin, cisplatin, and fluorouracil; pCF: perioperative cisplatin and fluorouracil; pDurva+FLOT: perioperative durvalumab with fluorouracil, leucovorin, oxaliplatin and docetaxel; pECF/ECX/FLOT+nCRT(F/X): either perioperative epirubicin with cisplatin and fluorouracil/capecitabine, or perioperative fluorouracil, leucovorin, oxaliplatin, and docetaxel, along with neoadjuvant chemoradiotherapy (fluorouracil/capecitabine and radiotherapy); pECF/ECX/FLOT: perioperative epirubicin, cisplatin and fluorouracil/capecitabine, or fluorouracil, leucovorin, oxaliplatin, and docetaxel; nECX+aCRT(CX): neoadjuvant epirubicin, cisplatin/oxaliplatin and capecitabine, followed by adjuvant chemoradiotherapy with radiotherapy, cisplatin and capecitabine; pECX: perioperative epirubicin, cisplatin/oxaliplatin and capecitabine; nCROSS: neoadjuvant paclitaxel and carboplatin with radiotherapy; nCRT(CF): neoadjuvant cisplatin and fluorouracil with radiotherapy; nPLF+nCRT (EP): neoadjuvant cisplatin, leucovorin, and fluorouracil for induction, followed by etoposide and cisplatin with concomitant radiotherapy; nPLF: neoadjuvant cisplatin, leucovorin, and fluorouracil; nECX: neoadjuvant epirubicin, cisplatin, and capecitabine; nCF: neoadjuvant cisplatin and fluorouracil: nCROSS+nivo: neoadjuvant paclitaxel and carboplatin with radiotherapy, plus nivolumab; NA: Not available

# **Supplementary Table 3: GRADE summary of findings table outlining certainty of evidence and absolute risks with nCROSS and pFLOT compared to other treatment regimens for overall survival and disease-free survival in the overall population**

| **Comparator** | **nCROSS** | | | **pFLOT** | | |  |
| --- | --- | --- | --- | --- | --- | --- | --- |
|  | **Overall Survival** | | **Disease Free Survival** | **Overall Survival** | **Disease Free Survival** | |  |
| **nCF** OS: 715 per 1000 DFS: 769 per 1000 | **4 more per 1000 (from 120 fewer to 119 more)** | | **18 fewer per 1000 (from 112 fewer to 71 more)** | **110 fewer per 1000 (from 236 fewer to 17 more)** | **160 fewer per 1000 (from 264 fewer to 57 fewer)** | |  |
|  | HR: 1.01 (0.72-1.43) | | HR: 0.95 (0.73-1.25) | HR: 0.74 (0.52-1.05) | HR: 0.64 (0.48-0.85) | |  |
|  | 1304 patients (6 RCTs) | | 1439 patients (6 RCTs) | 1352 patients (5 RCTs) | 1487 patients (5 RCTs) | |  |
|  | Rank: 8 | | Rank: 10 | Rank: 8 | Rank: 10 | |  |
| **nCRT (CF)** OS: not reported ^a^ DFS: 735 per 1000 | **Not estimable ^a^** | | **130 fewer per 1000 (from 271 fewer to 17 more)** | **Not estimable ^a^** | **271 fewer per 1000  (from 398 fewer to 124 fewer)** | |  |
|  | HR: 0.75 (0.50-1.12) | | HR: 0.70 (0.47-1.05) | HR: 0.55 (0.36-0.83) | HR: 0.47 (0.31-0.71) | |  |
|  | 609 patients (4 RCTs) | | 612 patients (4 RCTs) | 657 patients (3 RCTs) | 660 patients (3 RCTs) | |  |
|  | Rank: 11 | | Rank: 11 | Rank: 11 | Rank: 11 | |  |
| **nCROSS** OS: 573 per 1000 DFS: 571 per 1000 | Rank: 9 | | Rank: 9 | **110 fewer per 1000 (from 173 fewer to 42 fewer)** | **138 fewer per 1000 (from 194 fewer to 75 fewer)** | |  |
|  |  |  |  | HR: 0.73 (0.60-0.89) | HR: 0.67 (0.56-0.81) | |  |
|  |  |  |  | 1106 patients (4 RCTs) | 1106 patients (4 RCTs) | |  |
|  |  |  |  | Rank: 9 | Rank: 9 | |  |
| **nECX** OS: 677 per 1000 DFS: 709 per 1000 | **44 more per 1000 (from 96 fewer to 166 more)** | | **37 more per 1000 (from 72 fewer to 136 more)** | **73 fewer per 1000 (from 208 fewer to 71 more)** | **105 fewer per 1000 (from 222 fewer to 11 more)** | |  |
|  | HR: 1.13 (0.77-1.64) | | HR: 1.11 (0.82-1.51) | HR: 0.82 (0.56-1.22) | HR: 0.75 (0.54-1.03) | |  |
|  | 975 patients (4 RCTs) | | 975 patients (4 RCTs) | 1023 patients (3 RCTs) | 1023 patients (3 RCTs) | |  |
|  | Rank: 4 | | Rank: 5 | Rank: 4 | Rank: 5 | |  |
| **nPLF+nCRT (EP)** OS: 617 per 1000  DFS: not reported ^b^ | **159 more per 1000 (from 43 fewer to 309 more)** | | **Not estimable ^b^** | **48 more per 1000  (153 fewer to 236 more)** | **Not estimable ^b^** | |  |
|  | HR: 1.56 (0.89-2.71) | | HR: 1.49 (0.85-2.63) | HR: 1.14 (0.65-2.00) | HR: 1.00 (0.56-1.79) | |  |
|  | 589 patients (4 RCTs) | | 589 patients (4 RCTs) | 637 patients (3 RCTs) | 637 patients (3 RCTs) | |  |
|  | Rank: 1 | | Rank: 2 | Rank: 1 | Rank: 2 | |  |
| **pCF** OS: 491 per 1000 DFS: 559 per 1000 | **43 more per 1000 (from 86 fewer to 183 more)** | | **35 more per 1000 (from 96 fewer to 171 more)** | **62 fewer per 1000 (from 181 fewer to 73 more)** | **105 fewer per 1000 (from 223 fewer to 31 more)** | |  |
|  | HR: 1.13 (0.77-1.66) | | HR: 1.10 (0.76-1.60) | HR: 0.83 (0.55-1.23) | HR: 0.74 (0.50-1.09) | |  |
|  | 1044 patients (5 RCTs) | | 1044 patients (5 RCTs) | 1092 patients (4 RCTs) | 1092 patients (4 RCTs) | |  |
|  | Rank: 5 | | Rank: 8 | Rank: 5 | Rank: 8 | |  |
| **pECF/ECX** OS: 552 per 1000 DFS: 583 per 1000 | **11 more per 1000 (from 57 fewer to 81 more)** | | **38 more per 1000 (from 26 fewer to 102 more)** | **100 fewer per 1000 (from 155 fewer to 41 fewer)** | **106 fewer per 1000 (from 159 fewer to 46 fewer)** | |  |
|  | HR: 1.03 (0.85-1.25) | | HR: 1.11 (0.93-1.32) | HR: 0.75 (0.63-0.89) | HR: 0.74 (0.63-0.88) | |  |
|  | 2004 patients (7 RCTs) | | 2004 patients (7 RCTs) | 2052 patients (6 RCTs) | 2052 patients (6 RCTs) | |  |
|  | Rank: 6 | | Rank: 6 | Rank: 6 | Rank: 6 | |  |
| **pECF/ECX/FLOT**  **+nCRT(F/X)** OS: 535 per 1000 DFS: 570 per 1000 | **7 fewer per 1000 (from 107 fewer to 101 more)** | | **45 more per 1000 (from 58 fewer to 148 more)** | **113 fewer per 1000 (from 200 fewer to 18 fewer)** | **97 fewer per 1000 patients**  **(from 183 fewer to 0 fewer)** | |  |
|  | HR: 0.98 (0.73-1.32) | | HR: 1.13 (0.85-1.50) | HR: 0.72 (0.54-0.95) | HR: 0.76 (0.58-1.00) | |  |
|  | 815 patients (4 RCTs) | | 815 patients (4 RCTs) | 863 patients (3 RCTs) | 863 patients (3 RCTs) | |  |
|  | Rank: 10 | | Rank: 4 | Rank: 10 | Rank: 4 | |  |
| **pFLOT** OS: 456 per 1000 DFS: 520 per 1000 | **110 more per 1000  (from 38 more to 184 more)** | | **145 more per 1000 (from 75 more to 213 more)** | Rank: 2 | Rank: 1 | |  |
|  | HR: 1.37 (1.12-1.68) | | HR: 1.49 (1.23-1.80) |  |  |  |  |
|  | 1106 patients (4 RCTs) | | 1106 patients (4 RCTs) |  |  |  |  |
|  | Rank: 2 | | Rank: 1 |  |  |  |  |
| **pPembro+CF/CX** OS: 410 per 1000 DFS: 455 per 1000 | **76 more per 1000  (from 62 fewer to 233 more)** | | **107 more per 1000 (from 38 fewer to 260 more)** | **25 fewer per 1000 (from 146 fewer to 122 more)** | **31 fewer per 1000 (from 154 fewer to 117 more)** | |  |
|  | HR: 1.26 (0.81-1.95) | | HR: 1.36 (0.89-2.07) | HR: 0.92 (0.58-1.44) | HR: 0.91 (0.59-1.40) | |  |
|  | 931 patients (4 RCTs) | | 931 patients (4 RCTs) | 979 patients (3 RCTs) | 979 patients (3 RCTs) | |  |
|  | Rank: 3 | | Rank: 3 | Rank: 3 | Rank: 3 | |  |
| **nECX+aCRT(CX)**  OS: 582 per 1000  DFS: 610 per 1000 | **7 more per 1000**  **(from 88 fewer to 105 more)** | | **35 more per 1000**  **(from 59 fewer to 127 more)** | **102 fewer per 1000**  **(from 185 fewer to 15 fewer)** | **108 fewer per 1000 patients**  **(from 195 fewer to 23 fewer)** | |  |
|  | HR: 1.02 (0.78-1.33) | | HR: 1.1 (0.85-1.42) | HR: 0.75 (0.58-0.96) | HR: 0.74 (0.57-0.94) | |  |
|  | 924 patients (4 RCTs) | | 924 patients (4 RCTs) | 972 patients (3 RCTs) | 972 patients (3 RCTs) | |  |
|  | Rank: 7 | | Rank: 7 | Rank: 7 | Rank: 7 | |  |
| **Surgery** OS: 740 per 1000 DFS: 781 per 1000 | **90 fewer per 1000 (from 168 fewer to 14 fewer)** | | **116 fewer per 1000 (from 195 fewer to 44 fewer)** | **204 fewer per 1000 (from 285 fewer to 119 fewer)** | **263 fewer per 1000 (from 343 fewer to 183 fewer)** | |  |
|  | HR: 0.78 (0.63-0.96) | | HR: 0.72 (0.58-0.88) | HR: 0.57 (0.45-0.72) | HR: 0.48 (0.38-0.60) | |  |
|  | 1380 patients (7 RCTs) | | 1514 patients (7 RCTs) | 1428 patients (7 RCTs) | 1562 patients (7 RCTs) | |  |
|  | Rank: 12 | | Rank: 12 | Rank: 12 | Rank: 12 | |  |
| **High Certainty Benefit** | | **Low Certainty Benefit** | **Low Certainty No Difference** | **High Certainty Harm** | **Low Certainty Harm** | |  |
|  |  |  |  |  |  |  |  |
| How to read this table: Values in cells represent relative and absolute effect estimates for comparisons between CROSS/FLOT (columns) and other treatment options (rows). Each comparison consists of the absolute risk difference in events (upper cell) with CROSS/FLOT compared to other treatments, relative effect - hazard ratios (upper middle cell), sample size contributive to the evidence (lower middle cell), and relative rank of the treatment in a row (lower cell). | | | | | |  |  |

Abbreviations: OS: overall survival; DFS: disease-free survival; HR: hazard ratio; nCF: neoadjuvant cisplatin and fluorouracil; nCRT (CF): neoadjuvant cisplatin and fluorouracil with radiotherapy; nCROSS: neoadjuvant paclitaxel and carboplatin with radiotherapy; nECX: neoadjuvant epirubicin, cisplatin, and capecitabine; nPLF+nCRT (EP): neoadjuvant cisplatin, leucovorin, and fluorouracil for induction, followed by etoposide and cisplatin with concomitant radiotherapy; pCF: perioperative cisplatin and fluorouracil; pECF/ECX: perioperative epirubicin, cisplatin, and fluorouracil/capecitabine; pECF/ECX/FLOT+nCRT(F/X): either perioperative epirubicin with cisplatin and fluorouracil/capecitabine, or perioperative fluorouracil, leucovorin, oxaliplatin, and docetaxel, along with neoadjuvant chemoradiotherapy (fluorouracil/capecitabine and radiotherapy); pFLOT: perioperative fluorouracil, leucovorin, oxaliplatin, and docetaxel; pPembro+CF/CX: perioperative pembrolizumab with cisplatin and fluorouracil/capecitabine; nECX+aCRT(CX): neoadjuvant epirubicin, cisplatin/oxaliplatin and capecitabine, followed by adjuvant chemoradiotherapy with radiotherapy, cisplatin and capecitabine

^a^ Total deaths with nCRT (CF) were not reported in the Burmeister et al trial ^30^ , therefore, the baseline risk for all-cause mortality could not be calculated between the treatment (nCROSS/pFLOT) and the comparator (nCRT (CF)). Consequently, the absolute risk difference in all-cause mortality was not estimated.

^b^ Events for disease progression with nPLF+nCRT (EP) were not reported in the POET trial ^31^, therefore, the baseline risk for disease progression could not be calculated between the treatment (nCROSS/pFLOT) and the comparator (nPLF+nCRT (EP)). Consequently, the absolute risk difference in disease progression was not estimated.

# **Supplementary Table 4: GRADE summary of findings table outlining certainty of evidence and absolute risks with nCROSS and pFLOT compared to other treatment regimens for overall survival and disease-free survival in the GEJ cohort**

| **Comparator** | **nCROSS** | | **pFLOT** | |  |  |  |
| --- | --- | --- | --- | --- | --- | --- | --- |
|  | **Overall Survival** | **Disease Free Survival** | **Overall Survival** | **Disease Free Survival** |  |  |  |
| **nCF** OS: 705 per 1000 | **14 more per 1000  (from 120 fewer to 133 more)** | NA | **105 fewer per 1000 (255 fewer to 40 more)** | NA |  |  |  |
|  | HR: 1.04 (0.72-1.49) |  | HR: 0.75 (0.49-1.12) |  |  |  |  |
|  | 848 patients (5 RCTs) |  | 658 patients (4 RCTs) |  |  |  |  |
|  | Rank: 6 |  | Rank: 6 |  |  |  |  |
| **nCROSS** OS: 573 per 1000 DFS: 527 per 1000 | Rank: 8 | Rank: 4 | **120 fewer per 1000 (from 189 fewer to 42 fewer)** | **137 fewer per 1000 (from 210 fewer to 56 fewer)** |  |  |  |
|  |  |  | HR: 0.71 (0.57-0.89) | HR: 0.66 (0.51-0.85) |  |  |  |
|  |  |  | 868 patients (4 RCTs) | 794 patients (3 RCTs) |  |  |  |
|  |  |  | Rank: 8 | Rank: 4 |  |  |  |
| **nCRT(CF)**  DFS: 778 per 1000 | NA | **121 fewer per 1000**  **(from 278 fewer to 28 more)** | NA | **271 fewer per 1000 patients**  **(from 434 fewer to 87 fewer)** |  |  |  |
|  |  | HR: 0.71 (0.46-1.09) |  | HR: 0.47 (0.28-0.78) |  |  |  |
|  |  | 672 patients (4 RCTs) |  | 320 patients (2 RCTs) |  |  |  |
|  |  | Rank: 5 |  | Rank: 5 |  |  |  |
| **nPLF+nCRT (EP)**  OS: 617 per 1000 | **168 more per 1000**  **(from 39 fewer to 317 more)** | NA | **51 more per 1000**  **(163 fewer to 247 more)** | NA |  |  |  |
|  | HR: 1.60 (0.90-2.83) |  | HR: 1.15 (0.63-2.08) |  |  |  |  |
|  | 589 patients (4 RCTs) |  | 399 patients (3 RCTs) |  |  |  |  |
|  | Rank: 1 |  | Rank: 1 |  |  |  |  |
| **pCF** OS: 671 per 1000 | **101 more per 1000 (from 64 fewer to 231 more)** | NA | **19 fewer per 1000 (from 196 fewer to 152 more)** | NA |  |  |  |
|  | HR: 1.33 (0.84-2.09) |  | HR: 0.95 (0.58-1.56) |  |  |  |  |
|  | 678 patients (5 RCTs) |  | 488 patients (4 RCTs) |  |  |  |  |
|  | Rank: 3 |  | Rank: 3 |  |  |  |  |
| **pECF/ECX** OS: 550 per 1000  DFS: 488 per 1000 | **14 more per 1000 (from 61 fewer to 93 more)** | **40 more per 1000**  **(from 50 fewer to 138 more)** | **100 fewer per 1000 (from 171 fewer to 30 fewer)** | **97 fewer per 1000 patients**  **(from 199 fewer to 27 more)** |  |  |  |
|  | HR: 1.04 (0.84-1.29) | HR: 1.12 (0.86-1.47) | HR: 0.75 (0.60-0.92) | HR: 0.74 (0.51-1.08) |  |  |  |
|  | 1062 patients (6 RCTs) | 858 patients (4 RCTs) | 872 patients (6 RCTs) | 506 patients (3 RCTs) |  |  |  |
|  | Rank: 7 | Rank: 3 | Rank: 7 | Rank: 3 |  |  |  |
| **pECF/ECX/FLOT+nCRT (F/X)**  OS: 561 per 1000  DFS: 582 per 1000 | **104 more per 1000**  **(from 46 fewer to 249 more)** | **162 more per 1000**  **(from 0 to 297 more)** | **18 fewer per 1000 patients**  **(from 156 fewer to 136 more)** | **11 more per 1000 patients**  **(from 164 fewer to 195 more)** |  |  |  |
|  | HR: 1.33 (0.88-2.02) | HR: 1.56 (1.00-2.42) | HR: 0.95 (0.63-1.45) | HR: 1.03 (0.62-1.72) |  |  |  |
|  | 627 patients (4 RCTs) | 671 patients (4 RCTs) | 437 patients (3 RCTs) | 319 patients (2 RCTs) |  |  |  |
|  | Rank: 4 | Rank: 1 | Rank: 4 | Rank: 1 |  |  |  |
| **pFLOT** OS: 439 per 1000 DFS: 484 per 1000 | **116 more per 1000 (from 38 more to 195 more)** | **150 more per 1000 (from 55 more to 243 more)** | Rank: 2 | Rank: 2 |  |  |  |
|  | HR: 1.40 (1.12-1.74) | HR: 1.52 (1.17-1.96) |  |  |  |  |  |
|  | 868 patients (4 RCTs) | 794 patients (3 RCTs) |  |  |  |  |  |
|  | Rank: 2 | Rank: 2 |  |  |  |  |  |
| **pPembro+CF/CX** OS: 407 per 1000 | **12 more per 1000**  **(157 fewer to 234 more)** | NA | **86 fewer per 1000**  **(from 227 fewer to 124 more)** | NA |  |  |  |
|  | HR: 1.04 (0.55-1.96) |  | HR: 0.74 (0.38-1.45) |  |  |  |  |
|  | 615 patients (4 RCTs) |  | 425 patients (3 RCTs) |  |  |  |  |
|  | Rank: 5 |  | Rank: 5 |  |  |  |  |
| **nECX+aCRT(CX)**  OS: 567 per 1000 | **26 fewer per 1000 patients**  **(231 fewer to 206 more)** | NA | **138 fewer per 1000 patients**  **(313 fewer to 88 more)** | NA |  |  |  |
|  | HHR: 0.93 (0.49-1.77) |  | HR: 0.67 (0.35-1.27) |  |  |  |  |
|  | 596 patients (4 RCTs) |  | 406 patients (3 RCTs) |  |  |  |  |
|  | Rank: 9 |  | Rank: 9 |  |  |  |  |
| **Surgery** OS: 791 per 1000 DFS: 623 per 1000 | **95 fewer per 1000 (from 188 fewer to 7 fewer)** | **133 fewer per 1000 (from 225 fewer to 31 fewer)** | **220 fewer per 1000 (from 326 fewer to 100 fewer)** | **261 fewer per 1000 (from 362 fewer to 143 fewer)** |  |  |  |
|  | HR: 0.76 (0.59-0.98) | HR: 0.69 (0.52-0.92) | HR: 0.54 (0.40-0.75) | HR: 0.46 (0.31-0.67) |  |  |  |
|  | 1064 patients (6 RCTs) | 865 patients (4 RCTs) | 874 patients (6 RCTs) | 513 patients (3 RCTs) |  |  |  |
|  | Rank: 10 | Rank: 6 | Rank: 10 | Rank: 6 |  |  |  |
|  | **High Certainty Benefit** | **Low Certainty Benefit** | **High Certainty Harm** | **Low Certainty Harm** |  |  |  |
|  |  |  |  |  |  | |  |
| How to read this table: Values in cells represent relative and absolute effect estimates for comparisons between CROSS/FLOT (columns) and other treatment options (rows). Each comparison consists of the absolute risk difference in events (upper cell) with CROSS/FLOT compared to other treatments, relative effect - hazard ratios (upper middle cell), sample size contributive to the evidence (lower middle cell), and relative rank of the treatment in a row (lower cell). | | | | | |  | |

Abbreviations: OS: overall survival; DFS: disease-free survival; HR: hazard ratio; nCF: neoadjuvant cisplatin and fluorouracil; nCROSS: neoadjuvant paclitaxel and carboplatin with radiotherapy; nCRT (CF): neoadjuvant cisplatin and fluorouracil with radiotherapy; nPLF+nCRT (EP): neoadjuvant cisplatin, leucovorin, and fluorouracil for induction, followed by etoposide and cisplatin with concomitant radiotherapy; pCF: perioperative cisplatin and fluorouracil; pECF/ECX: perioperative epirubicin, cisplatin, and fluorouracil/capecitabine; pECF/ECX/FLOT+nCRT(F/X): either perioperative epirubicin with cisplatin and fluorouracil/capecitabine, or perioperative fluorouracil, leucovorin, oxaliplatin, and docetaxel, along with neoadjuvant chemoradiotherapy (fluorouracil/capecitabine and radiotherapy); pFLOT: perioperative fluorouracil, leucovorin, oxaliplatin, and docetaxel; pPembro+CF/CX: perioperative pembrolizumab with cisplatin and fluorouracil/capecitabine; nECX+aCRT(CX): neoadjuvant epirubicin, cisplatin/oxaliplatin and capecitabine, followed by adjuvant chemoradiotherapy with radiotherapy, cisplatin and capecitabin
